# Cryogenic infrared spectroscopy provides mechanistic insight into the fragmentation of phospholipid silver adducts

**DOI:** 10.1007/s00216-022-03927-6

**Published:** 2022-02-11

**Authors:** Carla Kirschbaum, Kim Greis, Sandy Gewinner, Wieland Schöllkopf, Gerard Meijer, Gert von Helden, Kevin Pagel

**Affiliations:** 1 Institut für Chemie und Biochemie, Freie Universität Berlin, 14195 Berlin, Germany; 2 Fritz-Haber-Institut der Max-Planck-Gesellschaft, 14195 Berlin, Germany

**Keywords:** Infrared spectroscopy, Tandem mass spectrometry, Lipidomics, Glycerophospholipids, Isomers, Silver

## Abstract

Tandem mass spectrometry is arguably the most important analytical tool for structure elucidation of lipids and other metabolites. By fragmenting intact lipid ions, valuable structural information such as the lipid class and fatty acyl composition are readily obtainable. The information content of a fragment spectrum can often be increased by the addition of metal cations. In particular, the use of silver ions is deeply rooted in the history of lipidomics due to their propensity to coordinate both electron-rich heteroatoms and C = C bonds in aliphatic chains. Not surprisingly, coordination of silver ions was found to enable the distinction of *sn*-isomers in glycerolipids by inducing reproducible intensity differences in the fragment spectra, which could, however, not be rationalized. Here, we investigate the fragmentation behaviors of silver-adducted *sn*- and double bond glycerophospholipid isomers by probing fragment structures using cryogenic gas-phase infrared (IR) spectroscopy. Our results confirm that neutral headgroup loss from silver-adducted glycerophospholipids leads to dioxolane-type fragments generated by intramolecular cyclization. By combining high-resolution IR spectroscopy and computational modelling of silver-adducted fragments, we offer qualitative explanations for different fragmentation behaviors of glycerophospholipid isomers. Overall, the results demonstrate that gas-phase IR spectroscopy of fragment ions can significantly contribute to our understanding of lipid dissociation mechanisms and the influence of coordinating cations.

**Graphical abstract:**

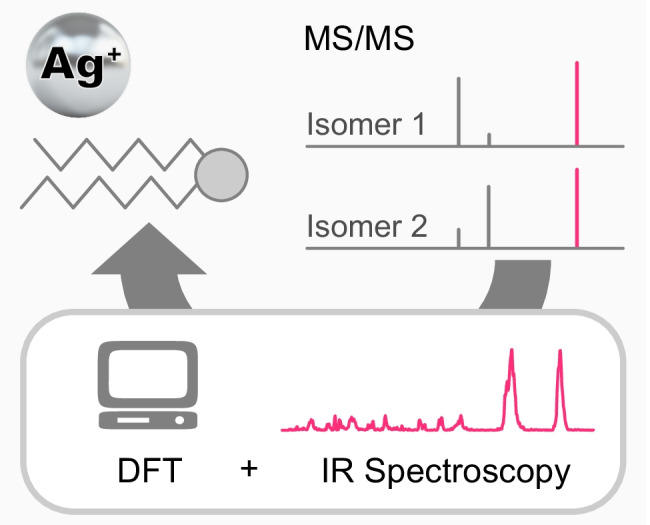

**Supplementary Information:**

The online version contains supplementary material available at 10.1007/s00216-022-03927-6.

## Introduction

**Fig. 1:**
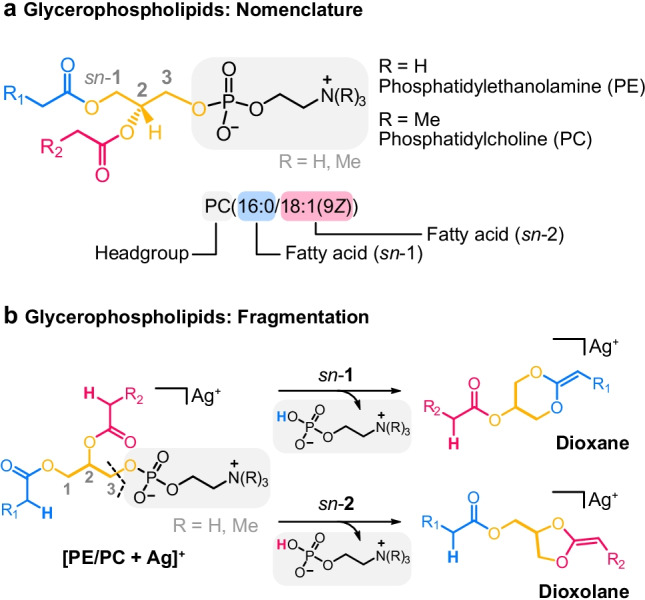
Structure and fragmentation of glycerophospholipids. **a** Glycerophospholipids feature a phosphate-containing headgroup and a glycerol backbone (yellow) esterified with two fatty acyls (blue and red, abbreviated by R_1_ and R_2_). The shorthand notation includes information on the headgroup and the position, length, and unsaturation of the two fatty acyls. **b** Neutral headgroup loss is the predominant fragmentation pathway of silver-adducted phospholipids. The resulting fragment structure can feature a dioxane or dioxolane ring resulting from *sn*-1 or *sn*-2 fatty acyl participation, respectively

Glycerophospholipids constitute the major components of mammalian membranes [1]. As such, they do not only influence membrane properties but also membrane protein functions and cellular signaling [1, 2]. Each mammalian cell contains a number of different glycerophospholipid classes, among which phosphatidylcholines (PCs) and phosphatidylethanolamines (PEs) are most abundant [3]. PC and PE differ by the phosphate-containing headgroup attached to the *sn*-3 carbon of the glycerol backbone, which is common to all glycerophospholipids and numbered using the stereospecific numbering (*sn*) nomenclature from *sn*-1 to *sn*-3 (Fig. 1). The remaining *sn*-1 and *sn*-2 positions are esterified with fatty acyls of variable length and degree of unsaturation. In general, glycerophospholipid structures can be described on five levels of complexity [4]. Ordered by increasing analytical challenge, the levels of structure information include (1) the glycerophospholipid class, (2) the fatty acyl sum composition, i.e., length and degree of unsaturation of the aliphatic chains, (3) the relative position of the two fatty acyls on the glycerol backbone (*sn*-position), (4) the location of C = C bonds within the fatty acyl chains, and (5) the stereochemistry of C = C bonds (*Z* or *E*). The shorthand nomenclature for glycerophospholipids employed throughout this work captures all five levels of complexity in a condensed form and is based on the recommendations by the LIPID MAPS Consortium [5, 6].

Complete structural characterization of glycerophospholipids requires advanced tandem mass spectrometry (MS) techniques involving derivatization strategies, alternative ion activation methods, and combinations of MS with optical spectroscopy [7]. Nonetheless, the first two levels of complexity can be readily tackled by collision-induced dissociation (CID), which is the most widespread ion activation method for routine tandem MS analyses. In negative ion mode, CID generates carboxylate anions that reveal the fatty acyl identities, whereas in positive ion mode, diagnostic ions resulting from headgroup cleavage determine the glycerophospholipid class [8]. Neutral headgroup loss in positive ion mode was postulated to proceed via an intramolecular cyclization resulting in a six-membered dioxane ring or five-membered dioxolane ring [9]. Subsequent studies confirmed the dioxolane structure for alkali metal adducts [4, 10–12] and protonated glycerolipid fragments [13]. Spectroscopic investigations of phospholipid precursor ions explained the formation of dioxolanes by strong interactions between the *sn*-1 ester and the cation, promoting intramolecular cyclization of the less strongly bound *sn*-2 ester into a five-membered ring [12]. In the cyclic dioxolane structure, the fatty acyls occupy chemically different positions, either being part of the ring or next to the ring. This advantage is exploited to assign *sn*-isomers by advanced tandem MS methods [4, 10, 11]. In principle, the positions of the lipid chains on the glycerol backbone can also be determined in CID experiments by monitoring peak intensity differences of fragments resulting from fatty acid loss [14]. However, no fragment ion is exclusive for one *sn*-isomer because the dissociation of fatty acyls from the *sn*-1 and *sn*-2 position is competing. Hence, product ion intensities of carboxylate anions and alkali metal adducts were found to depend on instrument parameters [15] and on the nature of the phospholipid headgroup [16]. As a result, peak abundances alone are not sufficient to assign acyl chain positions in glycerophospholipids without calibration. Transition metal ions were found to yield more abundant *sn*-specific product ions and more reproducible spectra [16, 17]. For example, silver adduction yields CID spectra that allow for the distinction of *sn*-isomers based on the ratio of ketene and acid fragments, independent of the phospholipid class [18]. Historically, silver ions have played a special role in lipid analysis and separation. Silver ion chromatography separates lipids according to the number, position, and stereochemistry of C = C bonds [19] based on the ability of silver ions to bind lone pairs of heteroatoms and pi-bonds [20]. The strong interaction between silver ions and pi-bonds can be explained based on the concept of hard and soft acids and bases (HSAB), according to which soft acids such as Ag^+^ preferably bind to soft bases such as pi-electrons of C = C bonds [21]. Such cation-pi interactions are also expected in the gas phase and allowed for the separation of glycerophospholipid *sn*- [22] and double bond isomers [23] by ion mobility spectrometry (IMS) and subsequent acquisition of isomerically pure tandem MS spectra. However, the observed intensity differences between acid and ketene fragments in *sn*-isomers have never been rationalized.

Here, we investigate collision-induced fragmentation of silver-adducted glycerophospholipids by cryogenic IR spectroscopy to understand different fragmentation behaviors of *sn*- and double bond isomers. In combination with density functional theory (DFT) calculations, MS^2^ fragment ion structures were confirmed to feature a five-membered dioxolane ring in which the two fatty acids occupy chemically different positions. The IR spectra are highly dependent on the nature of the lipid chains and the resulting coordination geometry of silver, which is employed to qualitatively explain intensity differences between acid and ketene fragments in the CID spectra of glycerophospholipid isomers.

## Materials and methods

### Reagents and solvents

PE(6:0/6:0), PE(16:0/18:1(9*Z*)), PE(18:1(9*Z*)/16:0), PC(16:0/18:1(9*Z*)), PC(18:1(9*Z*)/18:1(9*Z*)), and PC(18:1(6*Z*)/18:1(6*Z*)) were purchased from Avanti Polar Lipids (Alabaster, USA). Methanol and silver hexafluorophosphate were purchased from Sigma-Aldrich (Taufkirchen, Germany). Phospholipids were dissolved in methanol and diluted to obtain 100 µm solutions containing 1.7 mM Ag[PF_6_]. The solutions were stored at − 25 °C until use.

### Tandem mass spectrometry

Tandem mass spectra were measured on a Synapt G2-S HDMS instrument (Waters Corporation, Manchester). Silver-adducted glycerophospholipids were fragmented in the ion source and the fragments resulting from neutral headgroup loss were *m/z*-selected in the quadrupole before being subjected to CID in the trap cell. Acceleration voltages of 35–40 V were applied.

### Cryogenic gas-phase infrared spectroscopy in helium nanodroplets

Gas-phase IR spectra of lipid ions were recorded on a custom-built instrument described previously [24–26]. Silver adducts of phospholipids were generated by nano-electrospray ionization (nano-ESI) of Ag[PF_6_]-containing phospholipid solutions using Pd/Pt-coated glass capillaries and applying a needle voltage of 0.7–1.1 kV. In the source region of the instrument, the intact precursor ions are subjected to in-source fragmentation induced by ion acceleration and collisions with residual gas molecules (Fig. S1). The fragment ions of interest are subsequently *m/z*-selected in a quadrupole and guided towards a hexapole ion trap. Upon entering the ion trap, the ions are decelerated and thermalized by pre-cooled helium buffer gas (90 K) and trapped by DC and RF potentials. After pumping out the buffer gas, the trapped ions can be picked up by a traversing beam of superfluid helium droplets pulsed by a cryo-cooled Even-Lavie valve (21 K nozzle temperature, 10 Hz repetition rate). Once picked up by a helium droplet, ions are cooled down to the intrinsic droplet temperature of 0.4 K by evaporation of helium atoms. Contrary to the bare ions, ions inside a helium droplet can leave the trap because of the high kinetic energy of the droplet. The doped droplets travel towards the interaction region, where they coincide with a pulsed laser beam generated by the Fritz Haber Institute free-electron laser (FHI FEL) [27]. The FHI FEL provides tunable IR radiation, which is scanned over the wavenumber range of interest in steps of 2 cm^−1^ to record an IR spectrum. If the photon energy is resonant with a vibration of the molecular ion, vibrational energy is dissipated by the evaporation of helium leading to shrinkage of the droplet. After the absorption of multiple photons, the bare ion is eventually released from the droplet and detected by a time-of-flight mass detector. The ion signal on the time-of-flight detector is plotted against the photon wavenumber to obtain an IR spectrum. All spectra shown in this work were averaged over two scans.

### Computational modelling of glycerophospholipid fragments

Computed IR spectra of silver-adducted glycerophospholipid fragments were obtained in a four-step procedure: (1) determination of preferred coordination sites of silver ions on the lipid fragments, (2) a conformational search of silver-adducted lipid fragments using a semiempirical method, (3) geometry optimization of selected conformers by DFT followed by (4) a harmonic frequency calculation of the optimized structures. Silver coordination sites on the phospholipid fragments were determined for both dioxolane and dioxane model structures using CREST [28] with the semiempirical method GFN2-xTB [29] and default settings by entering the keywords *-protonate -swel Ag* + , as described in the Supplementary Information. The most stable coordination geometry was subjected to a conformational search in CREST. Several low-energy conformers were optimized in Gaussian 16 [30] at the PBE0 + D3/6–311 + G(d,p) level of theory including an SDD effective core potential for silver. The geometry optimization was followed by a harmonic frequency calculation at the same level of theory. The harmonic IR spectra were scaled by single-parameter frequency scaling to correct the systematic overestimation of vibrational frequencies within the harmonic approximation. The applied scaling factor was 0.965, in accordance with previous works [13, 31, 32]. Harmonic free energies ΔF were calculated at 90 K, according to the temperature in the ion trap.

Structures of allylic dioxolane fragments were computed following the same procedure at the PBE0 + D3/6–311 + G(d,p) level of theory, with the only difference that step (1) was skipped. Rotation barriers of allylic dioxolane fragments with truncated lipid chains were computed by a relaxed potential energy surface (PES) scan in Gaussian 16. The dihedral angle of interest was incremented in 72 steps of 5°, followed by a geometry optimization and single point energy calculation (Fig. S10). Transition states for the formation of allylic dioxolane fragments from silver-adducted dioxolane model structures were computed by scanning the PES of the C-H bond to be broken in Gaussian 16. The structure at the saddle point of the PES was optimized as a transition state at the PBE0 + D3/6–311 + G(d,p), SDD (Ag) level of theory. A frequency analysis of the structure thus obtained was performed to prove the existence of one imaginary frequency. The transition state was then linked to a reactant and product by an intrinsic reaction coordinate calculation (Fig. S11). XYZ coordinates, energetics, and IR spectra of all computed conformers are available in the Supplementary Information.

## Results

### Neutral headgroup loss from silver-adducted phospholipids

**Fig. 2:**
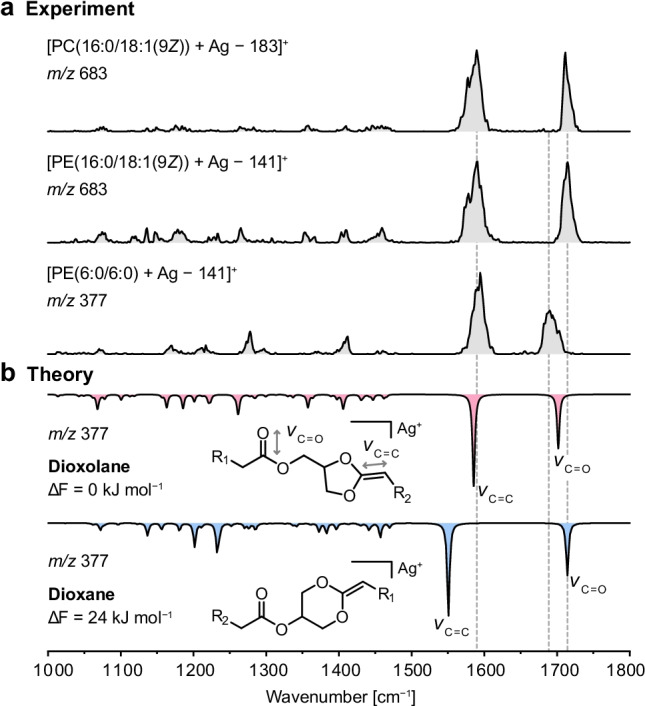
Experimental and computed IR spectra of silver-adducted phospholipid fragments generated by neutral headgroup loss. **a** The band positions in the fragment spectra of PE and PC(16:0/18:1(9*Z*)) are not influenced by the different headgroups. The lipid chains have a major influence on the spectral signature. **b** Computed spectra for [PE(6:0/6:0) + Ag − 141]^+^ show that the fragment features a five-membered dioxolane ring. Spectra were computed at the PBE0 + D3/6–311 + G(d,p), SDD (Ag) level of theory and are shown as inverted traces below the experimental spectra accompanied by their relative free energies (ΔF) at 90 K in kJ mol^−1^

In positive ion mode, CID of silver-adducted glycerophospholipids yields abundant product ions resulting from neutral loss of the phosphate-containing headgroup (Fig. S2). In recent studies, MS^2^ fragments generated by neutral headgroup loss from protonated [13], sodiated [4, 10, 11], and potassiated [12] glycerophospholipids were shown to feature a cyclic dioxolane structure. In order to determine whether silver adduction also yields dioxolane structures and to estimate the respective influence of the headgroup and the lipid chains on the fragment structure, silver-adducted PE and PC precursor ions were subjected to in-source fragmentation (Fig. S1). The fragments generated by neutral headgroup loss were subsequently probed by cryogenic IR spectroscopy. The IR spectra of fragments from silver-adducted PC(16:0/18:1(9*Z*)), PE(16:0/18:1(9*Z*)), and PE(6:0/6:0) are shown in Fig. 2. As the headgroup is cleaved during in-source fragmentation, the fragments resulting from PE and PC precursor ions with the same lipid chains are expected to be identical. Indeed, the IR spectra of [PC(16:0/18:1(9*Z*)) + Ag − 183]^+^ and [PE(16:0/18:1(9*Z*)) + Ag − 141]^+^ fragments are equal except for intensity differences in the lower wavenumber region that can be ascribed to day-to-day variations in laser power. The lipid chains, on the contrary, have a major influence on the IR spectrum, as illustrated by the model lipid PE(6:0/6:0) equipped with short, saturated hexanoic acid chains. One of the two main vibration bands is significantly shifted compared with the IR spectra of the fragments featuring significantly longer lipid chains and one unsaturation. In order to assign the vibration bands and determine the core fragment structure, DFT calculations were performed for both dioxolane and dioxane fragment structures. The preferred coordination geometry of silver ions was found to involve interactions with the carbonyl oxygen and the C = C bond adjacent to the ring (Fig. S3). Geometry optimizations and harmonic frequency calculations of [PE(6:0/6:0) + Ag − 141]^+^ ions were subsequently performed at the PBE0 + D3/6–311 + G(d,p), SDD (Ag) level of theory (Fig. S4). As shown in Fig. 2, the computed dioxolane fragment is energetically favored over the dioxane fragment and yields a satisfactory match with the experimental spectrum. The two main vibration bands are attributed to the stretching vibration of the C = C bond adjacent to the ring around 1600 cm^−1^ and to the C = O stretching vibration of the fatty acid next to the ring approximately at 1700 cm^−1^. The computed IR spectrum of the dioxane fragment does not coincide with the experimental spectrum as the C = C stretching frequency is red-shifted to 1550 cm^−1^ and the C = O vibration is blue-shifted to above 1700 cm^−1^. As a conclusion, MS^2^ fragments resulting from neutral headgroup loss from silver-adducted PE and PC ions yield exclusively dioxolane structures, in accordance with alkali metal adducts and protonated fragments. Truncation of the lipid chains leads to a red-shifted C = O stretching vibration in the spectrum of [PE(6:0/6:0) + Ag − 141]^+^ compared with the spectra of fragments featuring longer lipid chains, which is supported by DFT calculations (Figs. S4–S5). In addition, the vibration at 1600 cm^−1^ is broader and slightly shifted in the case of longer lipid chains. DFT calculations suggest that the stretching vibration of the C = C bond in the lipid chain that interacts with the silver ion contributes to the vibration band at 1600 cm^−1^ and leads to broadening compared to the truncated lipid chains lacking double bonds (compare Fig. S4 and Fig. S5). In the case of protonated dioxolane fragments that were previously studied by cryogenic IR spectroscopy, the band positions are not affected by the nature of the lipid chains [13]. Band shifts due to silver adduction hint to interactions between the silver ion and the fatty acyls, which influences the conformation of the fragment ions and consequently the spectral signature. In the following, silver-lipid interactions are studied in more detail to investigate the effect of lipid chain permutation on the glycerol backbone and C = C bond positions within the lipid chains on the fragment geometry and fragmentation behavior.

### Collision-induced dissociation of *sn*-isomers

**Fig. 3:**
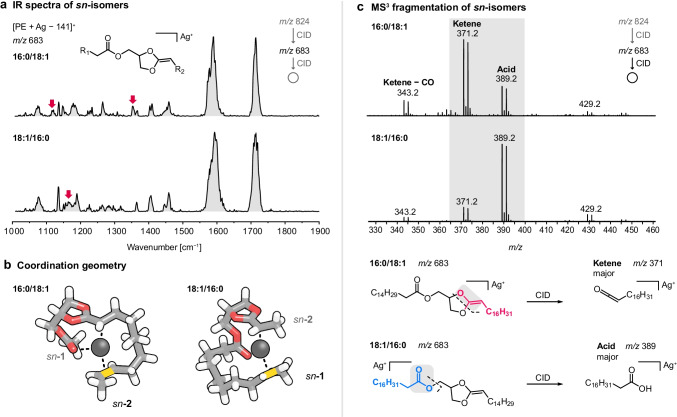
Collision-induced dissociation of *sn*-isomers. **a** The IR spectra of dioxolane fragments generated from PE(16:0/18:1(9*Z*)) and PE(18:1(9*Z*)/16:0) display subtle differences in the lower wavenumber region (1000–1500 cm^−1^). **b** Computed structures of dioxolane fragments show that silver coordinates to the C = C bond in the oleic acid residue. The fatty acyls are truncated for visibility and the double bond in oleic acid is highlighted in yellow. **c** Inverse ratios of acid and ketene fragments depending on the position of the fatty acyls can be rationalized by the preferential coordination of silver to the oleic acid residue and the preformed acid and ketene motifs at the *sn*-1 and *sn*-2 positions, respectively

Permutation of two different lipid chains on the glycerol backbone yields *sn*-isomers, which can have distinct properties and functions [22]. Silver adduction has been shown to generate distinguishable MS^3^ spectra for *sn*-isomers featuring palmitic acid (16:0) and oleic acid (18:1(9*Z*)) based on different relative abundances of silver-adducted oleic acid fragments in the form of carboxylic acid (*m/z* 389) or ketene (*m/z* 371) [18, 22]. If oleic acid is attached to the *sn*-2 position, ketene formation is prevalent, whereas carboxylic acid fragments are predominant if oleic acid is attached to the *sn*-1 position (Fig. 3c). In order to explain the observed intensity differences in the CID spectra, IR spectra of the MS^2^ fragment ions [PE(16:0/18:1(9*Z*)) + Ag − 141]^+^ and [PE(18:1(9*Z*)/16:0) + Ag − 141]^+^ at *m/z* 683 were recorded (Fig. 3a). The positions of the main absorption bands are identical, whereas minor but reproducible differences are discernible in the lower wavenumber region. It is important to note that commercial phospholipid standards usually contain a few percent up to 19% of the other *sn*-isomer due to acyl chain migration during synthesis [14, 22]. For instance, ion mobility analysis of silver-adducted PC(16:0/18:1(9*Z*)) and PC(18:1(9*Z*)/16:0) standards yielded *sn*-isomer impurities of 13% and 1%, respectively [22]. In line with this result, the IR spectrum of the [PE(18:1(9*Z*)/16:0) + Ag − 141]^+^ almost completely lacks the two absorption bands present in the spectrum of the PE(16:0/18:1(9*Z*)) isomer, whereas the spectrum of the latter is less distinct and probably contains a more considerable amount of *sn*-isomer impurities.

The fragment geometry is expected to be the same for both isomers but distinct coordination motifs between silver and the fatty acyls might induce conformational differences causing minor differences in the lower wavenumber region. According to DFT calculations, the region below 1500 cm^−1^ is dominated by C–H bending vibrations of the lipid chains. The experimental differences between the *sn*-isomers can, however, not be assigned to specific vibrational modes due to the crowdedness of that spectral region and insufficient accuracy of computation for such large and flexible lipids. Overall, the computed IR spectra yield a satisfactory match with the main absorption bands in the experimental spectra (Figs. S5–S6). Interestingly, the computed coordination geometry indeed clearly differs between the two isomers (Fig. 3b). As silver ions preferentially coordinate C = C bonds in fatty acids, the fragment geometry is adapted to enable an interaction between the C = C bond of oleic acid and the silver ion. In the (16:0/18:1(9*Z*)) isomer, oleic acid is attached to the *sn*-2 position and therefore participates in the formation of the dioxolane ring. In the (18:1(9*Z*)/16:0) isomer, oleic acid is attached next to the dioxolane ring and interacts with silver via the C = C bond and the carbonyl oxygen. These different coordination geometries and the dioxolane structure of the MS^2^ fragments are key to explain the previously noted intensity differences between MS^3^ ketene and acid fragments: in the (16:0/18:1(9*Z*)) isomer, the ketene motif is already preformed because oleic acid is part of the dioxolane ring and features a C = C bond between the original carbonyl carbon and the alpha carbon. In contrast, in the (18:1(9*Z*)/16:0) isomer, the oleic acid is esterified next to the dioxolane ring and the carboxylic acid motif is preformed. The preferential loss of oleic acid as either ketene or carboxylic acid can thus be rationalized by the structure and coordination geometry of the MS^2^ intermediate fragment. In both cases, however, a small fraction of the other structural motif is also present, showing that the formations of ketene and acid are competitive.

### Collision-induced dissociation of double bond isomers

**Fig. 4:**
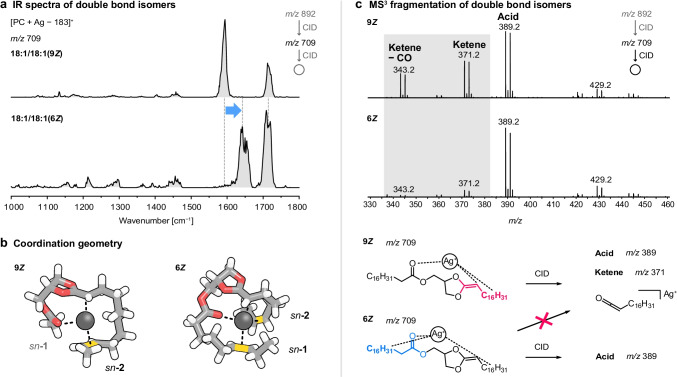
Collision-induced dissociation of double bond positional isomers. **a** The IR spectra of the dioxolane fragments generated from PC(18:1(9*Z*)/18:1(9*Z*)) and PC(18:1(6*Z*)/18:1(6*Z*)) show a significant band shift. **b** Computed structures of dioxolane fragments confirm that silver coordinates only to the fatty acyl at the *sn*-2 position in the 9*Z* isomer but to both lipid chains in the 6*Z* isomer. The fatty acyls are truncated for visibility and double bonds are highlighted in yellow. **c** Ketene formation is suppressed in the fragmentation of dioxolane fragments generated from the 6*Z* isomer. One possible explanation is the strong coordination of silver to the fatty acyl at the *sn*-1 position, which preferentially yields acid fragments

The position of unsaturated lipid chains on the glycerol backbone influences the coordination geometry in silver-adducted glycerophospholipids and thus the fragmentation behavior of *sn*-isomers. As the coordination of silver is mainly dictated by interactions with C = C bonds of unsaturated lipid chains, the position of unsaturations is equally expected to influence the coordination geometry and eventually the fragmentation behavior. To test this hypothesis, the double bond isomers PC(18:1(9*Z*)/18:1(9*Z*)) and PC(18:1(6*Z*)/18:1(6*Z*)) were investigated. The molecules feature identical monounsaturated C18 lipid chains at the *sn*-1 and *sn*-2 positions. In the 9*Z* isomer, the C = C bond is located at the center of the lipid chain, whereas in the 6*Z* isomer, the C = C bond is shifted by three carbon atoms closer to the glycerol backbone. CID of the double bond isomers yields a striking difference in their MS^3^ fragment spectra: the formation of ketene and ketene–CO fragments is suppressed in the 6*Z* isomer (Fig. 4c). As the fragment intensities are expected to be determined by the geometry of the intermediate dioxolane fragment, IR spectra of [PC(18:1(9*Z*)/18:1(9*Z*)) + Ag − 183]^+^ and [PC(18:1(6*Z*)/18:1(6*Z*)) + Ag − 183]^+^ fragments (*m/z* 709) were measured (Fig. 4a). Interestingly, one of the two main absorption bands corresponding to the C = C stretching vibration of the C = C bond adjacent to the dioxolane ring is blue-shifted by 50 cm^−1^ in the spectrum of the 6*Z* isomer compared with the 9*Z* isomer. Based on this significant spectral difference, fundamental conformational differences are expected between the double bond isomers. DFT calculations were thus performed on both isomers, and the computed harmonic IR spectra reproduce the experimentally observed shift of the C = C stretching vibration conclusively (Fig. S7). The computed low-energy conformers adopt clearly different conformations depending on the C = C bond position within the lipid chains (Fig. 4b). In the 9*Z* isomer, the overall conformation and spectral signature are very similar to the [PC(16:0/18:1(9*Z*)) + Ag − 183]^+^ fragment. The silver ion coordinates to the carbonyl oxygen, the C = C bond next to the dioxolane ring, and the C = C bond of the oleic acid chain at the *sn*-2 position. The C = C bond of the second oleic acid chain is not involved. On the contrary, the C = C bonds in the petroselinic acid substituents of the [PC(18:1(6*Z*)/18:1(6*Z*)) + Ag − 183]^+^ fragment are both interacting simultaneously with the silver ion due to their proximity to the glycerol backbone. The coordination of both lipid chains instead of one leads to an overall distortion of the chains in close proximity to the dioxolane core structure in comparison with the 9*Z* isomer (Fig. S8). The different conformations of silver-adducted dioxolane fragments depend on the proximity of C = C bonds to the glycerol backbone and are key to explain the different CID spectra of 6*Z* and 9*Z* double bond isomers. The MS^3^ spectrum of the 9Z isomer displays the expected formation of both ketene and acid fragments because both oleic acid residues can be cleaved during fragmentation. In the 6*Z* isomer, however, ketene formation, which is associated with the loss of the fatty acyl at the *sn*-2 position, is hardly discernible. It can be assumed that the fatty acyl at the *sn*-1 position, which yields mainly acid fragments, is dissociated with high preference together with the silver cation. To provide an explanation, it is important to note that the fragments are only visible in the CID spectrum if they are associated with a positively charged silver ion. In the 6*Z* isomer, the fatty acyl at the *sn*-1 position coordinates strongly to the silver ion via its carbonyl oxygen and the C = C bond, whereas in the case of the 9*Z* isomer, the C = C bond of the fatty acyl at the *sn*-1 position does not participate in the silver coordination. Therefore, the silver ion is strongly localized at the *sn*-1 fatty acyl in the 6*Z* isomer, which leads to a high ratio of acid fragments, whereas both fatty acyls can be lost as silver adducts from the 9*Z* isomer. This example shows that cation-pi interactions strongly influence the fragmentation behavior of silver-adducted lipids.

### Allylic dioxolane fragments

**Fig. 5:**
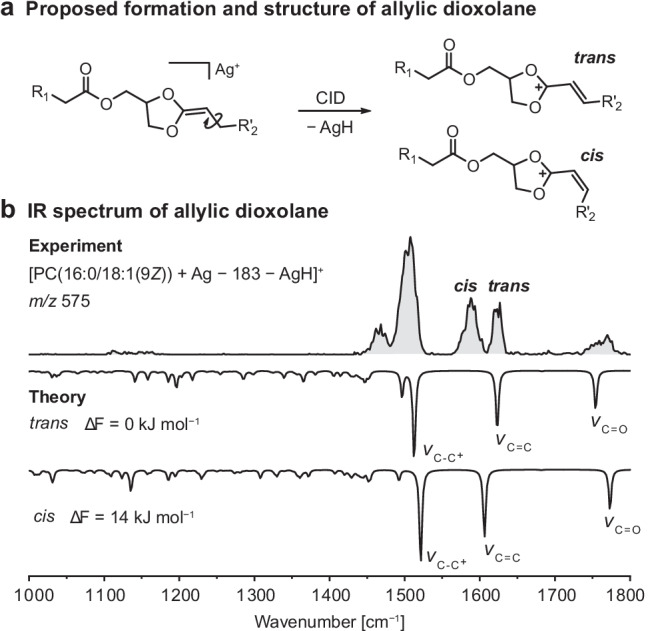
Proposed formation and IR signature of allylic dioxolane fragments. **a** Fragmentation of silver-adducted dioxolane fragments generated from PC(16:0/18:1(9*Z*)) results in neutral loss of silver hydride. The most stable fragment structure found by computation features an allylic cation. The residue R_2_ is replaced by R_2_’ = R_2_ − CH_2_ in the fragmentation scheme to illustrate the geometry of the allylic double bond. **b** Both *trans* and *cis* isomers contribute to the experimental IR spectrum of the allylic cation. Spectra were computed at the PBE0 + D3/6–311 + G(d,p) level of theory and are shown as inverted traces below the experimental spectra accompanied by their relative free energies (ΔF) at 90 K in kJ mol^−1^

Another particularity in the CID spectra of silver-adducted dioxolane fragments is the observation of abundant metal hydride loss, which does not occur in the case of alkali metal adduction (Fig. S14). In order to propose a fragmentation mechanism for hydride abstraction from dioxolane fragments, silver-adducted PC(16:0/18:1(9*Z*)) was subjected to neutral loss of phosphocholine and subsequent dissociation of silver hydride yielding [PC(16:0/18:1(9*Z*)) + Ag − 183 − AgH]^+^ fragments (*m/z* 575). The mass peak of the fragment ion does not show the characteristic silver isotope pattern, providing clear evidence that the silver ion is lost upon fragmentation. Even though in-source fragmentation does not allow for consecutive rounds of isolation and fragmentation required for MS^3^, the fragment of interest was generated by applying steeper voltage differences than required for the formation of MS^2^ fragments (Fig. S2). The IR spectrum of the obtained fragment is shown in Fig. 5b. It features a strong absorption at 1500 cm^−1^, two absorption bands of equal intensity between 1550 and 1650 cm^−1^, and a broad band between 1750 and 1800 cm^−1^, which is attributed to C = O stretching vibrations. The main absorption band at 1500 cm^−1^ coincides perfectly with the main absorption band of protonated dioxolane fragments [PC + H − 183]^+^, which stabilize the positive charge between the two ring oxygens [13]. The two absorption bands between 1550 and 1650 cm^−1^ are not present in the IR spectra of protonated fragments but highly diagnostic for the structural assignment of the fragment investigated here. Their position can only be explained by assuming an allylic dioxolane fragment, which is formed by hydride abstraction from the carbon atom next to the C = C bond (Fig. 5a). Before the hydride abstraction, the C–C bond next to the C = C bond can freely rotate but is locked in a *cis* or *trans* configuration upon dissociation of silver hydride. The coexistence of *cis* and *trans* isomers explains the observation of two separate bands corresponding to the C = C stretching vibration of the allylic cation. Rotation of the C–C^+^ bond next to the dioxolane ring gives rise to two possible conformers (Fig. S10). The match between experiment and theory supports the allylic structure (Fig. S9), which is also energetically favorable because of the mesomeric effect: the positive charge is well-stabilized by delocalization of electrons between the two ring oxygens and the allylic double bond. Computation of transition states for dioxolane model structures yielded an activation barrier for silver hydride abstraction of 150 kJ mol^−1^ (ΔG_298_) (Fig. S11). In comparison to this value, the energy difference between the formation of *cis* and *trans* allylic fragments is small, and hence both isomers are observed in comparable quantities. Interconversion between *cis* and *trans* isomers under the activating conditions in the source region is conceivable as well but rather unlikely because the computed activation barrier exceeds 200 kJ mol^−1^ (ΔG_298_) (Fig. S12). This significant amount of energy would have to be transferred to the allylic cation in addition to the activation energy required for its initial formation (Fig. S13). Therefore, the geometry of the double bond is assumed to be finally determined in the hydride abstraction reaction.

**Fig. 6:**
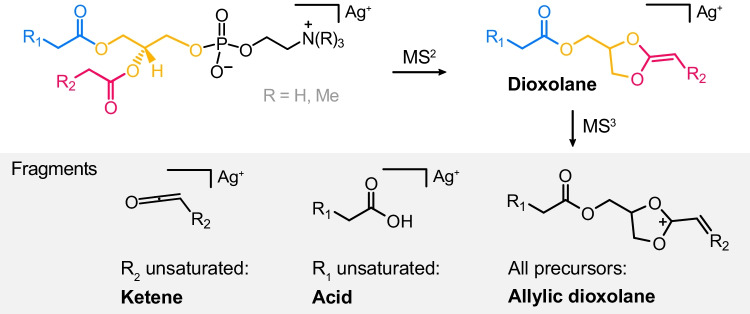
Overview of the fragmentation of phospholipid silver adducts. Silver-adducted phosphatidylethanolamine and phosphatidylcholine yield dioxolane silver adducts upon neutral headgroup loss. The latter dissociates into allylic dioxolane fragments as well as silver-adducted ketene and acid fragments. The relative abundance of ketene and acid fragments depends on the position of the unsaturated fatty acid and the double bond position

An overview scheme summarizing the fragmentation of phospholipid silver adducts studied in this work is shown in Fig. 6.

## Discussion

In the present study, the influence of silver adduction on the fragmentation behavior of glycerophospholipids was investigated and rationalized by a combination of cryogenic IR spectroscopy and computational chemistry. The approach was employed to determine the structure and coordination geometry of silver-adducted fragments, which, in turn, makes it possible to draw rational conclusions on the fragmentation mechanisms and relative fragment abundances. Neutral headgroup loss from silver-adducted PC and PE was found to yield dioxolane structures featuring specific coordination geometries depending on the lipid chain positions and position of C = C bonds within the lipid chains. Therefore, *sn*-isomers and double bond positional isomers yield distinguishable CID spectra, which can be qualitatively explained based on the geometry of the dioxolane fragments. Furthermore, allylic dioxolane fragment structures resulting from silver hydride abstraction were proposed and confirmed for the first time. Because the fragmentation behaviors differ little between different glycerolipids [8] and because fragment structures are assumed to be identical after neutral headgroup loss, the findings obtained for PC and PE are likely to be valid for other glycerophospholipid classes as well. Overall, gas-phase IR spectroscopy expands our understanding of lipid fragmentation mechanisms and reveals the influence of metal adduction on the fragmentation behavior of isomers.

Another potential field of application for cryogenic IR spectroscopy is the identification and quantification of phospholipid isomers based on spectral differences induced by subtle structural variations such as double bond and acyl chain position. Deconvolution of IR spectra of isomeric mixtures was demonstrated to be feasible using reference spectra of standards [32, 33]. This approach might be useful for specific applications such as distinguishing 6*Z* and 9*Z* double bond isomers, which feature sufficiently distinct IR spectra. For the *sn*-isomers investigated here, the subtle spectral differences and low absolute absorption intensities as well as the existence of isomeric impurities in commercial reference standards render a reliable mixture analysis impracticable. Furthermore, biological applications are rather unrealistic due to the large number of coexisting isomers and the need for reference standards to identify individual molecular structures and perform a spectral deconvolution. Unknown phospholipid isomers cannot be identified, and we observe no logical trend in the IR spectra yielding information about double bond or acyl chain position, which renders a systematic approach for isomer distinction very challenging. To gain a fundamental understanding of fragmentation processes in tandem mass spectrometry, however, cryogenic IR spectroscopy is a very powerful technique. A better identification of double bond and *sn*-isomers might be achieved in the future, e.g., by enhancing diagnostic spectral differences by chemical modification [34].

## Supplementary Information

Below is the link to the electronic supplementary material.

Supplementary file1 (PDF 2102 KB)

## Data Availability

All data generated or analyzed during this study are included in this published article and its supplementary information files.
