# Supplementary material for: Cryogenic infrared spectroscopy provides mechanistic insight into the fragmentation of phospholipid silver adducts
Source: Anal Bioanal Chem. 2022 Feb 11;414(18):5275–85. doi: 10.1007/s00216-022-03927-6 (PMC9242943; doi:10.1007/s00216-022-03927-6)
Supplement: Supplementary file 1 — Supplementary file1 (PDF 2102 KB) [file 216_2022_3927_MOESM1_ESM.pdf]

Cryogenic infrared spectroscopy provides mechanistic insight into the fragmentation of phospholipid silver adducts

Supplementary Information

Carla Kirschbaum,[a,b†] Kim Greis,[a,b†] Sandy Gewinner,[b] Wieland Schöllkopf,[b] Gerard Meijer,[b] Gert von Helden,[b] and Kevin Pagel[a,b]

a Institut für Chemie und Biochemie, Freie Universität Berlin, 14195 Berlin, Germany

b Fritz-Haber-Institut der Max-Planck-Gesellschaft, 14195 Berlin, Germany

† Authors contributed equally

Correspondence to: kevin.pagel@fu-berlin.de

Table of Contents

In-source fragmentation **3**
Figure S1. 3
Figure S2. 4
Silver coordination sites in model lipids **5**
Figure S3. 6
Computed infrared spectra **7**
Figure S4. 7
Figure S5. 8
Figure S6. 8
Figure S7. 9
Figure S8. 10
Allylic dioxolane fragments **11**
Figure S9. 11
Figure S10. 12
Figure S11. 12
Figure S12. 13
Figure S13. 13
Tandem mass spectra **14**
Figure S14. 14
XYZ coordinates of computed conformers **15**
[PE(3:0/3:0) + Ag − 141]$^+$ 15
[PE(6:0/6:0) + Ag − 141]$^+$ 16
[PE(16:0/18:1) + Ag − 141]$^+$ 19
[PE(18:1/16:0) + Ag − 141]$^+$ 21
[PC(18:1(9*Z*)/18:1(9*Z*)) + Ag − 183]$^+$ 24
[PC(18:1(6*Z*)/18:1(6*Z*)) + Ag − 183]$^+$ 28
[PC(16:0/18:1) + Ag − 183 − AgH]$^+$ 31
[PC(3:0/4:0) + Ag − 183 − AgH]$^+$ 36
PC(3:0/4:0) Hydride abstraction 37
PC(3:0/4:0) *cis*/*trans* isomerization 39
References **41**

In-source fragmentation

In-source fragmentation is equivalent to collision-induced dissociation (CID) based on the acceleration of molecular ions and subsequent collisions with a buffer gas. In the present setup, the ions are accelerated after nano-electrospray ionization and collide with residual gas molecules present in the differentially pumped source region. In order to achieve sufficiently high acceleration, the voltages on the source block and the two ring electrode ion guides (IG) are tuned manually for each ion. Typically, the source block and offset of the first ion guide are set to a maximum value of 150 V. The ions are thus accelerated starting from a high potential towards the second ion guide and then decelerated by an elevated potential on the endcap of the second ion guide. Both the high offset voltage on the source block and first ion guide, and the higher potential on the endcap of the second ion guide compared to the IG 2 offset, are required for fragmentation. Typical voltages applied to each part in the source region are shown below.

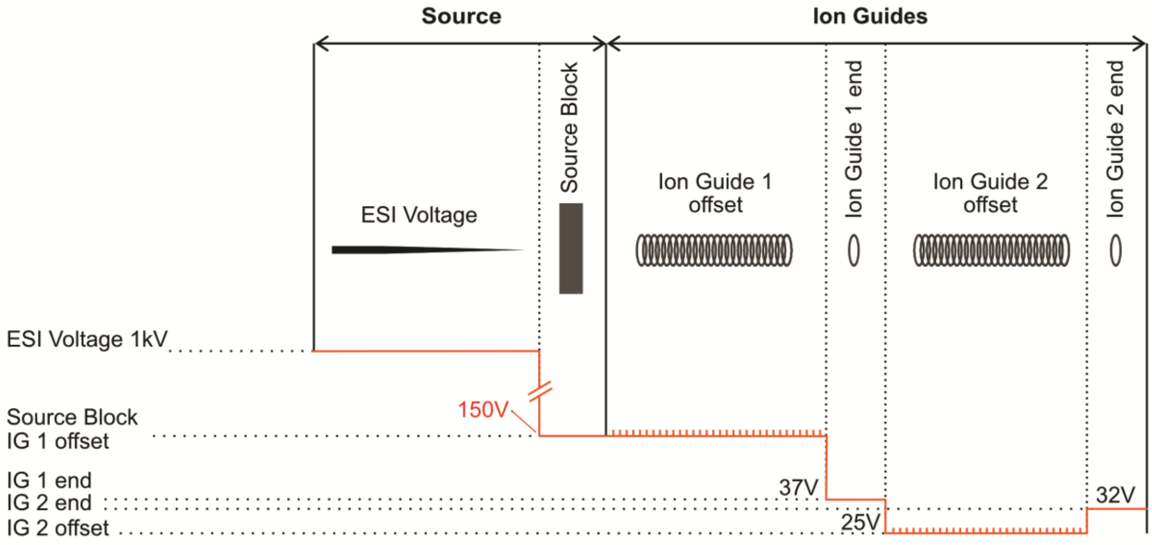

Figure S1. Voltage scheme of the source region to induce in-source fragmentation. Precursor ions are generated by nano-electrospray ionization and accelerated from the source block towards the second ion guide. Typical values employed for the fragmentation of silver-adducted phospholipids are shown in the scheme. The drawing is not to scale.

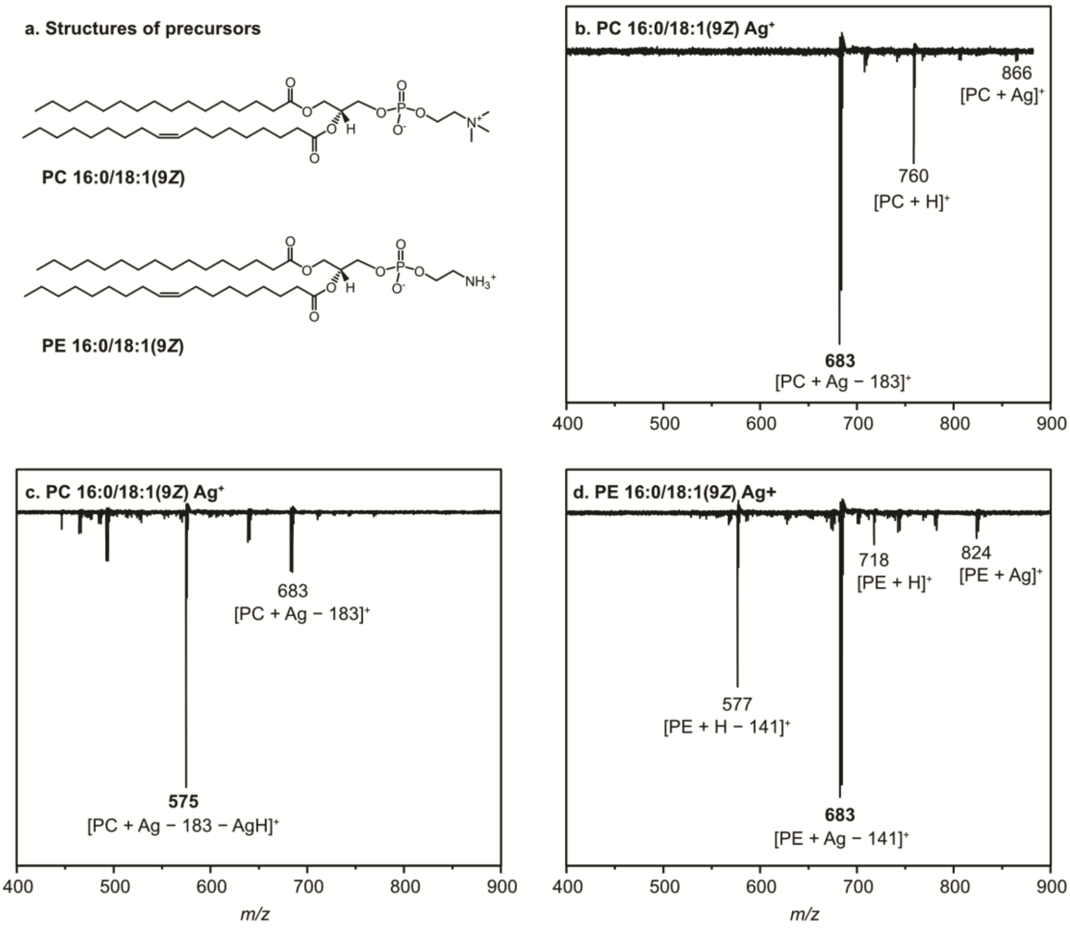

Figure S2. Mass spectra showing in-source fragmentation of silver-adducted phospholipids. a) Chemical structure formulae of the precursors PC(16:0/18:1(9*Z*)) and PE(16:0/18:1(9*Z*)). b) Silver adducts of PC(16:0/18:1(9*Z*)) readily eliminate phosphocholine (183 u). c) By applying steeper voltage differences than in (b), PC(16:0/18:1(9*Z*)) yields an MS^3 fragment at *m/z* 575 resulting from the loss of phosphocholine and silver hydride. d) Silver adducts of PE(16:0/18:1(9*Z*)) undergo neutral loss of phosphoethanolamine (141 u).

Silver coordination sites in model lipids

Coordination sites of Ag^+ on glycerophospholipid fragments were identified using the coordination site screening implemented in CREST[1] (keywords *-protonate -swel Ag+*) employing the semi-empirical method GFN2-xTB[2] and default settings. The search was performed on a model lipid truncated to three carbon atoms per acyl residue for both dioxolane and dioxane structures. Several coordination geometries were initially found and subjected to a conformational search in CREST. Selected conformers were then optimized in Gaussian 16[3] at the PBE0+D3/6-311+G(d,p)[4-5] level of theory including an SDD effective core potential for silver. Harmonic vibrational spectra were calculated at the same level of theory and scaled by a factor of 0.965. Free energies including zero-point correction were calculated at 90 K.

The coordination site screening yielded one energetically most favored geometry for both dioxolane and dioxane structures, in which the silver ion coordinates to the carbonyl oxygen and to the sp^2 carbon of the C=C double bond next to the ring. Coordination of the ring oxygen is energetically less favored and decreases the match with the experimental spectra significantly. Overall, the computed spectra of the model lipid yield an unsatisfactory match with the experimental spectrum of $[PE(16:0/18:1(9Z)) + Ag - 141]^+$, which suggests a major influence of the acyl chain length on the band positions in the IR spectrum.

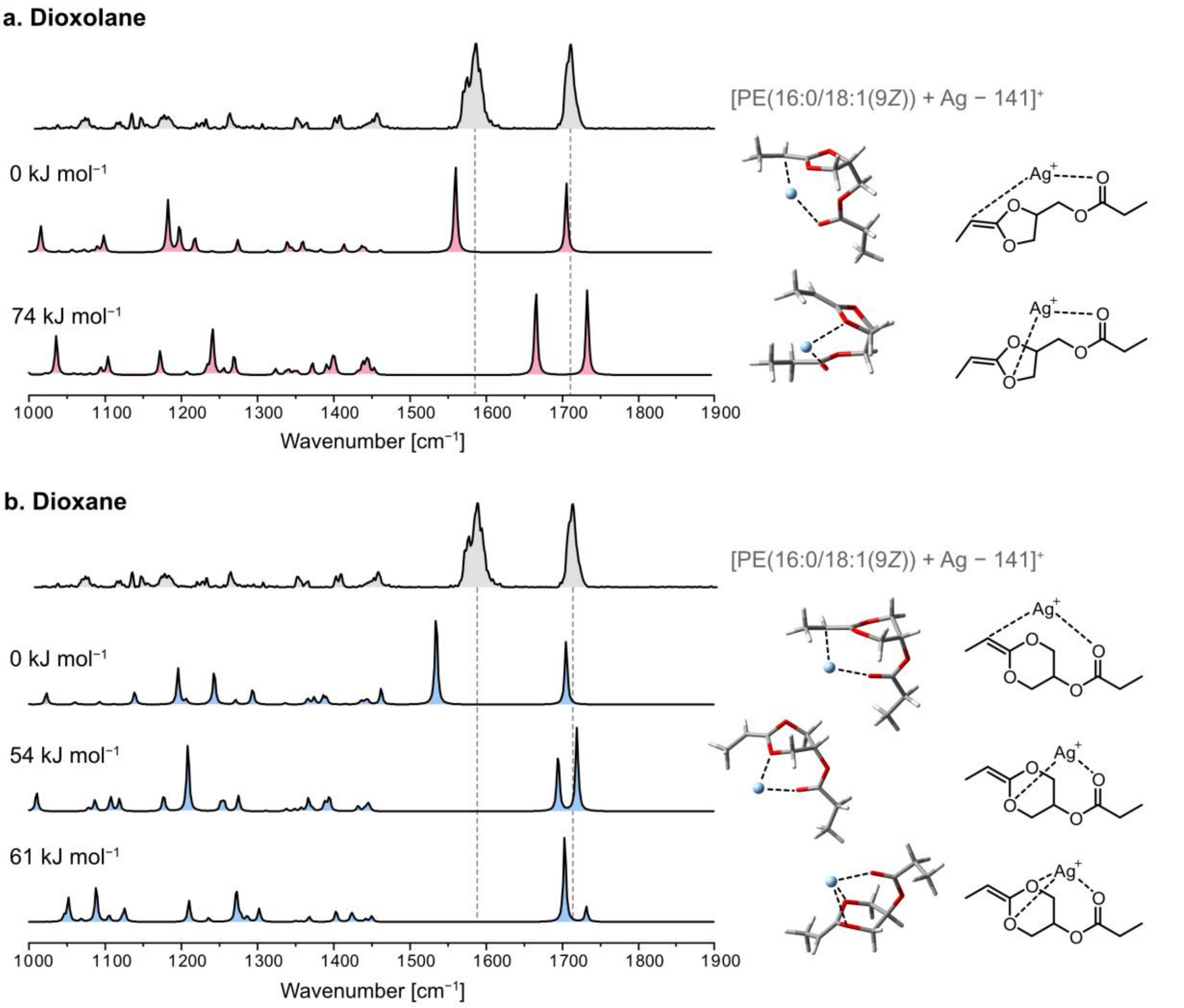

Figure S3. Computed IR spectra of a) dioxolane and b) dioxane model structures truncated to three carbons per fatty acid (3:0/3:0) with different silver coordination sites. The experimental spectrum of [PE(16:0/18:1(9*Z*)) + Ag − 141]$^+$ (gray) is shown above the computed spectra for comparison. In the most stable conformers, the silver ion coordinates the carbonyl oxygen and the C=C double bond adjacent to the ring. Spectra were computed at the PBE0+D3/6-311+G(d,p), SDD (Ag) level of theory, and relative free energies at 90 K refer to the most stable conformer of each structure motif. XYZ coordinates of all computed structures are listed on page 15ff.

Computed infrared spectra

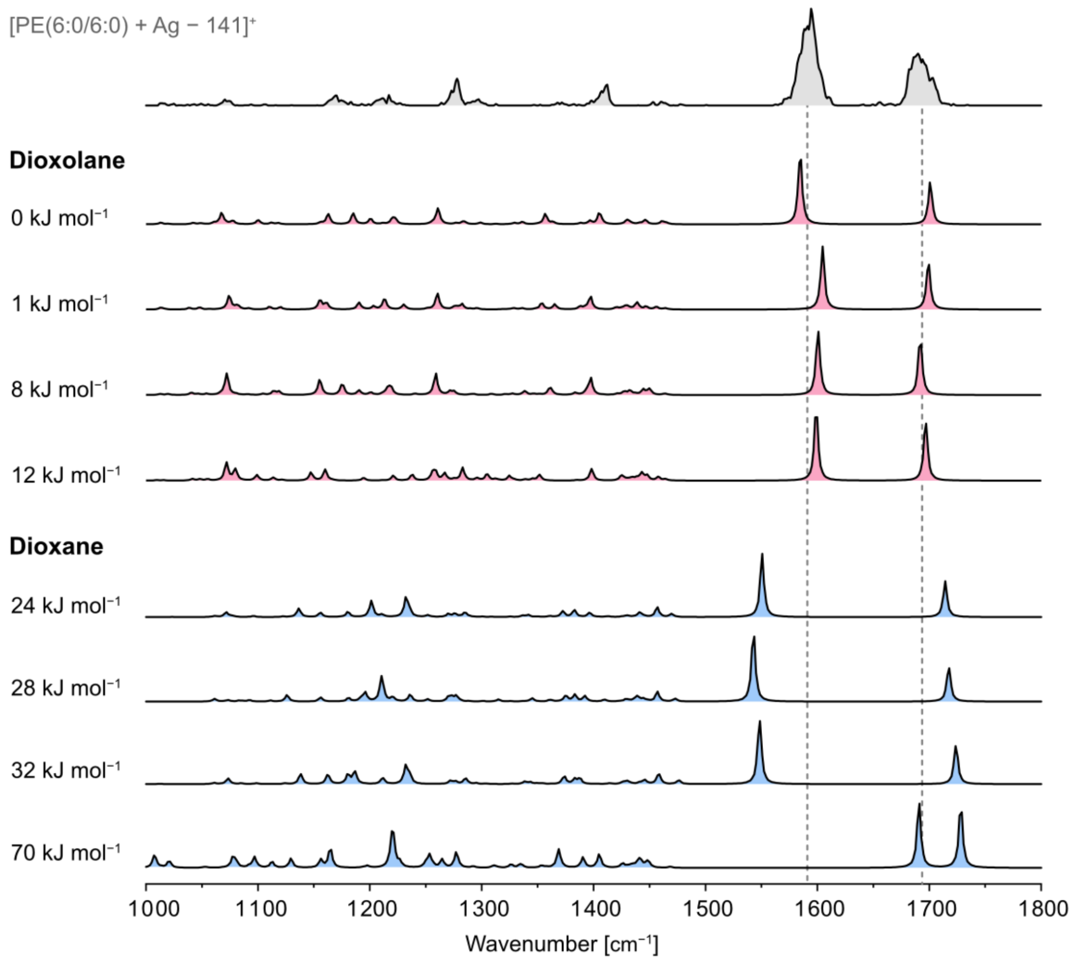

Figure S4. Computed IR spectra and relative free energies at 90 K of silver-adducted dioxolane and dioxane fragments equipped with hexanoic acid chains (6:0/6:0). The experimental spectrum of $[PE(6:0/6:0) + Ag - 141]^+$ (gray) is well modeled by the computed spectra of dioxolane structures. Dioxane structures are energetically disfavored, and their IR signatures do not match the experimental spectrum. The deviating band positions in the computed dioxane spectrum at the bottom is caused by energetically unfavorable interactions between the silver ion and ring oxygen. Spectra were computed at the PBE0+D3/6-311+G(d,p), SDD (Ag) level of theory. XYZ coordinates of all computed structures are listed on page 16ff.

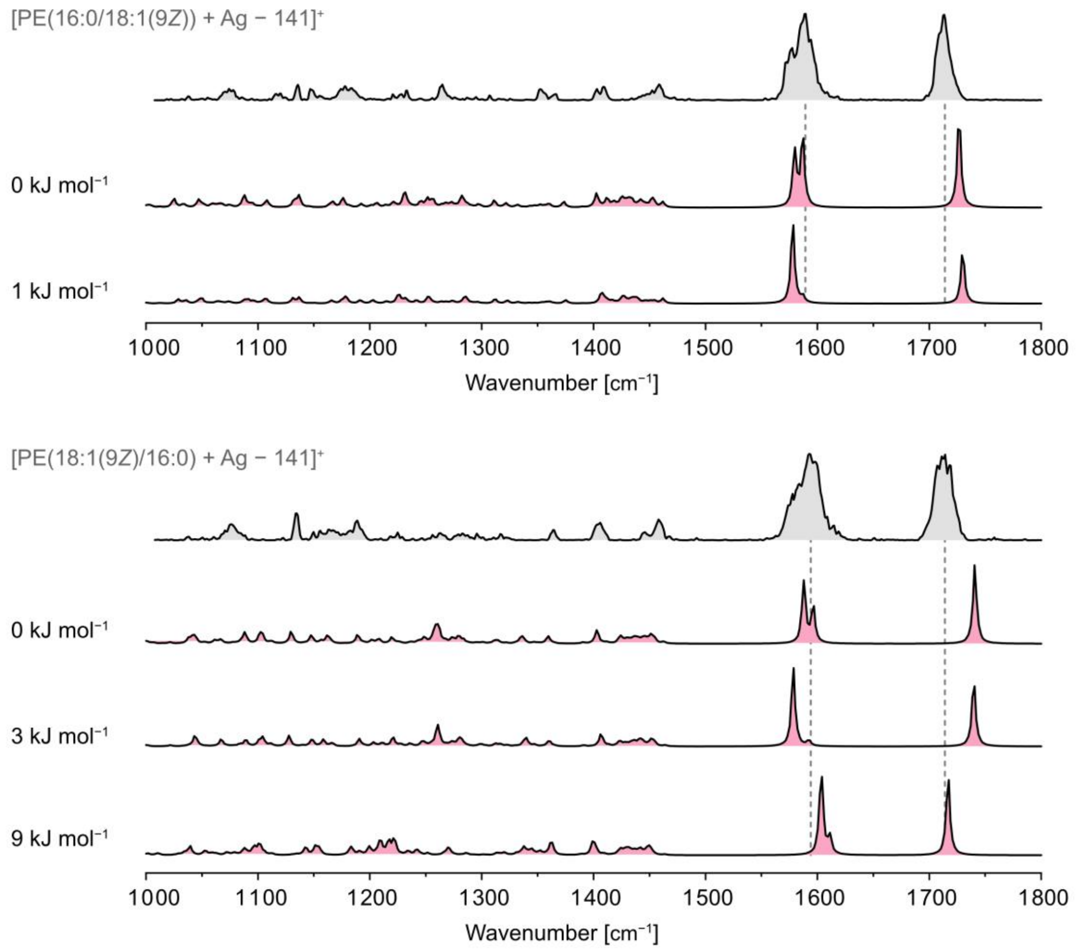

Figure S5. Computed IR spectra and relative free energies at 90 K of silver-adducted dioxolane structures derived from PE(16:0/18:1(9*Z*)) and PE(18:1(9*Z*)/16:0). Spectra were computed at the PBE0+D3/6-311+G(d,p), SDD (Ag) level of theory and XYZ coordinates of all structures are listed on page 19ff.

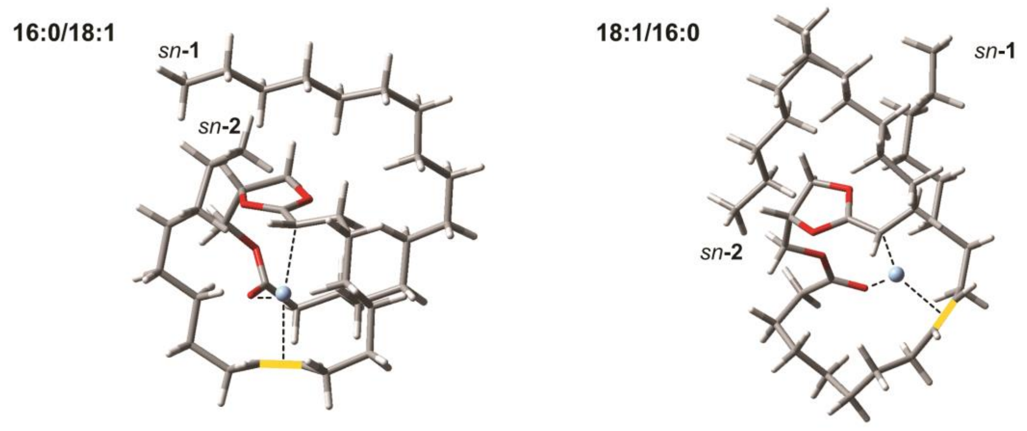

Figure S6. Structures of the lowest-energy computed dioxolane conformers of [PE + Ag − 141]$^+$ fragments derived from PE(16:0/18:1(9*Z*)) and PE(18:1(9*Z*)/16:0). The silver cation coordinates to the carbonyl oxygen and to the two C=C bonds. The C=C bond in the oleic acid residue is highlighted in yellow.

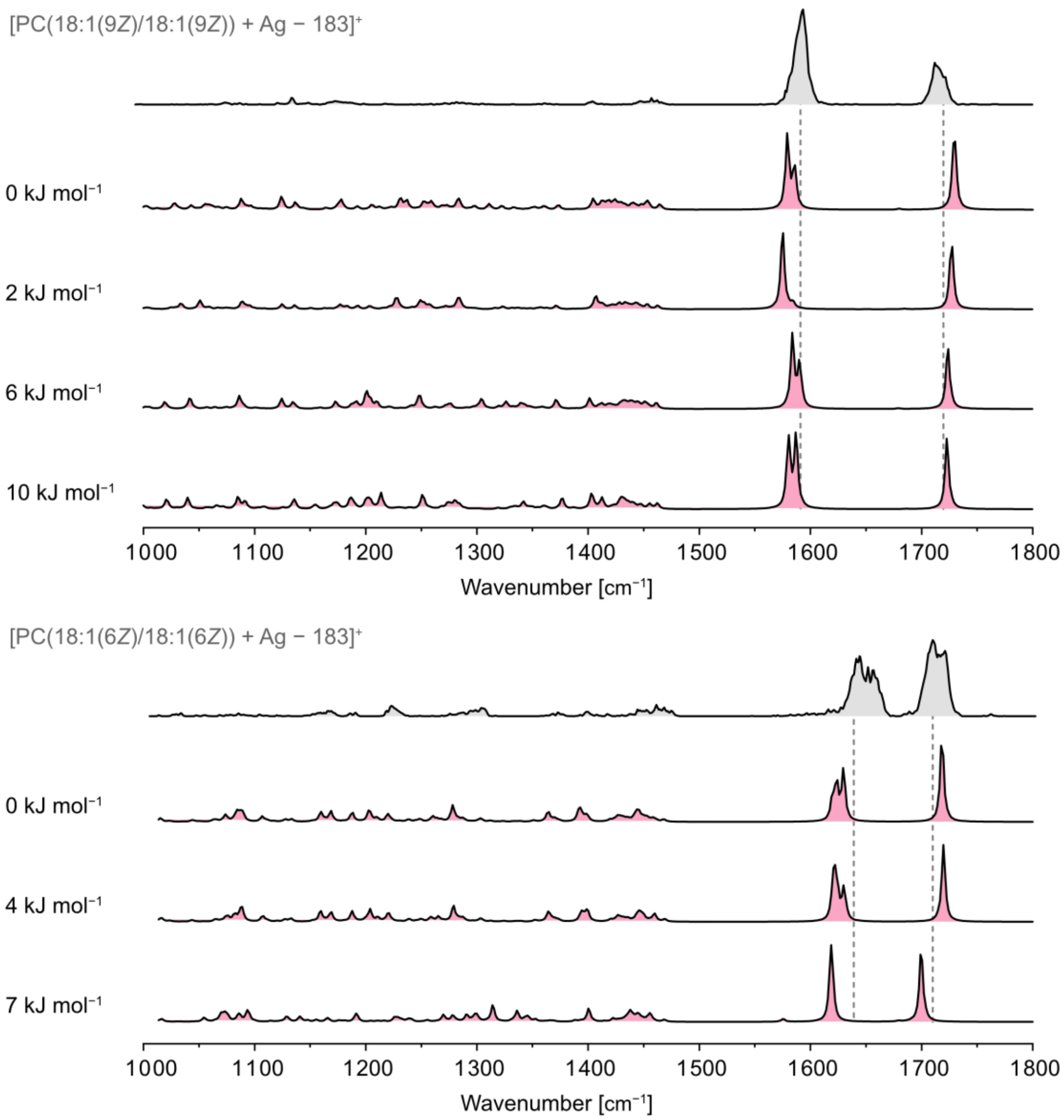

Figure S7. Computed IR spectra and relative free energies at 90 K of silver-adducted dioxolane structures derived from PC(18:1/18:1) double bond isomers (9*Z* and 6*Z*). The computed spectra model the band shifts observed in the experimental IR spectra (gray), which are dependent on the double bond position, reasonably well. Spectra were computed at the PBE0+D3/6-311+G(d,p), SDD (Ag) level of theory and XYZ coordinates of all computed structures are listed on page 24ff.

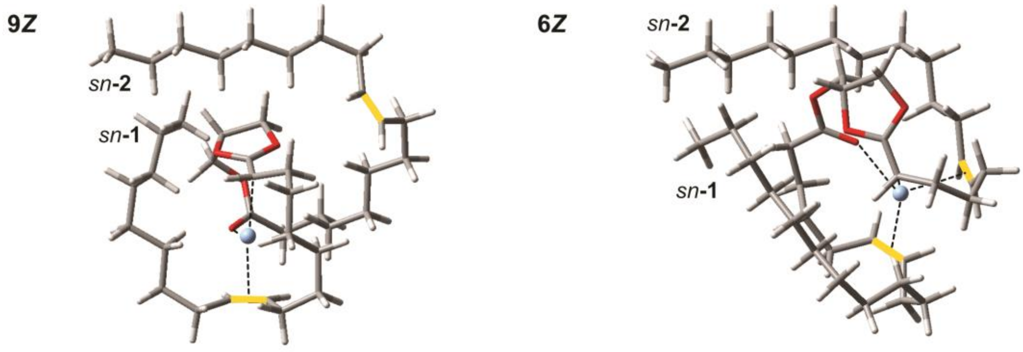

Figure S8. Structures of the lowest-energy computed dioxolane conformers of $[\mathrm{PC} + \mathrm{Ag} - 183]^+$ fragments derived from PC(18:1(9*Z*)/18:1(9*Z*)) and PC(18:1(6*Z*)/18:1(6*Z*)). The silver cation coordinates to the carbonyl oxygen, the C=C bond adjacent to the ring and to the C=C bonds of one (9*Z*) or two (6*Z*) acyl residues. The C=C bonds in the fatty acyl chains are highlighted in yellow.

Allylic dioxolane fragments

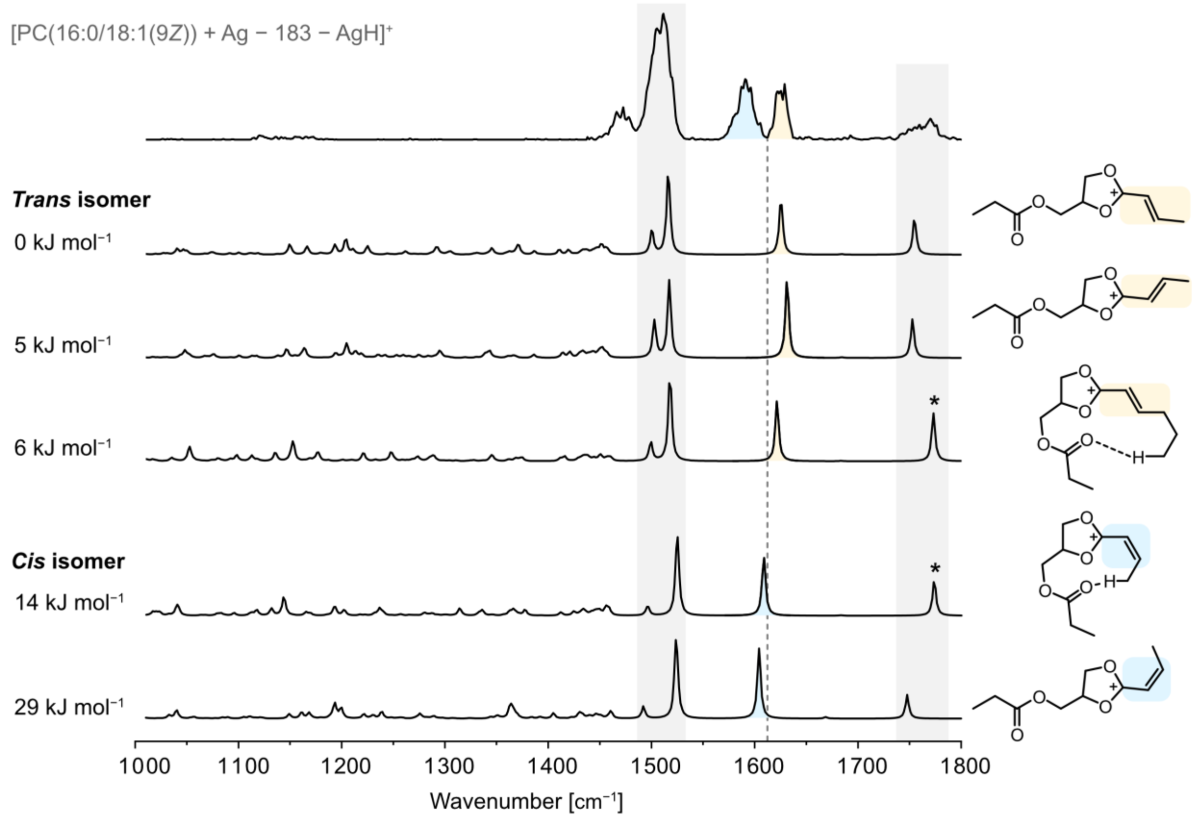

Figure S9. Computed IR spectra and relative free energies at 90 K of allylic dioxolane fragments generated from silver-adducted PC(16:0/18:1(9*Z*)). The core structures of computed conformers are depicted with truncated lipid chains for visibility. The allylic cation features two diagnostic bands between 1550–1650 cm^{-1}, which were attributed to *cis* and *trans* isomers. The carbonyl stretching frequency is shifted by interactions between the carbonyl oxygen and hydrogen atoms of the lipid chain (marked by asterisks). Spectra were computed at the PBE0+D3/6-311+G(d,p) level of theory and XYZ coordinates of all structures are listed on page 31ff.

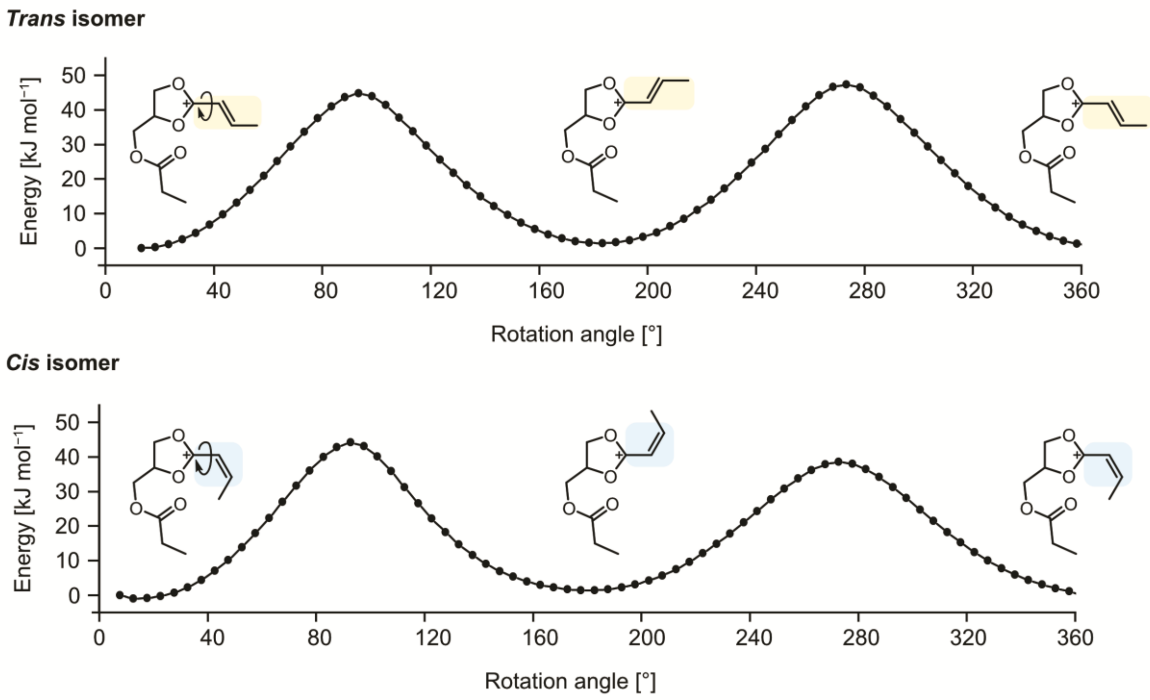

Figure S10. Rotation barriers for *trans* and *cis* isomers of truncated allylic dioxolane fragments (3:0/4:0). Single point energies were computed at the PBE0+D3/6-311+G(d,p) level of theory in steps of 5° in a potential energy surface scan. XYZ coordinates of the initial structures are given on page 36f.

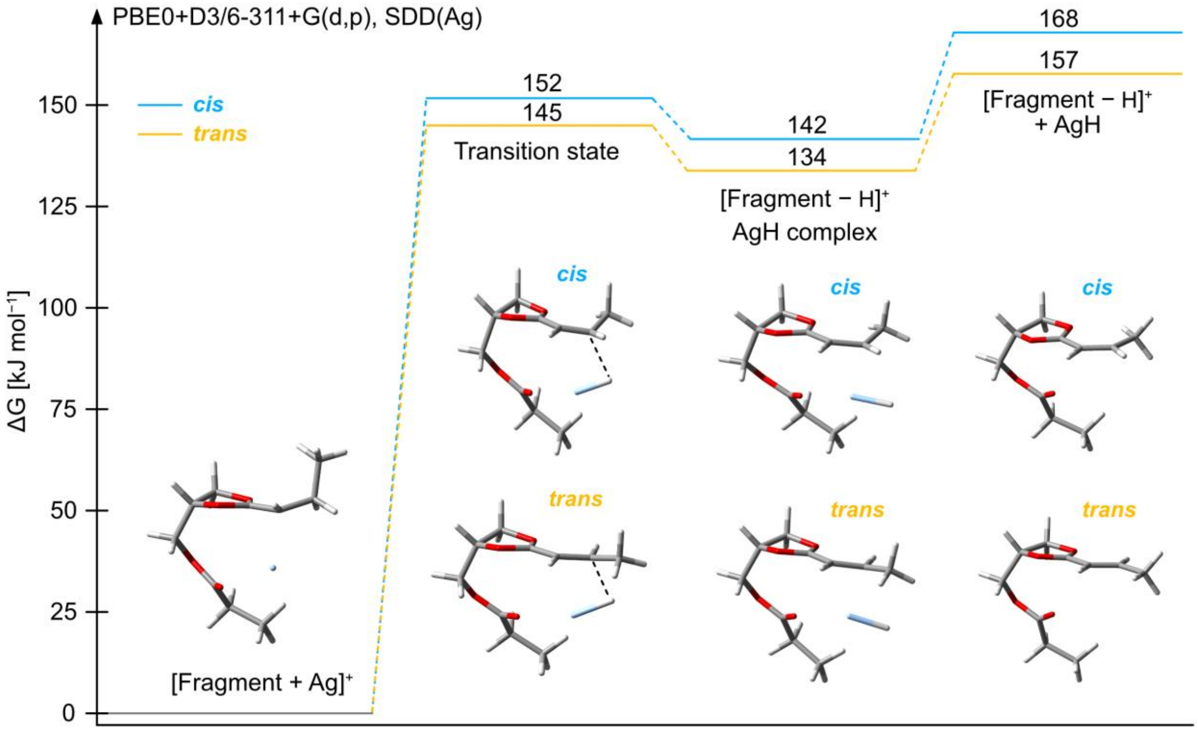

Figure S11. Computed transition states for the formation of *cis* and *trans* allylic dioxolane fragments from a silver-adducted dioxolane model structure (3:0/4:0). Gibbs energies were computed at the PBE0+D3/6-311+G(d,p), SDD (Ag) level of theory at 298.15 K. The activation barriers leading to *cis* and *trans* products are energetically similar and within the range of reactions possible in the source region. Energetics and structures are shown for the allylic fragment-AgH-complex as well as the allylic fragment separated from AgH. XYZ coordinates of all structures are given on page 37f.

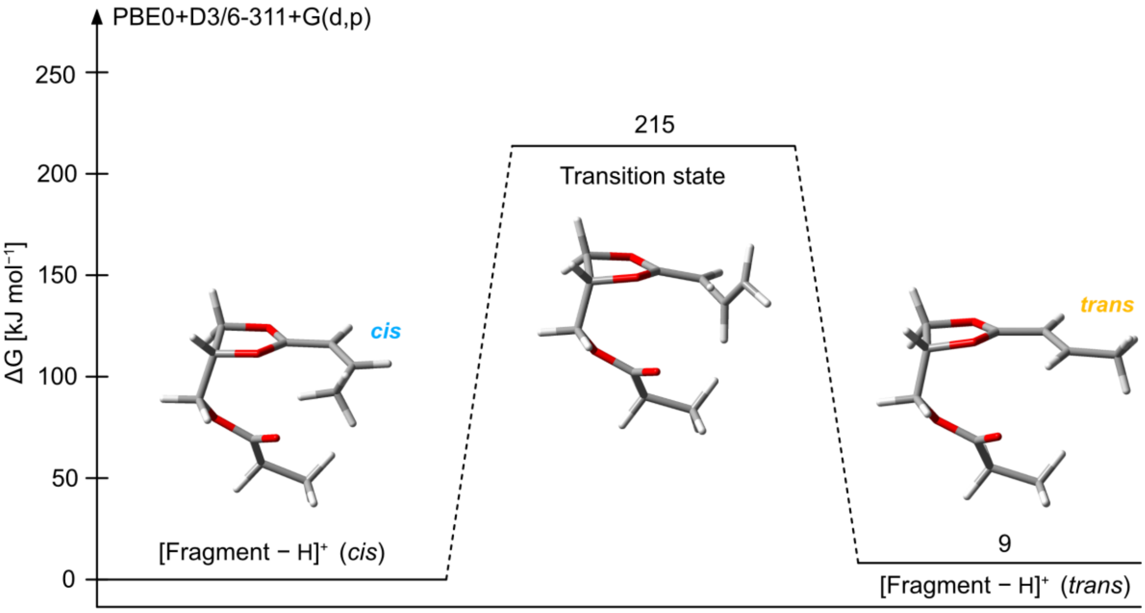

Figure S12. Computed transition state for *cis/trans* isomerization of the allylic dioxolane model structure (3:0/4:0). Gibbs energies were computed at the PBE0+D3/6-311+G(d,p) level of theory at 298.15 K. XYZ coordinates of all structures are given on page 39f.

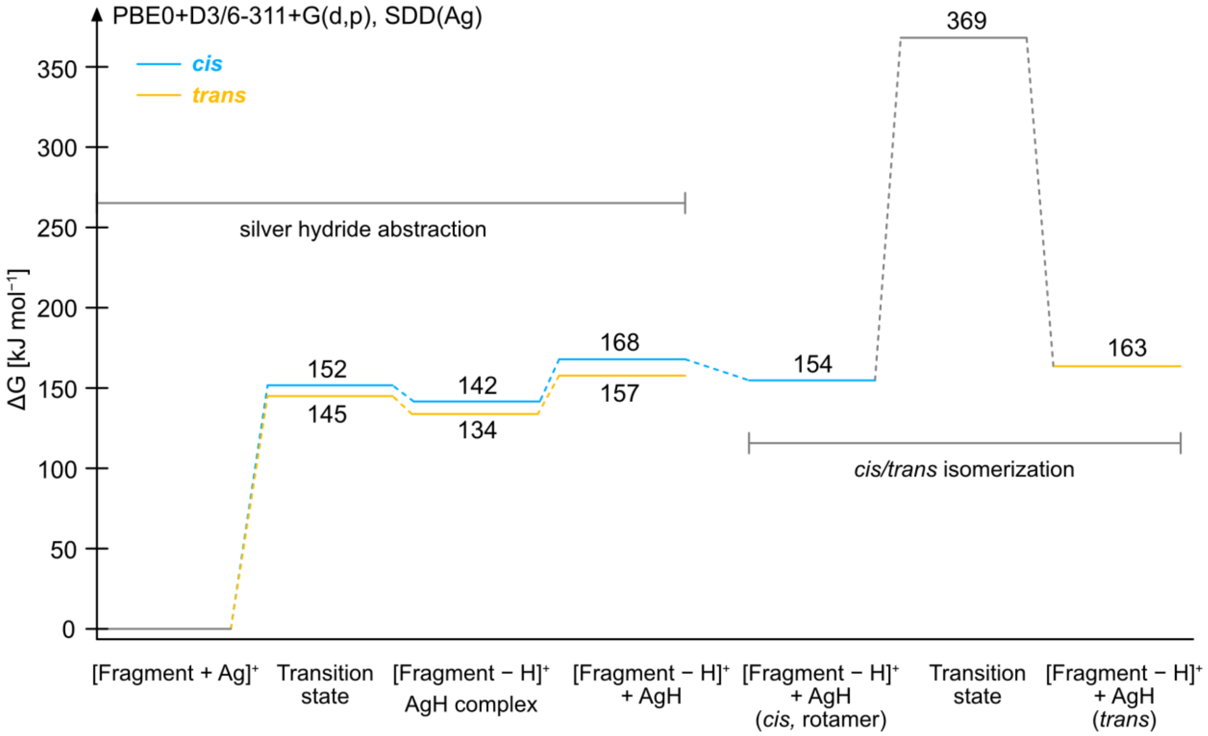

Figure S13. Overview scheme combining the computed energetics for the abstraction of silver hydride from silver-adducted dioxolane model fragments (Figure S11) and *cis/trans* isomerization of the resulting allylic dioxolane fragment (Figure S12). Comparison of the activation barriers demonstrates that *cis/trans* isomerization of the allylic dioxolane fragments after their formation is highly improbable due to the considerable activation energy for rotation around the C=C bond.

Tandem mass spectra

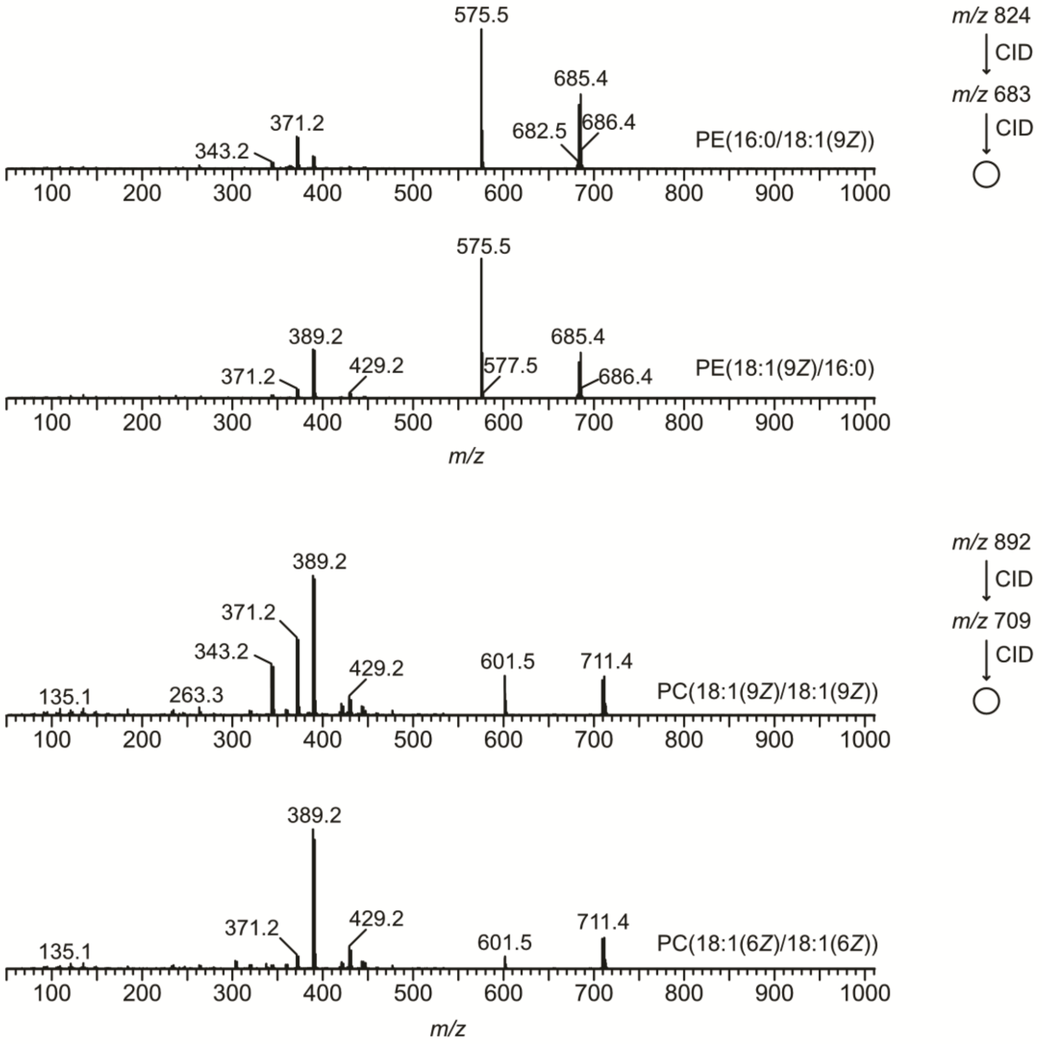

Figure S14. Tandem MS spectra of silver-adducted dioxolane *sn*-isomers (*m/z* 683) and dioxolane double bond isomers (*m/z* 709). Silver-adducted dioxolanes eliminate silver hydride yielding allylic dioxolane fragments at *m/z* 575 and 601, respectively. The fragment spectra of *sn*- and double bond isomers exhibit different ratios of oleic acid in the form of ketene (*m/z* 371) and carboxylic acid (*m/z* 389). Ketene formation is suppressed in the 6*Z* isomer of PC(18:1/18:1).

XYZ coordinates of computed conformers

In the following, XYZ coordinates of all conformers corresponding to the computed IR spectra shown in this work are listed. The structures were optimized at the PBE0+D3/6-311+G(d,p) level of theory with an SDD effective core potential for silver. Each conformer is uniquely identifiable by its name and relative free energy (90 K). The name "conf_x" refers to the position x of the conformer in the CREST output file from the conformer sampling. The charge of all conformers is +1 and the multiplicity is 1.

[PE(3:0/3:0) + Ag − 141]$^+$

Figure S3

DIOXOLANE

conf_8 0.0 kJ mol^{-1}

```
C 3.497735 0.516231 0.011508
C 2.117985 -0.026850 0.165517
O 1.843966 -1.205703 0.282817
Ag -0.424418 -1.455216 -0.038025
C -2.379735 -0.460960 -0.558690
C -1.802493 0.760790 -0.263380
O -1.888770 1.343600 0.919192
C -1.116135 2.551658 0.915791
H -0.439224 2.523954 1.770127
H -1.801740 3.394461 1.008589
C -0.397449 2.515881 -0.436358
H -0.506252 3.447528 -0.993228
C 1.067526 2.126674 -0.377776
H 1.483879 2.087948 -1.387662
H 1.632490 2.843792 0.226913
O 1.078721 0.825214 0.208252
O -1.123573 1.495920 -1.144871
C -3.500017 -0.997564 0.304117
H -4.448030 -0.515183 0.043693
H -3.628282 -2.070133 0.155344
H -3.317738 -0.821093 1.365526
H -2.417500 -0.664526 -1.627330
H 3.532753 1.059852 -0.942280
H 3.637711 1.280653 0.786540
C 4.575864 -0.550642 0.072049
H 5.557743 -0.091270 -0.050565
H 4.557749 -1.073211 1.029663
H 4.441651 -1.291174 -0.717940
```

conf_13 74.4 kJ mol^{-1}

```
C -0.167182 2.575953 0.939398
C 0.638575 1.735009 0.000169
O 0.194391 1.163467 -0.990128
Ag -1.555083 -0.283855 -0.700284
C -0.038012 -1.314845 1.883067
C 0.849032 -1.286662 0.891495
O 0.535342 -1.572537 -0.413933
C 1.711564 -1.377789 -1.215148
H 1.440196 -0.812709 -2.105465
H 2.102827 -2.359748 -1.487381
C 2.662129 -0.629801 -0.281641
H 3.665376 -1.055615 -0.348707
C 2.808032 0.891988 -0.466408
H 3.786816 1.209345 -0.112162
H 2.701926 1.159879 -1.520670
O 1.907138 1.672181 0.331947
O 2.149343 -0.954099 1.007301
C -1.444149 -1.802668 1.733387
H -1.626665 -2.707183 2.324647
H -2.181135 -1.066663 2.076072
H -1.680964 -2.116929 0.699207
H 0.321521 -1.034114 2.865210
H 0.208047 2.407615 1.951514
H 0.073719 3.619306 0.699670
C -1.661982 2.339159 0.834146
H -1.920612 1.311589 1.138633
H -2.206346 2.995422 1.515477
H -2.033538 2.536852 -0.174981
```

DIOXANE

conf_0 0.0 kJ mol^{-1}

```
C -3.291144 -0.955892 0.005585
C -2.103906 -0.074750 -0.216688
O -1.007340 -0.450297 -0.587019
Ag 1.044481 -1.479570 0.095555
C 2.281228 0.397629 0.426058
C 1.146005 1.186864 0.287658
O 0.898093 1.802074 -0.846578
C -0.431459 2.204590 -1.237075
C -1.420255 2.265333 -0.082681
H -2.052242 3.145492 -0.210056
C -0.643133 2.408063 1.203438
H -1.283386 2.310460 2.077882
H -0.142811 3.382013 1.227566
O 0.331880 1.369089 1.321328
O -2.389781 1.206009 0.010715
H -0.755038 1.531597 -2.030187
H -0.295919 3.204424 -1.650200
C 3.375165 0.450906 -0.615580
H 3.971234 1.362456 -0.497035
H 4.054386 -0.396004 -0.513051
H 2.976842 0.449706 -1.631491
H 2.576949 0.257914 1.463947
C -2.970049 -2.435991 -0.079255
```

H	-2.544964	-2.696635	-1.050521
H	-2.263465	-2.735752	0.699795
H	-3.878946	-3.023460	0.059266
H	-3.732159	-0.682596	0.969546
H	-4.038622	-0.665333	-0.742881

conf_12 53.9 kJ mol^{-1}

C	3.681851	-0.005220	-0.192688
C	2.233610	0.329592	-0.031920
O	1.323286	-0.470514	-0.178126
Ag	-0.630281	-1.565587	0.079941
C	-2.861695	0.525508	-0.719134
C	-1.831560	1.203824	-0.221137
O	-1.113960	0.674803	0.829857
C	-0.178489	1.567609	1.439828
C	0.758628	2.218232	0.430255
H	1.026973	3.200874	0.819227
C	0.036307	2.355803	-0.914032
H	0.288305	1.523304	-1.579296
H	0.275761	3.293874	-1.410008
O	-1.373904	2.377143	-0.683498
O	2.053948	1.604572	0.281694
H	0.363672	0.986175	2.184487
H	-0.740827	2.351302	1.953638
C	-3.332896	-0.751929	-0.102818
H	-4.334278	-1.022255	-0.442562
H	-2.724116	-1.646306	-0.416716
H	-3.346363	-0.717180	0.989004
H	-3.345393	0.925540	-1.600205
C	3.947199	-1.479170	-0.430712
H	3.445130	-1.834210	-1.332842
H	5.018276	-1.647380	-0.553062
H	3.607302	-2.087015	0.410827
H	4.053833	0.611752	-1.019804
H	4.202252	0.370568	0.694838

conf_18 60.5 kJ mol^{-1}

C	3.758103	-0.117098	-0.614421
C	2.280899	-0.217108	-0.408729
O	1.552688	0.755644	-0.299173
Ag	-0.454146	1.672449	0.012880
C	-3.201084	-0.798805	0.576128
C	-1.908578	-0.646719	0.327596
O	-1.352577	-0.541095	-0.927315
C	-0.465259	-1.638085	-1.190189
C	0.529231	-1.922742	-0.063012
H	0.693780	-3.001234	-0.052814
C	-0.012400	-1.544158	1.316998
H	0.796569	-1.258039	1.989041
H	-0.528505	-2.412028	1.740245
O	-0.952719	-0.460365	1.303360
O	1.877858	-1.473789	-0.327384
H	0.054976	-1.413096	-2.120952
H	-1.086330	-2.526847	-1.341653
C	-4.260389	-0.924321	-0.461435
H	-3.847663	-0.896376	-1.470366
H	-4.809473	-1.861956	-0.332632
H	-4.992987	-0.117239	-0.362211
H	-3.483933	-0.807667	1.624530
C	4.511089	-0.393775	0.690605
H	5.584158	-0.294523	0.519892
H	4.229332	0.318523	1.469985
H	4.318511	-1.406409	1.050823
H	4.053997	-0.842704	-1.376358
H	3.970765	0.888187	-0.980034

[PE(6:0/6:0) + Ag − 141]$^{+}$

Figure S4

DIOXOLANE

conf_17 0.0 kJ mol^{-1}

C	2.724632	-2.155590	-0.548088
C	2.438497	-0.920994	0.247145
O	1.414342	-0.709713	0.870948
O	3.437793	-0.038239	0.217235
C	3.189429	1.210646	0.855948
C	2.684669	2.232162	-0.155994
O	1.627092	1.683416	-0.965282
C	0.454260	2.115356	-0.498229
C	-0.759310	1.688171	-0.980294
C	-2.019585	2.488709	-0.726606
C	-3.301877	1.657124	-0.662485
C	-3.541866	0.967813	0.673741
C	-4.723744	0.012912	0.657374
H	-4.579991	-0.785312	-0.078116
H	-4.880705	-0.450948	1.634103
H	-5.643768	0.540657	0.390107
H	-3.676290	1.727743	1.452043
H	-2.636307	0.408137	0.994655
H	-4.155079	2.313488	-0.862826
H	-3.300604	0.916657	-1.473745
H	-1.909600	3.065264	0.196947
H	-2.114891	3.222155	-1.536108
H	-0.671666	1.185245	-1.945048
O	0.601964	3.027416	0.453141
C	1.987613	3.410611	0.512565
H	2.111324	4.346344	-0.035718
H	2.255602	3.552211	1.559597
H	3.475718	2.527087	-0.844809
H	2.473014	1.074506	1.667877
H	4.144542	1.548177	1.261281
C	1.457506	-2.917872	-0.923192
C	0.822036	-3.685614	0.235664
C	-0.628548	-4.089731	-0.012156
C	-1.605108	-2.918155	-0.005507
H	-1.447863	-2.259767	-0.881180
H	-2.647023	-3.237921	-0.076783
H	-1.525732	-2.365721	0.950105
H	-0.936677	-4.795366	0.764999
H	-0.715152	-4.625304	-0.964331
H	1.417744	-4.584283	0.427549
H	0.867879	-3.087185	1.151694
H	0.745112	-2.204334	-1.359030
H	1.698391	-3.615462	-1.730864
H	3.396216	-2.780856	0.055153

H	3.302762	-1.860445	-1.427459
Ag	-0.78230	-0.341513	0.086856

conf_0 0.6 kJ mol^{-1}

C	3.456081	1.131103	0.205032
C	2.056334	1.444224	0.631317
O	1.287692	0.646535	1.137331
O	1.733861	2.722579	0.436919
C	0.411682	3.118520	0.788348
C	-0.465316	3.197266	-0.450641
O	-0.400570	1.974352	-1.205920
C	-1.457991	1.218944	-0.886915
C	-1.630774	-0.069602	-1.311629
C	-2.978082	-0.756724	-1.235947
C	-3.030199	-2.105891	-0.501234
C	-3.395597	-2.031425	0.978962
C	-2.408236	-1.294645	1.871918
H	-2.292783	-0.243793	1.586968
H	-2.727609	-1.306186	2.917526
H	-1.419684	-1.795093	1.870104
H	-3.524624	-3.050881	1.356763
H	-4.377030	-1.551201	1.070907
H	-3.771725	-2.741732	-0.994805
H	-2.070123	-2.632287	-0.627841
H	-3.704504	-0.075532	-0.781996
H	-3.312956	-0.908014	-2.267412
H	-0.959902	-0.345287	-2.126003
O	-2.328081	1.879054	-0.126287
C	-1.943967	3.264652	-0.094779
H	-2.534613	3.808605	-0.834501
H	-2.147328	3.650236	0.904234
H	-0.137623	3.993154	-1.118769
H	-0.001353	2.419930	1.518760
H	0.491587	4.111079	1.235609
C	3.726789	-0.364272	0.150624
C	3.064723	-1.062288	-1.036444
C	2.966728	-2.575176	-0.864873
C	1.855718	-2.996247	0.088130
H	1.873224	-4.064416	0.317635
H	1.913276	-2.468083	1.048412
H	0.864900	-2.838595	-0.395201
H	3.923150	-2.958499	-0.491853
H	2.805168	-3.051987	-1.835899
H	2.050898	-0.656374	-1.202347
H	3.619948	-0.822802	-1.949260
H	4.807233	-0.525654	0.099188
H	3.395088	-0.810822	1.093180
H	4.114266	1.627401	0.928920
H	3.644961	1.627240	-0.752490
Ag	-0.090052	-0.997472	0.159630

conf_14 7.5 kJ mol^{-1}

C	-3.721612	-0.228281	-0.593803
C	-2.455378	0.518644	-0.859885
O	-1.489878	0.064252	-1.450797
O	-2.515829	1.779368	-0.430644
C	-1.404115	2.637144	-0.666532
C	-0.707582	2.963001	0.642832
O	-0.285670	1.758764	1.305375
C	0.977508	1.508112	0.933846
C	1.608548	0.313005	1.149923
C	3.107931	0.161490	1.047360
C	3.578979	-1.271346	0.740439
C	4.020100	-1.521361	-0.699675
C	2.941663	-1.342655	-1.759211
H	3.299933	-1.594753	-2.760674
H	2.094212	-2.036116	-1.577399
H	2.602033	-0.296689	-1.819950
H	4.416671	-2.539121	-0.770359
H	4.856092	-0.853643	-0.938043
H	4.424740	-1.512186	1.390273
H	2.789624	-1.985428	1.013790
H	3.500769	0.856603	0.298074
H	3.540842	0.481728	2.002407
H	1.077563	-0.338054	1.843895
O	1.543438	2.563676	0.352598
C	0.622505	3.667272	0.415643
H	0.906921	4.313274	1.248276
H	0.685553	4.216670	-0.523763
H	-1.373105	3.484740	1.329584
H	-0.717007	2.172117	-1.377170
H	-1.800506	3.557341	-1.101646
C	-3.569511	-1.745453	-0.539275
C	-3.069746	-2.288286	0.796245
C	-1.650755	-1.890468	1.185321
C	-1.186514	-2.538751	2.480298
H	-0.162009	-2.253679	2.737156
H	-1.829230	-2.243438	3.314463
H	-1.217953	-3.629389	2.407560
H	-1.575957	-0.799466	1.283950
H	-0.972398	-2.195675	0.364247
H	-3.752950	-1.972051	1.595312
H	-3.128979	-3.381936	0.766724
H	-4.549515	-2.184397	-0.744191
H	-2.912885	-2.069856	-1.353641
H	-4.389843	0.053851	-1.418698
H	-4.183482	0.176073	0.311624
Ag	0.548984	-0.646966	-0.667558

conf_8 12.4 kJ mol^{-1}

C	-3.509241	-0.633873	-0.531098
C	-2.408740	0.283720	-0.947845
O	-1.357625	-0.069416	-1.454592
O	-2.682693	1.562256	-0.684027
C	-1.665819	2.522506	-0.948985
C	-1.008665	2.974237	0.344454
O	-0.529449	1.847272	1.100209
C	0.767538	1.672545	0.818180
C	1.493975	0.581663	1.214794
C	3.005250	0.581297	1.176718
C	3.648674	-0.800341	0.961491
C	4.177501	-1.052210	-0.448344
C	3.127200	-1.050842	-1.549775
H	2.627035	-0.076014	-1.637344
H	3.561134	-1.256953	-2.531722
H	2.388128	-1.861398	-1.388751

H	4.702243	-2.012815	-0.460161
H	4.932217	-0.292714	-0.683707
H	4.485582	-0.914356	1.656117
H	2.933721	-1.589126	1.236859
H	3.351303	1.275859	0.404695
H	3.359861	0.995140	2.127863
H	1.004598	-0.005538	1.992167
O	1.268385	2.694001	0.128848
C	0.280729	3.738648	0.080486
H	0.507967	4.470368	0.858146
H	0.330520	4.207941	-0.902043
H	-1.712225	3.503848	0.985946
H	-0.931026	2.101096	-1.638176
H	-2.155905	3.377678	-1.418770
C	-3.481479	-0.889763	0.985237
C	-2.245225	-1.608462	1.519665
C	-2.029104	-3.034940	0.994180
C	-0.992730	-3.166479	-0.116235
H	-0.880612	-4.199431	-0.455359
H	-1.245931	-2.564156	-0.995478
H	0.007558	-2.881646	0.271263
H	-2.983129	-3.441762	0.638873
H	-1.724885	-3.682058	1.821472
H	-1.347403	-0.998085	1.329674
H	-2.339593	-1.634341	2.609386
H	-3.601394	0.066034	1.504586
H	-4.368061	-1.484639	1.226992
H	-3.400215	-1.564730	-1.090187
H	-4.464981	-0.177062	-0.798613
Ag	0.604582	-0.815750	-0.397974

DIOXANE

conf_0 23.8 kJ mol^{-1}

C	-3.260566	1.449883	0.751596
C	-2.502298	0.159248	0.677975
O	-1.326438	0.031433	0.951817
O	-3.280569	-0.858547	0.307574
C	-2.780985	-2.196583	0.130362
C	-2.262047	-2.383968	-1.274622
H	-2.931367	-1.951601	-2.015810
H	-2.129023	-3.451091	-1.483636
O	-1.018450	-1.700984	-1.432858
C	-0.069008	-1.960640	-0.532327
O	-0.377858	-2.648462	0.547195
C	-1.709903	-2.678539	1.094687
H	-1.695523	-2.112989	2.025176
H	-1.884347	-3.731843	1.319013
C	1.236832	-1.573535	-0.766098
H	1.448609	-1.396555	-1.820536
C	2.359961	-2.134601	0.082773
C	3.688329	-1.389400	-0.032388
C	3.750669	-0.049113	0.685541
C	5.120959	0.607072	0.609101
H	5.136781	1.568451	1.128160
H	5.419605	0.778930	-0.429180
H	5.880108	-0.031429	1.070202
H	3.016766	0.655613	0.242973
H	3.454858	-0.181509	1.733539
H	4.469692	-2.031636	0.388197
H	3.949522	-1.255622	-1.089891
H	2.049830	-2.178090	1.131772
H	2.522683	-3.175658	-0.224187
H	-3.675834	-2.805611	0.267524
H	-3.821340	1.421477	1.694340
C	-2.338640	2.658543	0.703243
C	-1.731062	2.894946	-0.678078
C	-0.551402	3.862226	-0.656257
C	0.736841	3.212356	-0.167465
H	1.123952	2.528054	-0.957328
H	1.551459	3.922431	-0.008688
H	0.589269	2.706361	0.802117
H	-0.382563	4.271750	-1.655933
H	-0.790466	4.714456	-0.010714
H	-1.399329	1.939589	-1.118523
H	-2.508969	3.270092	-1.350965
H	-1.553749	2.526594	1.455044
H	-2.902920	3.546651	1.001711
H	-4.011681	1.465451	-0.043344
Ag	0.829243	0.585805	-0.187428

conf_48 27.8 kJ mol^{-1}

C	-2.477859	2.400977	0.610491
C	-2.338593	1.004930	0.084141
O	-1.515567	0.653008	-0.735113
O	-3.253972	0.181499	0.597589
C	-3.295394	-1.226114	0.301098
C	-2.848128	-1.683869	-1.076998
H	-2.944438	-0.909336	-1.835460
H	-3.451747	-2.545906	-1.366422
O	-1.491001	-2.168334	-1.119904
C	-0.635465	-1.968405	-0.137410
O	-1.091739	-1.772942	1.095226
C	-2.486789	-1.986356	1.323137
H	-2.674959	-1.635431	2.335785
H	-2.709962	-3.056475	1.259788
C	0.729167	-2.041273	-0.360746
H	0.966980	-2.402209	-1.361124
C	1.659144	-2.456817	0.766140
C	3.145120	-2.374229	0.422247
C	3.712564	-0.975592	0.204195
C	3.540020	-0.039054	1.392725
H	3.997654	-0.462620	2.291694
H	2.484945	0.147112	1.631343
H	4.012839	0.930091	1.210431
H	4.777793	-1.068335	-0.030374
H	3.271054	-0.528925	-0.704129
H	3.699184	-2.846199	1.241394
H	3.342996	-2.985744	-0.465769
H	1.444780	-1.880071	1.670084
H	1.434675	-3.500454	1.021809
H	-4.352331	-1.469589	0.421197
H	-2.632249	2.350589	1.693299
C	-1.294397	3.278051	0.233463
C	-0.019671	2.958355	1.013211
C	1.233700	3.536591	0.362830
C	1.709323	2.723397	-0.835122

H 0.885663 2.499792 -1.530300
H 2.474203 3.230600 -1.427797
H 2.225065 1.803447 -0.475954
H 1.031936 4.564267 0.041198
H 2.043634 3.598569 1.095007
H -0.119429 3.335914 2.036041
H 0.102655 1.866419 1.117530
H -1.561342 4.324575 0.406073
H -1.120008 3.180577 -0.842896
H -3.413897 2.797161 0.198528
Ag 0.927789 0.183251 -0.726676

conf_32 31.7 kJ mol^{-1}
C 3.598142 -0.703426 0.822887
C 2.485544 0.281340 0.625337
O 1.308324 0.081438 0.834664
O 2.971294 1.467422 0.241088
C 2.159200 2.644670 0.101628
C 1.556719 2.749306 -1.277892
H 2.270436 2.473327 -2.051866
H 1.213453 3.775082 -1.452722
O 0.461388 1.846305 -1.422972
C -0.483733 1.884446 -0.483406
O -0.267401 2.563334 0.623383
C 1.052963 2.839102 1.126620
H 1.197858 2.228922 2.017420
H 1.013693 3.889314 1.418273
C -1.707513 1.276954 -0.701036
H -1.923376 1.116282 -1.757327
C -2.881218 1.567489 0.211311
C -4.034166 0.571163 0.106401
C -3.776278 -0.794721 0.725516
C -4.990005 -1.710823 0.681060
H -4.774962 -2.686095 1.124080
H -5.324616 -1.873553 -0.347580
H -5.824264 -1.270709 1.235040
H -2.951420 -1.308853 0.187047
H -3.437207 -0.669817 1.761389
H -4.902022 1.009967 0.610544
H -4.328095 0.451300 -0.944082
H -2.540316 1.630399 1.249656
H -3.264107 2.564845 -0.039851
H 2.890007 3.446237 0.222576
H 4.156066 -0.321419 1.687211
C 3.174116 -2.149158 1.060393
C 3.016451 -2.984883 -0.209344
C 1.855619 -2.607595 -1.124265
C 0.490494 -2.914108 -0.520177
H -0.322986 -2.672621 -1.233411
H 0.354793 -3.978818 -0.309340
H 0.366763 -2.423107 0.465457
H 1.914894 -1.547852 -1.400971
H 1.950520 -3.162772 -2.062506
H 3.948890 -2.927581 -0.782606
H 2.908085 -4.037150 0.078035
H 2.259949 -2.163631 1.662962
H 3.942754 -2.621974 1.677365
H 4.291129 -0.611518 -0.020265
Ag -0.850484 -0.759037 -0.256894

conf_39 69.7 kJ mol^{-1}
C -3.584992 0.597531 0.967283
C -2.200797 1.109542 0.712861
O -1.195017 0.452699 0.925766
O -2.172814 2.342873 0.231873
C -0.936448 2.959691 -0.193380
C 0.103473 3.114363 0.925255
H 0.004817 2.323980 1.671435
H 0.024982 4.083858 1.412907
O 1.407263 3.081370 0.345978
C 1.739978 1.913382 -0.238539
O 0.724480 1.337238 -0.976553
C -0.288648 2.259385 -1.381551
H -1.019414 1.688679 -1.954721
H 0.161114 3.006944 -2.040918
C 2.947771 1.364540 -0.152929
H 3.639741 1.858125 0.521353
C 3.432744 0.197940 -0.955116
C 3.869741 -0.997379 -0.106388
C 2.725263 -1.677724 0.631314
C 3.137913 -2.844417 1.516871
H 3.647558 -3.615146 0.933207
H 2.278777 -3.304851 2.010209
H 3.825910 -2.501852 2.294190
H 2.214362 -0.921406 1.254125
H 2.033516 -2.092040 -0.151579
H 4.370712 -1.732179 -0.745145
H 4.611432 -0.670082 0.631449
H 2.664993 -0.117082 -1.674371
H 4.287233 0.521886 -1.560418
H -1.283603 3.937235 -0.528929
H -4.292091 1.148002 0.342244
C -3.646092 -0.908665 0.737641
C -3.423535 -1.295112 -0.725678
C -2.975430 -2.740947 -0.917124
C -1.512795 -2.969718 -0.553776
H -1.307633 -2.676235 0.486424
H -1.211210 -4.015030 -0.648949
H -0.868580 -2.410996 -1.267681
H -3.601768 -3.403851 -0.309674
H -3.130365 -3.041047 -1.957427
H -4.344100 -1.114089 -1.289388
H -2.666636 -0.637647 -1.182120
H -4.618955 -1.280567 1.070097
H -2.898717 -1.383615 1.380424
H -3.828401 0.841483 2.008831
Ag 0.316301 -0.908519 -0.138778

[PE(16:0/18:1) + Ag − 141]$^+$
Figure S5

conf_0 0.0 kJ mol^{-1}
C 1.862967 -2.304343 3.448164
C 1.415118 -0.901056 3.175833
O 1.958737 -0.106654 2.440572

Ag 1.920207 0.040731 -0.167530
C -0.265986 0.419727 -0.713147
C -0.760579 0.338393 0.564229
O -1.379534 -0.732910 1.047011
C -2.017810 -0.383714 2.287801
H -1.878905 -1.208650 2.983368
H -3.076687 -0.216196 2.088040
C -1.287745 0.893413 2.696580
H -1.977263 1.682704 3.001627
C -0.227213 0.721908 3.772642
H 0.580965 1.437969 3.611063
H -0.682714 0.896463 4.748434
O 0.293726 -0.605885 3.851759
O -0.716839 1.331954 1.446573
C -0.756376 -0.551635 -1.770183
H -0.062414 1.445011 -1.019629
H 2.928106 -2.361876 3.212558
H 1.728718 -2.503159 4.514037
C 1.080529 -3.340265 2.626841
H 3.594632 -3.141149 -2.357823
C 4.245885 -1.096209 -2.167932
C 3.356631 -2.167326 -2.8019
C 1.855521 -1.921609 -2.681322
C 1.388440 -0.576513 -3.224135
H 3.625910 -2.244098 -3.860861
C -0.120838 -0.367531 -3.146512
H -1.839805 -0.424906 -1.884335
H -0.620351 -1.581852 -1.429692
H -0.610940 -1.071332 -3.828873
H -0.362136 0.633720 -3.522595
H 1.896729 0.242309 -2.692127
H 1.704168 -0.477057 -4.269401
H 1.558499 -2.021075 -1.626917
H 1.322998 -2.725194 -3.204222
H 1.006359 -4.259058 -0.731997
H -1.062312 -3.035065 0.221378
H -1.294783 -4.418282 1.265947
C -0.868973 -4.109369 0.302508
C 0.641074 -4.321855 0.300457
H 0.885028 -5.334949 0.643537
C -3.093691 -4.541454 -0.874529
C 1.398153 -3.296655 1.139035
C -1.597281 -4.844624 -0.816898
H -1.139879 -4.574681 -1.779369
H -1.444653 -5.924201 -0.702163
H 1.170954 -2.291955 0.747819
H 2.478267 -3.440625 1.007746
H 0.008096 -3.202540 2.796717
H 1.327816 -4.332203 3.019436
H 4.807531 3.647346 -0.316712
H 2.711395 5.095113 -0.448540
H 4.650373 1.569465 -1.625778
C 4.053039 -0.908040 -0.691648
H 2.537655 2.724288 -1.136831
C 4.295017 0.232794 0.014501
H 0.284621 3.706588 0.800582
C 4.276104 2.790674 0.111578
C 4.819349 1.521680 -0.545172
C 0.171944 4.245995 -1.277613
C 2.247782 4.364859 0.226857
C 2.779625 2.973939 -0.095020
C 0.726628 4.453563 0.127715
H 4.509535 2.789857 1.183047
H 2.562150 4.641583 1.240179
H 5.910102 1.493243 -0.414125
H 2.232646 2.258812 0.544166
H 0.394634 5.430151 0.498229
H -4.702421 1.638467 1.472279
H -5.109395 -0.669494 0.558566
C -4.337217 1.994356 0.496979
H -5.719225 3.635864 0.384452
H -6.019402 1.300387 -0.656830
C -3.989779 4.297478 1.487584
C -4.657122 -0.350452 -0.392125
H -6.238592 -1.113246 -1.629388
C -4.633917 3.481935 0.376236
C -4.936904 1.130605 -0.604658
C -4.883058 -2.745068 -1.245575
C -5.160244 -1.264328 -1.503108
H -3.245683 1.856161 0.504766
C -3.398963 -3.087297 -1.212273
H -3.575279 -0.484814 -0.275837
H -4.283341 3.842480 -0.597941
H -4.531946 1.446698 -1.575258
H -4.699382 -0.966573 -2.455149
H -3.554287 -4.795796 0.089729
H -3.566983 -5.194520 -1.616843
H 4.131353 -0.144906 -2.694886
H 5.294166 -1.388716 -2.318000
H -5.377770 -3.347160 -2.016471
H -5.344102 -3.036620 -0.291952
H -2.953387 -2.844926 -2.187904
H -2.900546 -2.440532 -0.484091
H -4.206604 5.363795 1.384921
H -2.900053 4.181433 1.480166
H -4.355631 3.981937 2.470853
C -1.347329 4.147784 -1.316340
H 0.592591 3.333609 -1.722164
H 0.505328 5.064288 -1.928811
C -1.890736 3.860342 -2.707530
H -1.781635 5.078276 -0.931373
H -1.671507 3.359579 -0.624238
H -2.982493 3.800025 -2.711260
H -1.506501 2.909213 -3.093291
H -1.600724 4.641909 -3.416235
H 3.875653 -1.819025 -0.117559
H 4.272063 0.160656 1.102887

conf_1 0.8 kJ mol^{-1}

C 1.054118 -1.391717 3.822736
C 1.450425 -0.334089 2.838601
O 2.045414 -0.513335 1.799858
Ag 1.092455 -1.499862 -0.491296
C -0.338168 0.111816 -1.217902
C -0.300187 0.911010 -0.100446
O -1.253920 0.928300 0.821023

C	-1.034126	2.045039	1.701727
H	-1.202941	1.709028	2.723050
H	-1.741140	2.831031	1.431549
C	0.417919	2.431354	1.416606
H	0.531191	3.499937	1.226747
C	1.434505	2.019985	2.467212
H	2.399711	1.842196	1.988412
H	1.531253	2.825377	3.196881
O	1.042105	0.882428	3.236047
O	0.665589	1.780530	0.155386
C	-1.667027	-0.476307	-1.661317
H	0.346207	0.455794	-1.994122
H	1.743790	-2.230166	3.700087
H	1.168650	-0.986934	4.830583
C	-0.392448	-1.862195	3.612488
H	-0.416198	-5.456398	-1.475272
C	1.399945	-4.411735	-1.956218
C	-0.082235	-4.701382	-2.196644
C	-0.996903	-3.484575	-2.101789
C	-0.666351	-2.378175	-3.094943
H	-0.188251	-5.159481	-3.185865
C	-1.602223	-1.177035	-3.015902
H	-2.403525	0.333335	-1.733923
H	-2.060393	-1.157570	-0.901237
H	-2.617602	-1.498747	-3.274042
H	-1.312998	-0.446293	-3.780388
H	0.371246	-2.040916	-2.953001
H	-0.700150	-2.786260	-4.111893
H	-0.966437	-3.088868	-1.075646
H	-2.033599	-3.806754	-2.255513
H	-2.031659	-3.698873	1.145222
H	-2.602183	-1.192094	1.313602
H	-3.193716	-1.510618	2.929834
C	-3.029309	-1.975902	1.949352
C	-2.017304	-3.108716	2.070484
H	-2.315558	-3.797841	2.870369
C	-5.390540	-1.289614	1.268248
C	-0.592770	-2.623649	2.310558
C	-4.373278	-2.420431	1.387428
H	-4.218134	-2.874366	0.398372
H	-4.783706	-3.214100	2.023169
H	-0.306167	-1.975856	1.465595
H	0.098656	-3.475829	2.293864
H	-1.064346	-0.999689	3.662071
H	-0.659775	-2.508736	4.454996
H	5.757261	-1.600833	-1.161617
H	5.784200	1.005708	-0.669671
H	3.689122	-2.744415	-2.180394
C	1.689953	-3.821810	-0.608262
H	4.059701	-0.142082	-2.145996
C	2.802999	-3.121421	-0.252766
H	4.192156	2.465952	-1.845530
C	4.765091	-1.575861	-0.699521
C	3.984234	-2.817114	-1.127637
C	4.475023	3.452779	0.046328
C	4.720754	0.954667	-0.403975
C	4.083919	-0.263128	-1.055137
C	4.026527	2.256287	-0.780832
H	4.932549	-1.617513	0.384228
H	4.685866	0.822770	0.685787
H	4.644385	-3.693603	-1.065137
H	3.029599	-0.289800	-0.728513
H	2.943467	2.127186	-0.665437
H	-2.880995	5.501302	-2.114131
H	-4.477605	3.201946	0.363624
C	-2.884036	4.626120	-1.453846
H	-1.618623	3.451414	-2.733546
H	-5.021693	4.523792	-1.664889
C	-0.342096	4.631898	-1.463057
C	-4.418673	2.743776	-0.634843
H	-6.534138	2.663145	-0.976541
C	-1.610563	3.826967	-1.702501
C	-4.169671	3.834244	-1.670345
C	-6.027018	0.909959	0.159293
C	-5.697915	1.958109	-0.898909
H	-2.870488	5.025528	-0.429377
C	-5.000092	-0.209869	0.266318
H	-3.566196	2.054788	-0.606118
H	-1.609959	2.933798	-1.066581
H	-4.153007	3.385731	-2.673004
H	-5.623420	1.470813	-1.880798
H	-5.541479	-0.832292	2.255581
H	-6.361634	-1.708524	0.979664
H	1.810710	-3.781258	-2.750812
H	1.954225	-5.358146	-2.020959
H	-7.007944	0.473870	-0.064518
H	-6.131326	1.399864	1.137034
H	-4.865183	-0.670567	-0.723291
H	-4.027113	0.212817	0.539582
H	-0.286794	5.488607	-2.141011
H	0.558536	4.030424	-1.614671
H	-0.315390	5.035591	-0.443650
C	3.704827	4.727376	-0.272192
H	5.548154	3.627443	-0.102916
H	4.361768	3.219795	1.115661
C	4.131926	5.915095	0.576768
H	2.630122	4.542137	-0.136992
H	3.830278	4.968662	-1.334798
H	3.561224	6.813945	0.328863
H	5.191263	6.144697	0.427425
H	3.989463	5.712962	1.643913
H	1.027149	-4.139737	0.198027
H	2.945250	-2.916635	0.809430

[PE(18:1/16:0) + Ag − 141]$^{+}$
Figure S5

conf_1 0.0 kJ mol^{-1}

C	2.628789	3.163192	0.671087
C	2.212444	1.739727	0.452649
O	2.674537	0.800911	1.055755
Ag	2.141684	-1.422136	-0.346948
C	0.541095	-1.898664	-1.932783
C	-0.048843	-0.662442	-1.872538
O	-1.018506	-0.351574	-1.022315

C	-1.414422	1.007302	-1.223260
H	-1.455262	1.497337	-0.251082
H	-2.402663	1.013693	-1.685788
C	-0.324936	1.558666	-2.141101
H	-0.724694	2.135319	-2.976024
C	0.751074	2.346392	-1.427672
H	1.530239	2.640796	-2.134758
H	0.322326	3.234777	-0.952589
O	1.279095	1.438130	-0.469463
O	0.260867	0.358825	-2.676804
C	-0.164072	-3.120212	-1.373086
H	1.175396	-2.034427	-2.806629
H	1.745265	3.797337	0.793022
H	3.166304	3.162640	1.619495
C	3.525295	3.716030	-0.457933
H	-3.716435	-0.683638	-2.813578
C	-5.002445	-0.811168	-1.101276
C	-4.253076	-1.464697	-2.253621
C	-3.253186	-2.519523	-1.794554
C	-2.252009	-2.910056	-2.874854
H	-4.962195	-1.899021	-2.968997
C	-1.105433	-3.788310	-2.386482
H	-0.715716	-2.844521	-0.468939
H	0.579541	-3.859940	-1.061603
H	-0.513328	-4.121448	-3.246869
H	-1.508889	-4.696634	-1.923176
H	-2.773373	-3.429899	-3.686592
H	-1.841313	-1.999636	-3.333847
H	-3.786929	-3.412107	-1.444989
H	-2.718101	-2.126442	-0.923889
H	6.215449	2.945346	0.197123
H	7.187783	0.770956	-0.021319
H	6.706656	1.250273	-1.634541
C	6.312702	0.965940	-0.651866
C	5.522787	2.141135	-0.074930
H	5.036096	1.831873	0.856030
C	5.226682	-1.020744	0.542317
C	4.490523	2.695770	-1.053385
C	5.507226	-0.324837	-0.789119
H	4.552456	-0.117885	-1.293715
H	6.040986	-1.019981	-1.446968
H	3.911194	1.869864	-1.490178
H	5.012900	3.160143	-1.897074
H	4.081182	4.562449	-0.043020
H	2.903391	4.133931	-1.254884
H	-7.289391	0.358577	0.143971
H	-5.215589	3.558334	-0.308597
H	-3.153235	5.048111	0.268976
H	-0.159835	5.325057	-0.215405
H	-6.231365	2.151552	1.413306
C	-5.953131	0.293914	-1.542088
H	-4.008654	3.452833	1.922187
C	-6.621185	1.044980	-0.389905
H	-1.735047	4.596909	2.326690
C	-4.672736	2.665425	0.027245
C	-5.654605	1.662238	0.619945
C	-0.522257	5.507414	0.804019
C	-2.611260	4.107512	0.427280
C	-3.567631	3.066855	0.994644
C	-1.397050	4.368979	1.309190
H	-1.083838	6.445400	0.772697
H	-7.264380	1.830107	-0.805098
H	-4.212139	2.244632	-0.875998
H	-2.277566	3.797598	-0.575914
H	0.347212	5.671674	1.447315
H	-5.091855	0.863779	1.117522
H	-3.007559	2.167853	1.292082
H	-0.807296	3.443941	1.401070
H	-1.032513	-2.710038	2.851468
H	-3.202259	-0.843496	4.000829
H	1.472267	-3.406336	3.067157
H	-5.834862	-1.412643	2.268186
C	-0.721663	-1.678076	3.053213
H	-1.677448	-0.786625	1.348576
H	0.676307	-1.650975	1.440824
C	-3.157170	-0.885582	2.904577
H	-3.301535	-3.040985	2.857380
C	1.739981	-2.348954	3.191700
H	3.415193	-1.055229	2.731294
C	-5.293464	-2.240326	2.738577
C	-1.716710	-0.698755	2.442241
C	0.691211	-1.466963	2.531451
C	-3.808092	-2.174876	2.416255
C	3.409559	-2.654199	1.299631
C	3.160551	-2.117428	2.676372
H	-0.726628	-1.565753	4.145363
H	-5.467467	-2.175605	3.817219
H	-3.754581	-0.036273	2.551142
C	4.312229	-2.196132	0.389438
H	1.727311	-2.160129	4.270480
H	-5.741230	-3.173585	2.386656
H	-1.388783	0.322293	2.681399
H	0.978791	-0.415844	2.650614
H	-3.664936	-2.258189	1.331196
H	3.864719	-2.638579	3.340035
H	4.818265	-0.317207	1.270484
H	6.176319	-1.386261	0.958220
H	-6.732037	-0.134057	-2.183682
H	-4.261982	-0.405971	-0.398525
H	-5.408405	1.002104	-2.180930
H	-5.557166	-1.571621	-0.536115
H	4.498911	-2.828210	-0.480871
H	2.938296	-3.615387	1.083068

conf_8 2.7 kJ mol^{-1}

C	3.175792	3.167500	-0.183254
C	2.440852	1.886978	0.081216
O	2.888587	0.992299	0.758122
Ag	2.089908	-1.401216	-0.097674
C	0.624923	-1.861116	-1.788775
C	0.011838	-0.630616	-1.777998
O	-0.980150	-0.310836	-0.960633
C	-1.409568	1.027855	-1.226169
H	-1.459127	1.562563	-0.279003
H	-2.398130	0.985967	-1.685215
C	-0.334529	1.565593	-2.171498

H -0.753743 2.054813 -3.051792
C 0.685626 2.463254 -1.509122
H 1.459414 2.742370 -2.226498
H 0.200593 3.362871 -1.116994
O 1.225224 1.677177 -0.454207
O 0.323932 0.363891 -2.609937
C -0.110007 -3.091073 -1.282358
H 1.292836 -1.993821 -2.638093
H 2.481063 4.005875 -0.282477
H 3.781511 3.342518 0.707350
C 4.092039 3.085897 -1.424882
H -3.545004 -0.610415 -2.896712
C -4.931013 -0.737177 -1.262351
C -4.125052 -1.388758 -2.377411
C -3.168406 -2.464514 -1.875665
C -2.109004 -2.852849 -2.899952
H -4.797739 -1.802351 -3.138650
C -0.996271 -3.741736 -2.355486
H -0.708152 -2.829693 -0.404529
H 0.615258 -3.836657 -0.943955
H -0.357997 -4.069212 -3.184485
H -1.429655 -4.652747 -1.925693
H -2.585824 -3.362912 -3.744586
H -1.668368 -1.941525 -3.328844
H -3.733352 -3.355009 -1.573257
H -2.682542 -2.092252 -0.967620
H 6.629995 2.285892 -0.417537
H 7.340514 0.069043 0.198967
H 7.030781 0.072723 -1.524218
C 6.539782 0.140362 -0.546355
C 5.860474 1.505228 -0.425086
H 5.345227 1.572623 0.538071
C 5.088862 -1.258484 1.044174
C 4.883504 1.789605 -1.561823
C 5.601434 -1.053113 -0.382407
H 4.742188 -0.942624 -1.058916
H 6.112894 -1.965090 -0.710376
H 4.178649 0.955386 -1.668600
H 5.436890 1.827276 -2.507168
H 4.773801 3.940733 -1.372504
H 3.498727 3.233895 -2.333323
H -7.273255 0.464582 -0.165056
H -5.121112 3.664283 -0.260423
H -2.982510 5.031814 0.601249
H -0.010066 5.179496 0.115610
H -6.284307 2.168882 1.256499
C -5.806124 0.415399 -1.737835
H -4.001956 3.323220 1.998010
C -6.546953 1.149396 -0.619203
H -1.588816 4.172384 2.556295
C -4.620129 2.728214 0.017275
C -5.653580 1.709963 0.486634
C -0.319619 5.211285 1.167486
C -2.513840 4.040288 0.620772
C -3.541118 3.002274 1.055652
C -1.276341 4.078368 1.509669
H -0.792525 6.184926 1.324438
H -7.135268 1.963272 -1.059710
H -4.136149 2.370992 -0.901173
H -2.211433 3.847076 -0.420959
H 0.582841 5.180398 1.784576
H -5.137318 0.883658 0.989594
H -3.033238 2.055998 1.292586
H -0.753316 3.112608 1.448321
H -1.234582 0.009127 3.851584
H -3.570865 -1.086111 3.670183
H 0.723203 -2.220732 3.487415
H -6.037557 -1.435930 1.774803
C -1.077524 -0.216204 2.788540
H -1.519400 -2.327942 2.966576
H 0.533853 -0.664034 1.462539
C -3.369494 -1.293396 2.611004
H -3.859877 -3.383653 2.727712
C 1.183676 -1.225859 3.453316
H 3.117364 -0.392179 2.926999
C -5.682525 -2.298636 2.348273
C -1.871648 -1.456621 2.400558
C 0.415710 -0.315903 2.503936
C -4.185472 -2.499015 2.166365
C 2.938169 -2.304795 1.966411
C 2.666919 -1.372249 3.107874
H -1.484783 0.642889 2.240971
H -5.932402 -2.119521 3.398677
H -3.712709 -0.404903 2.063568
C 3.989593 -2.273475 1.101430
H 1.097667 -0.819012 4.466655
H -6.249058 -3.173359 2.017936
H -1.680280 -1.678271 1.341767
H 0.871072 0.679523 2.531432
H -3.967257 -2.711272 1.112208
H 3.189760 -1.797468 3.975974
H 4.752155 -0.315088 1.477110
H 5.915455 -1.616943 1.673483
H -6.541080 0.037054 -2.457763
H -4.229855 -0.380940 -0.495541
H -5.187507 1.125855 -2.303137
H -5.552729 -1.490929 -0.762451
H 4.127969 -3.151373 0.467118
H 2.316872 -3.202941 1.947146

conf_0 8.8 kJ mol^{-1}

C 1.453861 3.639598 0.139837
C 1.318868 2.149147 0.137291
O 2.061720 1.408878 0.746121
Ag 2.119842 -0.843019 -0.255586
C 0.527246 -1.993537 -1.530716
C -0.381224 -0.974364 -1.582897
O -1.374353 -0.820640 -0.714352
C -2.150400 0.322915 -1.080396
H -2.322919 0.918465 -0.186234
H -3.102787 -0.017150 -1.489833
C -1.276839 1.024861 -2.116470
H -1.838210 1.359186 -2.989715
C -0.457135 2.165363 -1.552792
H 0.208747 2.572461 -2.318914
H -1.114359 2.951282 -1.167939

O 0.302813 1.562761 -0.508648
O -0.394285 -0.028654 -2.534183
C 0.254764 -3.243669 -0.720927
H 1.130645 -2.087773 -2.431378
H 0.884130 4.092837 -0.675147
H 0.964819 3.961326 1.068769
C 2.907988 4.111447 0.123529
H -4.353866 -2.776032 -1.966570
C -5.321142 -3.378645 -0.124237
C -4.319834 -3.611966 -1.254874
C -2.884898 -3.817312 -0.782195
C -1.904166 -3.973468 -1.938329
H -4.629151 -4.494179 -1.827570
C -0.481597 -4.326947 -1.519155
H -0.311113 -2.983216 0.178534
H 1.202974 -3.666207 -0.372299
H 0.110749 -4.567489 -2.410154
H -0.503638 -5.240018 -0.912017
H -2.268864 -4.760586 -2.608908
H -1.898826 -3.056207 -2.543801
H -2.843822 -4.714334 -0.149628
H -2.578133 -2.979687 -0.146989
H 2.671784 2.288226 -2.099825
H 4.091003 1.440869 -0.271211
H 5.503611 2.217103 -0.951817
C 4.554795 1.734566 -1.218934
C 3.666152 2.736106 -1.944428
H 4.060815 2.895928 -2.955003
C 5.447519 -0.649509 -1.209522
C 3.543663 4.098207 -1.265514
C 4.854394 0.492463 -2.052080
H 3.936474 0.142503 -2.545137
H 5.541460 0.759491 -2.860917
H 2.973814 4.770942 -1.918744
H 4.544138 4.538256 -1.186136
H 3.483824 3.502530 0.826191
H 2.933111 5.134889 0.507223
H -6.710240 -0.982195 -0.567575
H -6.918577 1.611986 -0.759520
H -3.674039 3.372588 -0.993642
H -1.653327 4.247413 1.190037
H -6.066882 0.552332 1.283391
C -5.205958 -2.020954 0.567318
H -6.057553 3.820582 -0.532350
C -5.654455 -0.850057 -0.298199
H -4.252239 5.522452 0.163894
C -5.847156 1.675136 -0.538566
C -5.467091 0.507488 0.365474
C -2.215735 5.030128 0.667314
C -4.040747 3.383470 0.044935
C -5.527736 3.050912 0.040550
C -3.706465 4.725085 0.681471
H -1.836680 5.098104 -0.359654
H -5.110404 -0.861155 -1.252861
H -5.340186 1.568096 -1.509526
H -3.488472 2.601415 0.580891
H -1.991091 5.980757 1.157746
H -4.424709 0.603643 0.695651
H -5.916920 3.114251 1.065034
H -4.070458 4.730213 1.715637
H 1.317815 -2.508518 4.080120
H -0.071870 -0.641308 1.820953
H 3.661178 -2.991098 3.022908
H -2.833972 1.768963 2.648670
C 1.560866 -1.439238 4.026457
H 0.534234 0.443905 3.975362
H 1.968912 -1.519972 1.933879
C -0.596974 -0.922581 2.743483
H -2.501111 -0.505990 3.661087
C 3.827684 -1.909780 2.934418
H 4.968410 -0.566717 1.675369
C -1.845106 1.304435 2.700908
C 0.273894 -0.621607 3.959156
C 2.492286 -1.189660 2.849659
C -1.947519 -0.213973 2.760617
C 4.160157 -2.126069 0.440810
C 4.736166 -1.635364 1.736086
H 2.090451 -1.199096 4.956107
H -1.272553 1.622261 1.820823
H -0.763381 -2.006306 2.683954
C 4.452180 -1.694334 -0.810376
H 4.347508 -1.606199 3.849218
H -1.342556 1.714597 3.580832
H -0.312986 -0.806292 4.867115
H 2.660276 -0.111643 2.736270
H -2.544235 -0.572917 1.914616
H 5.694718 -2.148811 1.889762
H 5.956290 -0.239873 -0.332226
H 6.216935 -1.172934 -1.789935
H -4.171541 -1.856758 0.897068
H -5.199874 -4.174650 0.620342
H -5.808482 -2.032428 1.483182
H -6.339547 -3.486133 -0.516510
H 4.034226 -2.266813 -1.640006
H 3.544979 -3.025799 0.517869

[PC(18:1(9*Z*)/18:1(9*Z*)) + Ag − 183]$^+$
Figure S7

conf_0 0.0 kJ mol^{-1}

C -2.319596 1.444441 3.693573
C -1.621748 0.197594 3.246124
O -2.018474 -0.585625 2.412010
Ag -2.052633 -0.319086 -0.200647
C 0.142016 -0.205842 -0.811582
C 0.653850 -0.192198 0.462975
O 1.076380 0.907366 1.071081
C 1.780399 0.542781 2.269809
H 1.469775 1.218598 3.064005
H 2.848278 0.637274 2.075144
C 1.350468 -0.906158 2.498469
H 2.203085 -1.566089 2.669765
C 0.332752 -1.118203 3.607335
H -0.312327 -1.963267 3.358883
H 0.860975 -1.321369 4.539832

O -0.451469 0.042677 3.883515
O 0.819319 -1.278503 1.210731
C 0.411844 0.968656 -1.732865
H 0.129436 -1.203960 -1.247935
H -3.371299 1.354690 3.412776
H -2.251809 1.499515 4.782950
C -1.708643 2.709416 3.073700
H -4.341434 2.790554 -1.886729
C -4.614573 0.655611 -1.981919
C -3.952981 1.946200 -2.468463
C -2.428431 1.959620 -2.404581
C -1.755438 0.806987 -3.140306
H -4.272747 2.118599 -3.501843
C -0.230813 0.859319 -3.113109
H 1.495005 1.064534 -1.872639
H 0.106838 1.901986 -1.253078
H 0.104117 1.723902 -3.697744
H 0.165890 -0.023942 -3.627836
H -2.092690 -0.154375 -2.723235
H -2.089815 0.798144 -4.184503
H -2.112589 1.964503 -1.350978
H -2.065946 2.909594 -2.815606
H -1.846708 4.220983 -0.050598
H 0.445092 3.302809 0.252084
H 0.574555 4.077240 1.813482
C 0.101235 4.169415 0.827334
C -1.415886 4.111861 0.952309
H -1.779460 4.973205 1.527958
C 2.028331 5.335646 -0.363657
C -1.952374 2.826922 1.575517
C 0.595521 5.444647 0.154635
H -0.061288 5.683663 -0.692807
H 0.511209 6.288081 0.848482
H -1.503787 1.966054 1.056144
H -3.033795 2.765282 1.398811
H -0.638131 2.744448 3.299118
H -2.155211 3.573596 3.576494
H -4.273195 -4.325886 -0.800881
H -1.948443 -5.367187 -1.071934
H -4.497175 -2.095302 -1.819988
C -4.335130 0.302649 -0.550384
H -2.206245 -2.941125 -1.475972
C -4.346553 -0.949684 -0.011698
H 0.197623 -3.730151 0.373950
C -3.891345 -3.454467 -0.258125
C -4.654226 -2.222197 -0.743855
C 0.400160 -3.992160 -1.749370
C -1.618733 -4.652081 -0.307554
C -2.388661 -3.347673 -0.472133
C -0.106292 -4.459437 -0.388792
H -4.106084 -3.635392 0.801976
H -1.875670 -5.097829 0.660729
H -5.731606 -2.398773 -0.618095
H -1.968263 -2.626758 0.250663
H 0.395006 -5.398640 -0.128040
H 4.884874 -0.541413 -1.539796
H 5.188340 1.860857 -2.287759
C 5.018651 -0.203534 -0.503828
H 3.266872 -1.177649 0.261704
H 3.401602 1.220585 -0.587919
C 4.838117 -2.629314 0.303653
C 5.184006 2.225989 -1.252071
H 4.489488 3.978879 -0.186852
C 4.351455 -1.194581 0.437919
C 4.476704 1.212634 -0.363383
C 3.204246 3.659493 -1.877462
C 4.548712 3.615134 -1.219288
H 6.100962 -0.202281 -0.323115
C 2.144485 4.385302 -1.518991
H 6.235351 2.297272 -0.951652
H 4.508969 -0.863460 1.475652
H 4.571358 1.539505 0.682796
H 5.212140 4.315151 -1.744753
H 2.705146 5.045273 0.448718
H 2.362248 6.329592 -0.689557
H -4.354635 -0.177778 -2.640467
H -5.703020 0.772483 -2.074836
C 1.875012 -3.613698 -1.739764
H -0.181139 -3.126390 -2.094704
H 0.223821 -4.776065 -2.497168
C 2.366173 -3.073047 -3.073569
H 2.471015 -4.487433 -1.449607
H 2.043084 -2.864806 -0.955491
H 1.815068 -2.170512 -3.361502
H 2.233039 -3.807268 -3.873832
H 3.427771 -2.812888 -3.036919
H -4.299251 1.142158 0.146069
H -4.296632 -1.026296 1.075394
H 3.118148 3.061002 -2.785658
H 1.263266 4.332820 -2.160458
C 4.153821 -3.581393 1.276301
H 5.923636 -2.665571 0.459601
H 4.667542 -2.975741 -0.723621
C 4.593491 -5.027913 1.109491
H 5.671272 -5.132932 1.266294
H 4.371610 -5.391071 0.100969
H 4.088724 -5.688577 1.819412
H 3.066003 -3.512773 1.139135
H 4.359688 -3.252602 2.304468

conf_29 1.9 kJ mol^{-1}

C 1.559138 -1.808197 3.682818
C 1.810977 -0.482364 3.028059
O 2.541792 -0.291362 2.081414
Ag 1.801896 -0.985759 -0.333212
C 0.065520 0.370576 -0.882145
C -0.095432 0.979697 0.340896
O -1.076427 0.681869 1.179142
C -1.065597 1.611432 2.275916
H -1.185543 1.045358 3.197755
H -1.892786 2.306278 2.134291
C 0.299286 2.287125 2.148257
H 0.223504 3.375895 2.174675
C 1.340428 1.847527 3.163215
H 2.341097 1.958029 2.741236
H 1.246408 2.469125 4.055116

O 1.138225 0.510994 3.626137
O 0.687554 1.947591 0.801774
C -1.092894 -0.402305 -1.4852
H 0.692498 0.952526 -1.558519
H 2.027526 -2.570617 3.055841
H 2.101503 -1.793144 4.635799
C 0.081603 -2.106112 3.929011
H 1.054865 -5.044296 -1.294019
C 2.671717 -3.739537 -1.860574
C 1.247045 -4.263837 -2.039642
C 0.151782 -3.206308 -1.945719
C 0.317553 -2.042945 -2.915641
H 1.188736 -4.757250 -3.015847
C -0.805104 -1.011649 -2.855250
H -1.945681 0.277234 -1.599917
H -1.435682 -1.178078 -0.794873
H -1.733407 -1.478121 -3.203032
H -0.586650 -0.206931 -3.567306
H 1.282196 -1.541576 -2.741697
H 0.381023 -2.432866 -3.938530
H 0.095587 -2.829352 -0.912674
H -0.815230 -3.688842 -2.124948
H -2.509194 -2.974284 3.642185
H -2.853441 -1.543894 0.955788
H -4.153881 -2.089560 1.987837
C -3.123416 -2.315271 1.686693
C -2.230832 -2.198142 2.916837
H -2.438670 -1.241377 3.413818
C -4.172830 -3.833411 -0.065972
C -0.732000 -2.279202 2.649782
C -3.110604 -3.688750 1.027692
H -2.121375 -3.902085 0.601217
H -3.285539 -4.453881 1.794163
H -0.464992 -1.501089 1.922360
H -0.475667 -3.237049 2.180970
H -0.343176 -1.297710 4.532759
H 0.007540 -3.011470 4.539140
H 6.089671 0.134289 -1.706058
H 5.322657 2.595748 -1.729893
H 4.382090 -1.554833 -2.305879
C 2.939137 -3.082726 -0.539077
H 3.638975 0.823699 -1.868926
C 3.882459 -2.129344 -0.296001
H 3.191572 3.154974 0.392904
C 5.329869 -0.145503 -0.968227
C 4.828722 -1.547824 -1.305347
C 2.615160 3.452861 -1.650334
C 4.766282 2.335781 -0.820405
C 4.235167 0.912059 -0.950445
C 3.680364 3.376791 -0.565587
H 5.839656 -0.161122 0.003035
H 5.492638 2.368561 0.000631
H 5.688986 -2.229618 -1.361143
H 3.556092 0.725001 -0.102759
H 4.148051 4.361619 -0.449919
H -4.433054 2.345680 -1.936261
H -5.698537 0.329596 -2.516738
C -4.674839 2.218361 -0.872551
H -2.643373 2.279919 -0.205899
H -3.906569 0.220607 -0.686022
C -3.313266 4.316936 -0.305948
C -5.895797 0.059164 -1.470623
H -6.081301 -1.760620 -0.313508
C -3.570841 2.843188 -0.032197
C -4.868003 0.737440 -0.575724
C -4.595987 -2.054227 -1.838075
C -5.883695 -1.465617 -1.349245
H -5.615344 2.759660 -0.709777
C -3.874661 -3.036209 -1.296100
H -6.897789 0.439139 -1.242780
H -3.819307 2.710197 1.030967
H -5.155409 0.604414 0.476255
H -6.708611 -1.872644 -1.948531
H -5.152393 -3.568569 0.344089
H -4.238698 -4.890457 -0.352912
H 2.941504 -3.070508 -2.682606
H 3.364124 -4.589168 -1.938136
C 1.550215 4.508843 -1.387626
H 2.122446 2.477877 -1.748201
H 3.090676 3.646204 -2.621297
C 0.403049 4.449484 -2.385175
H 2.010722 5.503609 -1.407683
H 1.159239 4.375620 -0.370914
H -0.334381 5.234772 -2.201292
H -0.118154 3.486670 -2.329819
H 0.764224 4.569553 -3.411297
H 2.464742 -3.548433 0.327136
H 4.087046 -1.880448 0.745626
H -4.226209 -1.618439 -2.768040
H -2.962725 -3.328645 -1.816907
C -2.147735 4.876341 0.500630
H -4.219179 4.896084 -0.087555
H -3.111017 4.460503 -1.375377
C -1.909618 6.361200 0.271664
H -2.781962 6.948904 0.572274
H -1.722446 6.571502 -0.785692
H -1.050053 6.727748 0.839733
H -1.238050 4.315285 0.242835
H -2.334302 4.701066 1.570147

conf_14 5.7 kJ mol^{-1}

C 0.680035 0.153209 3.260575
C -0.530115 0.552095 2.482482
O -0.589508 0.616379 1.273439
Ag -0.459361 -1.059861 -0.602234
C -2.284953 -2.339603 -0.095941
C -2.730960 -1.626372 0.987288
O -2.578415 -2.036700 2.242975
C -3.434948 -1.243908 3.085182
H -2.911879 -1.054986 4.020014
H -4.353571 -1.805344 3.264798
C -3.668329 0.006867 2.243177
H -4.713440 0.319506 2.237330
C -2.803886 1.203663 2.611046
H -2.600186 1.805987 1.723872
H -3.332211 1.807438 3.349809

O	-1.583673	0.832141	3.258865
O	-3.396938	-0.479444	0.912214
C	-1.911643	-3.800547	0.089678
H	-2.766471	-2.042458	-1.028685
H	1.430332	0.932876	3.087987
H	0.446578	0.145304	4.326919
C	1.217917	-1.205682	2.803356
H	3.039316	-3.393728	-1.162527
C	2.027464	-2.031660	-2.485660
C	2.183527	-3.412319	-1.847769
C	0.955455	-3.913736	-1.094391
C	-0.311760	-3.971027	-1.938562
H	2.440947	-4.126607	-2.637230
C	-1.529238	-4.520218	-1.201972
H	-2.765781	-4.330383	0.529156
H	-1.104472	-3.891674	0.823201
H	-1.348549	-5.571882	-0.952708
H	-2.389259	-4.515428	-1.881928
H	-0.546145	-2.970943	-2.332750
H	-0.126967	-4.592160	-2.822987
H	0.790720	-3.276993	-0.211088
H	1.167774	-4.911732	-0.693227
H	3.512925	0.281608	3.311484
H	3.533953	-0.923393	1.067423
H	4.596899	-2.128021	1.774126
C	4.304643	-1.070507	1.833992
C	3.727246	-0.791274	3.215996
H	4.498038	-1.000873	3.966053
C	6.208513	-0.545436	0.191822
C	2.485320	-1.615421	3.550052
C	5.498224	-0.185487	1.502015
H	5.164065	0.857417	1.454745
H	6.227430	-0.239671	2.319436
H	2.288543	-1.563714	4.627468
H	2.692090	-2.670035	3.333156
H	1.405199	-1.169664	1.724250
H	0.441878	-1.963912	2.959416
H	-0.735473	2.071048	-4.064565
H	-3.121168	2.575194	-3.471818
H	0.200467	-0.203616	-3.678877
C	1.745283	-0.929556	-1.511007
H	-2.158834	0.384958	-2.826250
C	1.110585	0.244402	-1.783461
H	-3.900302	1.920570	-0.590988
C	-0.503285	1.742923	-3.045579
C	0.570405	0.662736	-3.118749
C	-4.882839	0.826333	-2.177120
C	-2.868712	2.385268	-2.420469
C	-1.791506	1.309225	-2.360094
C	-4.143362	2.054381	-1.652803
H	-0.097964	2.621763	-2.528895
H	-2.446947	3.321351	-2.036237
H	1.422110	1.047243	-3.698119
H	-1.594256	1.076500	-1.300655
H	-4.815573	2.920135	-1.705325
H	2.679296	4.617224	-0.215722
H	4.716210	3.820630	-1.759238
C	2.387432	3.560355	-0.167924
H	2.336087	3.414788	1.968419
H	4.318206	2.901397	0.507633
C	0.404579	4.146599	1.374947
C	4.337585	2.799520	-1.637947
H	6.042425	2.036343	-2.707998
C	1.639200	3.286112	1.130142
C	3.616155	2.673157	-0.305520
C	5.016954	0.391933	-1.843952
C	5.496740	1.807198	-1.784414
H	1.723852	3.393448	-1.026757
C	5.303595	-0.604987	-1.005094
H	3.622738	2.639686	-2.457290
H	1.341282	2.229480	1.136278
H	3.323395	1.624695	-0.158709
H	6.203124	1.946272	-0.959917
H	6.692684	-1.521697	0.310447
H	7.014797	0.174193	0.021970
H	1.273713	-2.051277	-3.278031
H	2.971272	-1.775183	-2.987162
C	-6.323431	0.710922	-1.686384
H	-4.334212	-0.084796	-1.904391
H	-4.890070	0.862417	-3.274050
C	-6.462546	0.551173	-0.178288
H	-6.801117	-0.141530	-2.181766
H	-6.880026	1.599035	-2.009817
H	-7.511433	0.449500	0.112207
H	-6.058359	1.418565	0.354472
H	-5.929278	-0.341302	0.166076
H	2.272928	-0.996300	-0.559918
H	1.167175	1.027709	-1.026859
H	4.341466	0.177676	-2.673751
H	4.844754	-1.572881	-1.204653
C	-0.700274	3.980188	0.337272
H	0.695868	5.203482	1.428986
H	-0.003880	3.902311	2.365449
C	-1.979700	4.708024	0.724313
H	-2.770165	4.569024	-0.018791
H	-1.807147	5.783734	0.825188
H	-2.361093	4.353736	1.688927
H	-0.903261	2.910180	0.201296
H	-0.355007	4.350941	-0.634664

conf_54 9.8 kJ mol^{-1}

C	1.607342	1.931203	3.231984
C	0.697820	1.320992	2.211300
O	0.984062	1.117795	1.051106
Ag	2.473471	-0.519912	-0.197772
C	1.635598	-2.541642	0.437668
C	0.605218	-2.072548	1.214082
O	0.627549	-2.064376	2.542058
C	-0.707870	-1.804518	3.017346
H	-0.637964	-1.161442	3.891840
H	-1.171544	-2.757867	3.276911
C	-1.369938	-1.143017	1.813836
H	-2.367415	-1.535013	1.620538
C	-1.453689	0.373366	1.861975
H	-1.375721	0.785260	0.854794
H	-2.414586	0.655618	2.293024

O -0.486177 0.972137 2.728406
O -0.543715 -1.615237 0.728259
C 2.708638 -3.418266 1.059345
H 1.323015 -2.762212 -0.583787
H 1.028773 2.191723 4.121198
H 2.291490 1.123304 3.523532
C 2.410025 3.125299 2.715434
H 6.724553 -0.350765 0.113481
C 5.395375 -0.064482 -1.554379
C 6.174947 -0.972953 -0.602432
C 5.329480 -1.984763 0.165298
C 4.527909 -2.930822 -0.720152
H 6.933975 -1.505403 -1.185703
C 3.715220 -3.967790 0.049358
H 2.228130 -4.271670 1.553519
H 3.228905 -2.876516 1.855206
H 4.402829 -4.627364 0.590529
H 3.185854 -4.606795 -0.667180
H 3.857177 -2.356898 -1.377212
H 5.212987 -3.454721 -1.397132
H 4.654677 -1.446447 0.849017
H 5.986802 -2.572676 0.816529
H -0.254335 3.651584 1.887294
H 1.464052 3.466264 0.041892
H 1.588314 5.218124 0.012968
C 0.896367 4.389368 0.206627
C 0.455224 4.458564 1.663364
H -0.109838 5.385642 1.816855
C -1.177183 3.230545 -0.752042
C 1.613183 4.428120 2.658259
C -0.243884 4.443009 -0.803977
H -0.833344 5.356422 -0.661032
H 0.179087 4.515902 -1.813554
H 1.230801 4.643892 3.663215
H 2.305101 5.243958 2.418437
H 2.828361 2.878582 1.733745
H 3.262946 3.267835 3.385154
H 1.193259 1.312229 -4.264264
H -0.889450 0.002516 -4.240301
H 3.301531 0.290819 -3.368206
C 4.350311 0.780598 -0.887227
H 1.162010 -0.937450 -3.083858
C 3.243406 1.313407 -1.478533
H -0.921845 -2.282905 -3.268990
C 1.347813 1.200690 -3.185688
C 2.852905 1.183891 -2.920278
C -2.945103 -1.565339 -3.178930
C -0.832613 -0.139477 -3.153205
C 0.623214 -0.052677 -2.715123
C -1.478201 -1.468183 -2.784767
H 0.901674 2.088500 -2.720680
H -1.402495 0.685467 -2.708585
H 3.311110 2.038651 -3.437691
H 0.644499 -0.107402 -1.612843
H -1.379730 -1.634525 -1.704283
H -6.600609 -0.004031 0.282635
H -6.140275 2.515990 0.139281
C -5.662914 -0.243695 -0.235877
H -4.118147 -1.741461 -0.123755
H -4.357748 1.052026 0.905318
C -5.029990 -1.617378 1.830400
C -5.346420 2.249857 -0.567841
H -4.918947 4.345333 -0.777800
C -5.111803 -1.556861 0.310158
C -4.707686 0.938702 -0.129775
C -3.458858 3.281227 -1.871454
C -4.353716 3.410208 -0.679852
H -5.930151 -0.374864 -1.290997
C -2.128044 3.201134 -1.904951
H -5.839070 2.107331 -1.538347
H -5.741355 -2.385037 -0.035926
H -3.816802 0.741615 -0.742524
H -3.776960 3.496972 0.247412
H -1.721838 3.216254 0.196707
H -0.557897 2.321754 -0.771617
H 4.956533 -0.645192 -2.370906
H 6.105965 0.622704 -2.033813
C -3.549247 -2.935994 -2.903596
H -3.052829 -1.332318 -4.246483
H -3.519022 -0.795635 -2.644988
C -5.022693 -3.025117 -3.270318
H -3.417081 -3.184657 -1.842462
H -2.985126 -3.693667 -3.461758
H -5.429109 -4.019348 -3.066503
H -5.177268 -2.815746 -4.333394
H -5.616237 -2.300637 -2.703704
H 4.602502 1.146062 0.110359
H 2.681476 2.052119 -0.906246
H -3.982264 3.254346 -2.827339
H -1.657050 3.134143 -2.885860
C -4.533275 -2.958177 2.358073
H -6.020782 -1.411454 2.254563
H -4.384832 -0.811860 2.210527
C -4.325316 -2.967417 3.865082
H -3.967387 -3.937294 4.220963
H -5.258623 -2.747471 4.391549
H -3.599354 -2.205546 4.175092
H -3.599359 -3.236821 1.847585
H -5.250178 -3.738912 2.079543

[PC(18:1(6*Z*)/18:1(6*Z*)) + Ag − 183]$^+$

Figure S7

conf_0 0.0 kJ mol^{-1}

C 2.202028 0.855596 -1.405407
C 1.650904 0.055119 -0.267400
O 0.681372 -0.671176 -0.324119
Ag -1.831405 -0.803612 -0.367926
C -2.290548 0.129615 1.860011
C -1.071222 -0.031236 2.436558
O -0.792068 -0.968057 3.351745
C 0.461307 -0.636740 3.965098
H 0.992832 -1.564930 4.176675
H 0.271553 -0.094317 4.894092
C 1.121560 0.230111 2.902618

H 1.664371 1.084493 3.307578
C 1.996043 -0.582742 1.969234
H 2.918761 -0.858478 2.484203
H 1.477540 -1.484710 1.637201
O 2.385635 0.191104 0.842038
O -0.014855 0.757578 2.202911
C -3.526034 -0.453611 2.515382
H -2.383829 1.061285 1.307386
H 2.378695 1.877077 -1.050491
H 3.194515 0.444313 -1.621708
C 1.293590 0.812930 -2.621817
C -0.677632 -3.930154 0.515672
C -1.824262 -3.450219 -0.322310
C -3.006949 -2.971816 0.121814
C -3.460639 -2.838534 1.542101
C -4.286830 -1.568999 1.800884
H -4.220980 0.376098 2.686196
H -3.245826 -0.821539 3.507785
H -5.153078 -1.815544 2.421909
H -4.693892 -1.197532 0.853334
H -4.078455 -3.719570 1.758466
H -2.611828 -2.892103 2.231050
H -1.978388 1.867060 -3.200128
C -1.682125 -0.231088 -2.918029
C -1.172420 1.133266 -3.270752
H -0.857443 1.119470 -4.323630
C -4.187777 0.224761 -3.112425
C -0.004242 1.585626 -2.398668
C -2.976559 -0.608049 -2.832175
H -0.311208 1.495228 -1.351620
H 0.176185 2.653106 -2.561778
H 1.086082 -0.235495 -2.857323
H 1.827484 1.221041 -3.484844
H 3.640234 -2.409573 0.360462
H 4.737586 -1.300281 -2.415408
H 3.076362 -5.062472 -1.041000
C 0.637359 -3.973840 -0.253732
H 5.746667 -3.428460 -0.460905
C 1.811729 -4.446189 0.592184
H 4.696146 -0.498648 -0.023827
C 3.630327 -3.042816 -0.536053
C 3.148756 -4.434681 -0.143473
C 6.084482 0.733024 -1.092934
C 5.462282 -1.668550 -1.673874
C 5.012255 -3.039718 -1.178398
C 5.635444 -0.626814 -0.575062
H 2.917533 -2.581565 -1.232866
H 6.409531 -1.774907 -2.215338
H 3.908638 -4.904853 0.491780
H 5.011732 -3.741995 -2.020678
H 6.364835 -0.994557 0.159090
H -1.812743 4.969112 1.063258
H -4.215356 4.512634 -0.046440
C -1.571116 3.910872 0.902611
H 0.192021 4.283527 -0.262997
H -2.150594 3.881982 -1.171101
C 0.794121 4.231935 1.810614
C -3.918771 3.458509 -0.026229
H -5.754794 3.016779 -1.087022
C -0.076943 3.752577 0.658158
C -2.412987 3.361218 -0.240475
C -4.706629 1.168962 -0.797743
C -4.715477 2.671112 -1.065585
H -1.841876 3.396418 1.835230
C -5.189836 0.304802 -1.954750
H -4.175138 3.094196 0.978618
H 0.139711 2.691949 0.478972
H -2.133372 2.311889 -0.410034
H -4.306486 2.886359 -2.061122
H -0.918042 -4.937889 0.883934
H -0.575206 -3.306515 1.412574
C 6.119163 1.807442 -0.014059
H 5.415089 1.058603 -1.902823
H 7.076777 0.641883 -1.552829
C 6.572752 3.162493 -0.535859
H 6.785163 1.482778 0.795172
H 5.120277 1.896758 0.434629
H 5.905733 3.527467 -1.324206
H 7.579636 3.104005 -0.960429
H 6.593657 3.914905 0.257492
C 2.279173 3.927637 1.625554
H 0.442100 3.759169 2.736845
H 0.657685 5.311857 1.952152
C 2.914696 4.626271 0.430382
H 2.463956 4.311730 -0.516367
H 3.985186 4.414281 0.369691
H 2.795138 5.712050 0.501443
H 2.414403 2.842288 1.523306
H 2.818463 4.217928 2.534492
H 0.839074 -2.977557 -0.656835
H 0.526114 -4.642794 -1.117078
H 1.606051 -5.459322 0.958057
H 1.891794 -3.816502 1.490788
H -5.315827 0.970077 0.091492
H -3.687009 0.852200 -0.533234
H -1.720240 -3.603500 -1.397449
H -3.781951 -2.787725 -0.623566
H -4.687321 -0.235818 -3.974056
H -3.904023 1.229832 -3.432027
H -6.137668 0.698944 -2.336520
H -5.405430 -0.710201 -1.598047
H -3.176343 -1.667464 -2.665623
H -0.929443 -1.018290 -2.842050

conf_20 3.8 kJ mol^{-1}

C 2.199915 1.261368 -0.552980
C 1.503393 0.304539 0.363531
O 0.646195 -0.487186 0.033012
Ag -1.780903 -0.835508 -0.592254
C -2.805416 -0.088117 1.505754
C -1.739088 -0.207864 2.339829
O -1.599407 -1.189694 3.239122
C -0.546671 -0.826291 4.142734
H -0.000785 -1.731214 4.411014
H -0.983893 -0.374766 5.036112
C 0.260935 0.165690 3.317833

H 0.619901 1.021560 3.889897
C 1.394151 -0.504369 2.566726
H 2.192855 -0.758598 3.266482
H 1.046282 -1.408822 2.063236
O 1.963819 0.387351 1.617119
O -0.727496 0.666183 2.405097
C -4.107585 -0.790654 1.835864
H -2.846557 0.873007 0.998845
H 2.157566 2.257480 -0.097754
H 3.259059 0.980278 -0.554641
C 1.603474 1.241721 -1.950002
C -0.617205 -3.932984 0.337846
C -1.596526 -3.473749 -0.699651
C -2.879981 -3.104152 -0.499388
C -3.638962 -3.099593 0.790675
C -4.602670 -1.910453 0.922656
H -4.885021 -0.019784 1.879384
H -4.028657 -1.196564 2.849660
H -5.557923 -2.255914 1.328885
H -4.827746 -1.511001 -0.072695
H -4.214847 -4.033570 0.819284
H -2.958427 -3.141730 1.646946
H -1.553193 2.009378 -3.214040
C -1.088479 -0.065292 -2.989954
C -0.672981 1.367307 -3.135590
H -0.127854 1.471743 -4.084241
C -3.506855 0.147131 -3.782757
C 0.206933 1.858778 -1.989267
C -2.311479 -0.577519 -3.248915
H -0.315226 1.648464 -1.050033
H 0.293044 2.948957 -2.041582
H 1.579688 0.204289 -2.297930
H 2.267712 1.778603 -2.633320
H 3.463646 -1.994215 1.110761
H 5.217195 -0.324697 0.406766
H 3.473287 -4.634137 -0.431285
C 0.827467 -3.797079 -0.128130
H 5.783611 -2.763988 0.678628
C 1.846184 -4.222443 0.919763
H 7.583919 -1.051009 0.067045
C 3.687589 -2.592784 0.216142
C 3.295352 -4.042133 0.475766
C 7.284729 0.737018 -1.092924
C 5.499324 -0.952427 -0.451130
C 5.153732 -2.402899 -0.144437
C 6.966809 -0.717860 -0.777537
H 3.071046 -2.178651 -0.591859
H 4.886490 -0.611256 -1.298472
H 3.958780 -4.459151 1.242476
H 5.400760 -3.027692 -1.012318
H 7.254587 -1.347245 -1.629721
H -2.779136 4.795499 1.067966
H -4.767447 4.198226 -0.655030
C -2.391618 3.779888 0.918461
H -0.443065 4.409306 0.276185
H -2.430433 3.866549 -1.232966
C -0.382449 4.228027 2.428170
C -4.366961 3.180257 -0.602078
H -5.818507 2.628666 -2.112282
C -0.876233 3.777380 1.060919
C -2.854117 3.247887 -0.430614
C -4.675184 0.879555 -1.632912
C -4.785385 2.387407 -1.839173
H -2.831683 3.170888 1.720677
C -4.760422 0.047759 -2.905585
H -4.821128 2.725143 0.289356
H -0.506531 2.760371 0.878573
H -2.422841 2.248127 -0.581186
H -4.167617 2.705898 -2.688645
H -0.829295 -4.985804 0.573015
H -0.770979 -3.384288 1.275446
C 8.752386 0.984969 -1.414418
H 6.990734 1.366401 -0.241213
H 6.669144 1.067940 -1.941338
C 9.054474 2.442632 -1.728246
H 9.045708 0.355988 -2.264063
H 9.366504 0.654953 -0.567364
H 10.113151 2.593714 -1.954675
H 8.801145 3.089785 -0.882083
H 8.478372 2.788400 -2.592870
C 1.125581 4.079195 2.614964
H -0.900092 3.641959 3.198638
H -0.669970 5.273517 2.599061
C 1.957793 4.946568 1.679216
H 3.026296 4.841303 1.885639
H 1.701691 6.004584 1.794106
H 1.798771 4.686084 0.628059
H 1.403035 3.025577 2.473356
H 1.382587 4.326441 3.651333
H 1.001138 -2.757316 -0.418927
H 0.970564 -4.401871 -1.033218
H 1.674357 -5.272424 1.185431
H 1.679607 -3.650598 1.844628
H -5.460116 0.566593 -0.934844
H -3.724512 0.648065 -1.129756
H -1.254028 -3.543648 -1.733173
H -3.487415 -2.915622 -1.385764
H -3.729258 -0.301095 -4.759307
H -3.270490 1.194928 -3.980427
H -5.627247 0.366793 -3.494112
H -4.937300 -1.005147 -2.652469
H -2.419970 -1.661313 -3.189485
H -0.289215 -0.775119 -2.767620

conf_23 7.3 kJ mol^{-1}

C 0.579603 2.619044 -1.678795
C -0.154545 1.440783 -1.141312
O 0.291875 0.661282 -0.317844
Ag 1.788419 -1.098310 -0.079675
C 1.689851 -2.129940 -2.193318
C 0.344674 -1.950038 -2.324217
O -0.585029 -2.827538 -1.935930
C -1.868507 -2.370881 -2.393328
H -2.597260 -2.549465 -1.602177
H -2.141769 -2.933288 -3.288362
C -1.619163 -0.895232 -2.678685

H -2.082668 -0.548140 -3.602022
C -1.980012 0.011771 -1.511939
H -3.056561 0.186717 -1.491588
H -1.667517 -0.421699 -0.561760
O -1.354880 1.277462 -1.701575
O -0.199857 -0.870576 -2.898813
C 2.302944 -3.499431 -2.007346
H 2.279641 -1.379503 -2.716643
H -0.107101 3.456182 -1.828025
H 1.339698 2.896924 -0.947096
C 1.225386 2.213923 -3.014715
C 2.361486 -2.799392 2.713770
C 3.064835 -1.807862 1.837578
C 3.721505 -2.086102 0.671073
C 3.896116 -3.437933 0.043420
C 3.740544 -3.511580 -1.477993
H 2.306199 -3.995483 -2.986353
H 1.665113 -4.115835 -1.364925
H 4.203486 -4.447325 -1.803635
H 4.320335 -2.711082 -1.954676
H 4.917170 -3.756278 0.293043
H 3.233157 -4.172310 0.509397
H 4.061586 4.220711 -3.234424
C 4.145743 2.293199 -2.340854
C 3.394002 3.539916 -2.691599
H 3.093278 4.079826 -1.789419
C 4.438886 2.673719 0.149269
C 2.175218 3.263539 -3.576502
C 4.582647 1.922517 -1.134923
H 2.517596 2.912351 -4.556767
H 1.637439 4.200751 -3.758048
H 0.434233 2.001504 -3.739106
H 1.776414 1.278881 -2.865785
H -2.444414 -2.243766 1.150238
H -4.557159 0.116576 1.110497
H -1.350747 -1.016986 3.717342
C 1.064123 -2.304728 3.350203
H -4.211794 -2.352598 2.886629
C -0.048750 -2.048847 2.345599
H -4.209368 -2.172678 -0.069779
C -2.477072 -1.485881 1.944619
C -1.415251 -1.825511 2.978439
C -5.915471 -1.127769 -0.843965
C -4.904475 -0.853548 1.488943
C -3.889835 -1.376000 2.502652
C -5.164385 -1.786363 0.309540
H -2.212061 -0.528511 1.477475
H -5.849399 -0.650664 2.002830
H -1.707882 -2.726796 3.531709
H -3.873720 -0.700477 3.366413
H -5.720231 -2.667079 0.655829
H -0.869336 1.241989 1.858501
H 1.410805 0.732431 2.506567
C -0.922922 2.182140 2.421256
H -1.191024 3.476313 0.725524
H 0.933498 3.036876 1.780479
C -3.108945 2.640875 1.213632
C 1.375494 1.534023 3.256224
H 2.745109 2.620277 4.507223
C -1.749221 3.182934 1.626464
C 0.496166 2.649690 2.709833
C 3.562970 2.678155 2.527555
C 2.792514 1.960713 3.632970
H -1.434925 1.944219 3.362682
C 3.716790 1.882564 1.237980
H 0.893104 1.094190 4.138229
H -1.877978 4.105725 2.206148
H 0.481109 3.493886 3.411177
H 3.359423 1.077916 3.958418
H 3.068345 -3.059902 3.513706
H 2.166081 -3.728559 2.170282
C -7.294298 -0.589615 -0.482647
H -6.020887 -1.846979 -1.666824
H -5.312621 -0.296669 -1.241449
C -8.012624 0.023275 -1.675252
H -7.201000 0.161941 0.308971
H -7.897052 -1.404368 -0.062166
H -9.000657 0.399328 -1.398267
H -8.149701 -0.710324 -2.476292
H -7.443774 0.863358 -2.088071
C -3.921360 3.593644 0.348742
H -3.686242 2.376291 2.109567
H -2.962910 1.699073 0.666660
C -5.254311 3.005669 -0.090695
H -5.872760 2.737558 0.771850
H -5.110551 2.095929 -0.684293
H -5.825002 3.710091 -0.701250
H -3.330962 3.869380 -0.534735
H -4.093199 4.525569 0.900436
H 1.254406 -1.395490 3.931942
H 0.729206 -3.062253 4.067023
H -0.113328 -2.895437 1.646398
H 0.192142 -1.156728 1.743034
H 4.558345 2.943798 2.902332
H 3.069787 3.630714 2.298520
H 3.212756 -0.818873 2.270487
H 4.364278 -1.300311 0.267045
H 3.917382 3.622434 -0.009882
H 5.439161 2.941489 0.515401
H 2.730763 1.579940 0.860401
H 4.274104 0.957303 1.440987
H 5.136199 0.984917 -1.067345
H 4.367543 1.635385 -3.182256

[PC(16:0/18:1) + Ag − 183 − AgH]$^+$

Figure S9

trans_1 0.0 kJ mol^{-1}

C -2.330450 -1.721415 2.931170
C -1.063111 -1.959441 2.171850
O -0.786020 -1.458497 1.110255
O -0.218188 -2.811902 2.789253
C 0.890229 -3.286294 2.031137
C 1.982358 -2.273453 1.689397
O 1.821019 -1.772673 0.328468

C	1.508463	-0.519604	0.373162
C	1.120214	0.233152	-0.759278
C	1.065239	-0.356769	-1.971108
C	0.719327	0.297963	-3.248285
C	0.068008	1.672723	-3.192869
C	-1.367548	1.631304	-2.683233
C	-2.083529	2.969583	-2.790947
C	-3.539503	2.903942	-2.324140
C	-3.667870	2.580271	-0.868735
C	-4.398772	1.619587	-0.300142
C	-5.283949	0.614513	-0.967814
C	-4.853529	-0.831845	-0.705379
C	-3.499672	-1.166143	-1.312219
C	-3.059520	-2.605188	-1.094881
C	-1.675666	-2.886197	-1.662744
C	-1.136548	-4.267033	-1.321462
C	0.295998	-4.491217	-1.787505
C	0.848051	-5.857914	-1.409644
H	1.878143	-5.988534	-1.751951
H	0.248700	-6.659430	-1.851000
H	0.835298	-6.007861	-0.323850
H	0.344475	-4.364380	-2.876236
H	0.937606	-3.705273	-1.364289
H	-1.194571	-4.416227	-0.232641
H	-1.785364	-5.038889	-1.754088
H	-0.986358	-2.128049	-1.270481
H	-1.694661	-2.760271	-2.753981
H	-3.791057	-3.294061	-1.536060
H	-3.053379	-2.820767	-0.017894
H	-2.741250	-0.495207	-0.891698
H	-3.529557	-0.954743	-2.390088
H	-5.616285	-1.509166	-1.106842
H	-4.824473	-1.012345	0.378137
H	-5.329017	0.787477	-2.047434
H	-6.306164	0.750987	-0.593496
H	-4.371756	1.552624	0.787965
H	-3.098223	3.232888	-0.207144
H	-4.085882	2.180289	-2.936699
H	-4.006684	3.879379	-2.510055
H	-1.546446	3.726312	-2.204868
H	-2.056507	3.313495	-3.831541
H	-1.398211	1.285074	-1.643195
H	-1.926663	0.881532	-3.258199
H	0.672932	2.360538	-2.588632
H	0.078155	2.087774	-4.206103
H	0.093826	-0.402856	-3.818241
H	1.662712	0.331229	-3.819297
H	1.332840	-1.410251	-2.032834
H	0.874643	1.272280	-0.581229
O	1.606187	0.025575	1.540565
C	2.025482	-0.975386	2.499182
H	1.321884	-0.952944	3.329949
H	3.026794	-0.704403	2.832732
H	2.944444	-2.786644	1.683933
H	0.544737	-3.741365	1.098568
H	1.325772	-4.061662	2.661752
C	-2.758264	-0.261795	2.820672
C	-1.904663	0.657198	3.694017
C	-1.887168	2.112879	3.236490
C	-0.995460	2.333961	2.020603
C	-0.863026	3.794000	1.609590
C	0.110842	4.016474	0.455420
C	1.571485	3.773648	0.820684
C	2.538788	4.046971	-0.324887
C	4.011427	3.928577	0.065703
C	4.472080	2.527283	0.463341
C	4.499700	1.533191	-0.690677
C	4.900438	0.126761	-0.268373
C	4.905355	-0.881126	-1.409333
C	5.234701	-2.297127	-0.962551
H	6.214381	-2.341125	-0.477439
H	5.251002	-2.994264	-1.804148
H	4.493664	-2.668260	-0.245019
H	3.927521	-0.868191	-1.909287
H	5.628915	-0.557148	-2.166240
H	4.220672	-0.223094	0.524721
H	5.893478	0.152531	0.198222
H	3.517782	1.494693	-1.178514
H	5.198431	1.891000	-1.457580
H	3.831562	2.138690	1.266856
H	5.478307	2.594145	0.893473
H	4.633523	4.280317	-0.766082
H	4.204533	4.616178	0.897654
H	2.316346	3.377415	-1.167507
H	2.358410	5.059919	-0.703460
H	1.701608	2.746428	1.181065
H	1.834805	4.418889	1.669524
H	-0.176280	3.370518	-0.387378
H	0.008902	5.043980	0.086839
H	-0.536262	4.387947	2.473207
H	-1.850398	4.184418	1.336768
H	-0.002698	1.931798	2.253895
H	-1.369964	1.752638	1.166799
H	-2.909932	2.449785	3.023136
H	-1.526352	2.744022	4.056720
H	-0.865290	0.294492	3.705215
H	-2.251983	0.587359	4.730498
H	-2.692886	0.033722	1.769820
H	-3.809774	-0.170995	3.107770
H	-3.077684	-2.383475	2.474951
H	-2.208378	-2.047144	3.966952

trans_2 4.8 kJ mol^{-1}

C	2.599163	-0.530080	2.811895
C	1.588471	0.442998	2.293596
O	1.240318	0.531255	1.141744
O	1.066200	1.237845	3.251472
C	0.275337	2.340301	2.820175
C	-1.085930	2.016753	2.202785
C	-1.653616	0.612953	2.425953
O	-1.543975	0.000397	1.117016
C	-1.166482	0.896257	0.264505
C	-0.952639	0.633008	-1.109537
C	-1.019366	-0.622156	-1.592874
C	-0.744354	-0.994026	-2.993015
C	0.348947	-2.067285	-3.108390

C	1.713183	-1.628968	-2.592676
C	2.770952	-2.693672	-2.852786
C	4.171356	-2.323179	-2.368249
C	4.278001	-2.248713	-0.876587
C	4.959513	-1.359904	-0.152704
C	5.776241	-0.206083	-0.642449
C	5.221906	1.150039	-0.197080
C	3.892550	1.492613	-0.852646
C	3.314202	2.825584	-0.400717
C	1.992231	3.152761	-1.079080
C	1.370551	4.468325	-0.634871
C	0.060172	4.795354	-1.338995
C	-0.559614	6.103159	-0.870368
H	0.124757	6.941851	-1.029592
H	-0.794484	6.070662	0.199146
H	-1.485829	6.327371	-1.405798
H	0.237772	4.838825	-2.420647
H	-0.653756	3.976505	-1.178913
H	2.083859	5.284936	-0.804441
H	1.202569	4.450278	0.452404
H	2.142957	3.176162	-2.166773
H	1.293902	2.330271	-0.883116
H	4.035852	3.629530	-0.594620
H	3.171435	2.808826	0.689632
H	4.029709	1.512461	-1.942706
H	3.167715	0.695708	-0.648587
H	5.956833	1.930362	-0.426256
H	5.107774	1.156217	0.896412
H	5.862528	-0.221716	-1.733236
H	6.796067	-0.317133	-0.254174
H	4.947716	-1.484520	0.930655
H	3.762337	-3.046805	-0.340292
H	4.484853	-1.381715	-2.830236
H	4.870210	-3.088673	-2.730678
H	2.809750	-2.899557	-3.928911
H	2.463011	-3.633988	-2.375609
H	1.663168	-1.403610	-1.518701
H	2.010597	-0.691525	-3.081627
H	0.027162	-2.971406	-2.578367
H	0.425797	-2.345516	-4.164607
H	-1.676173	-1.407135	-3.406459
H	-0.486317	-0.109738	-3.583091
H	-1.275267	-1.430366	-0.909183
H	-0.672948	1.484480	-1.717434
O	-1.011796	2.081384	0.747691
H	-2.709131	0.609798	2.696419
H	-1.073124	0.011097	3.123217
H	-1.794415	2.797216	2.481538
H	0.115404	2.920437	3.729021
H	0.834400	2.948935	2.103545
C	2.288646	-1.943056	2.321039
C	1.113569	-2.569125	3.065707
C	0.498380	-3.777323	2.366937
C	-0.352081	-3.400247	1.158736
C	-1.136891	-4.569719	0.575979
C	-2.142081	-4.174420	-0.503513
C	-3.285997	-3.299942	-0.000999
C	-4.286421	-2.922495	-1.086553
C	-5.531172	-2.210627	-0.559066
C	-5.285962	-0.863329	0.117762
C	-4.784423	0.226173	-0.820987
C	-4.587325	1.570539	-0.134264
C	-4.113405	2.679067	-1.064814
C	-3.871519	4.000481	-0.351827
H	-4.777477	4.347620	0.154115
H	-3.559905	4.783279	-1.047364
H	-3.083845	3.907364	0.404448
H	-4.860540	2.819787	-1.854400
H	-3.197732	2.360861	-1.580686
H	-3.871665	1.456906	0.695459
H	-5.527518	1.877190	0.341696
H	-5.500159	0.347892	-1.644176
H	-3.841727	-0.081770	-1.290620
H	-4.578555	-0.986182	0.949572
H	-6.224154	-0.526754	0.574691
H	-6.033901	-2.876725	0.152281
H	-6.237257	-2.064844	-1.385325
H	-3.790119	-2.305165	-1.848395
H	-4.603336	-3.832773	-1.608932
H	-2.889773	-2.388398	0.462753
H	-3.811993	-3.834592	0.801572
H	-1.620358	-3.666650	-1.327131
H	-2.565871	-5.082813	-0.946747
H	-1.673996	-5.077983	1.387426
H	-0.436531	-5.308676	0.169600
H	0.277689	-2.955732	0.375499
H	-1.049565	-2.614044	1.472477
H	1.287424	-4.480552	2.071779
H	-0.134505	-4.316492	3.081138
H	0.321390	-1.817812	3.206243
H	1.439535	-2.843274	4.075127
H	2.096474	-1.893431	1.245817
H	3.176820	-2.569116	2.448908
H	2.651931	-0.472005	3.900991
H	3.561974	-0.192800	2.410743

trans_3 5.9 kJ mol^{-1}

C	-0.936544	-0.194623	0.967566
C	-0.883924	-0.596233	-0.384438
C	0.001673	-0.027584	-1.230064
C	0.234997	-0.476082	-2.613735
H	0.639165	0.767302	-0.851163
H	-1.545870	-1.401270	-0.673535
O	-0.256425	0.795566	1.440891
C	-0.390557	0.827802	2.893249
C	-1.547121	-0.149973	3.118722
O	-1.662171	-0.821654	1.836230
H	-2.500197	0.341470	3.310102
H	-1.332972	-0.912066	3.864964
C	0.927726	0.439529	3.527697
O	1.123530	-0.967255	3.511782
C	1.882880	-1.493232	2.508677
O	2.295550	-0.824532	1.599864
H	0.908974	0.732135	4.579932
H	1.746275	0.953158	3.017542
H	-0.648785	1.856338	3.147384

C	2.109914	-2.960483	2.715306
H	0.403048	-2.616749	0.478186
H	2.665929	-3.462654	0.693124
C	1.924925	-3.773978	1.436912
C	0.523881	-3.639150	0.855027
H	-1.159658	-5.069979	-1.843570
H	1.091548	-4.638173	-0.961689
C	0.235072	-4.619024	-0.275868
H	-0.218291	-3.769297	1.652947
C	-1.026390	-4.299082	-1.075313
H	-4.453393	-4.244977	-0.453928
H	2.135624	-4.822427	1.671700
H	-2.382075	-5.110246	0.374085
C	-2.307242	-4.209966	-0.248435
C	-3.576353	-4.095751	-1.094882
H	-0.866344	-3.363345	-1.627287
H	-3.602465	-1.505745	-0.103985
H	-2.799410	-2.513946	-2.353689
H	0.159611	-5.632088	0.138327
C	-3.742605	-2.779123	-1.854436
C	-4.221348	-1.604273	-1.004971
H	-3.225009	-0.088251	-2.169261
H	-3.583843	-4.925610	-1.811069
H	-5.751602	0.745007	-0.655703
H	-2.250833	-3.371053	0.458356
C	-4.236345	-0.296259	-1.789329
C	-4.734185	0.926455	-1.024289
H	-3.869089	0.578107	0.921588
H	-5.232740	-1.819320	-0.636888
H	-4.460836	-2.923841	-2.669213
H	-5.238505	2.682296	1.091809
C	-3.839774	1.346270	0.136448
C	-4.200403	2.696601	0.737136
H	-3.370323	2.380916	2.694095
H	-4.860392	-0.435720	-2.679859
H	-4.811564	1.767547	-1.723709
H	-2.906062	4.774172	3.238015
C	-3.279595	3.112491	1.877246
C	-3.575280	4.503963	2.416406
H	-2.802817	1.396917	-0.224280
H	-4.159197	3.459789	-0.050423
H	-3.458797	5.257951	1.632310
H	-2.239332	3.070298	1.523906
H	-4.601768	4.571487	2.788353
H	3.137665	-3.059611	3.084910
H	1.449350	-3.314277	3.509962
H	5.696124	2.525065	-1.392519
H	3.757320	3.054794	1.743927
H	1.576199	4.160956	1.170540
H	5.857656	0.584102	-2.677873
C	5.373464	1.591579	-0.929932
H	2.118594	4.765256	-0.378158
C	1.658992	3.895407	0.108099
H	4.396400	3.815815	0.301621
C	3.927869	2.894610	0.671533
H	-0.373907	4.539155	-0.190089
C	5.472767	0.481248	-1.663036
C	0.256372	3.681225	-0.455076
C	4.897797	1.726075	0.484324
C	2.589568	2.694989	-0.027504
H	3.821796	-2.547862	-1.811852
H	0.892345	2.699820	-2.280199
H	5.770325	1.895195	1.129456
C	5.118792	-0.916175	-1.259835
H	-0.200120	2.815116	0.042506
H	2.107970	1.800971	0.394010
C	3.991419	-1.506353	-2.112648
C	0.188373	3.481677	-1.964484
H	6.008389	-1.549479	-1.362305
H	4.295158	-1.534187	-3.166791
H	4.428137	0.800387	0.832044
H	2.775358	2.475049	-1.085307
H	0.526595	4.397053	-2.466217
H	4.826318	-0.956319	-0.205990
C	2.702470	-0.713860	-1.965560
C	-1.209824	3.122268	-2.450471
H	2.495720	-0.601938	-0.893997
H	-1.523241	2.177898	-1.981582
C	1.509662	-1.354953	-2.657541
H	-0.623759	2.248603	-4.355268
H	-1.921136	3.876683	-2.091290
C	-1.315877	3.001477	-3.962625
H	2.858583	0.300498	-2.353680
H	-1.070993	3.949701	-4.450076
H	-2.324740	2.718733	-4.274951
H	0.394888	0.402855	-3.247448
H	-0.622915	-1.033314	-2.999031
H	1.734304	-1.556643	-3.709867
H	1.282255	-2.320271	-2.191329

cis_1 13.8 kJ mol^{-1}

C	5.067386	-1.504523	-0.465184
C	4.414230	-0.335041	-1.136729
O	3.591336	-0.399356	-2.009579
O	4.850290	0.861114	-0.641027
C	4.435184	2.034710	-1.326281
C	3.207743	2.665991	-0.706230
H	3.028106	3.648768	-1.142400
C	3.165546	2.673976	0.822477
H	4.117684	2.420894	1.285245
H	2.750393	3.589225	1.244224
O	2.240379	1.599948	1.126560
C	1.653881	1.224777	0.032183
O	2.031878	1.859806	-1.027588
C	0.701714	0.184606	0.055052
C	0.116039	-0.348879	-1.044618
C	0.240668	0.051412	-2.462420
H	0.008501	-0.817397	-3.083965
C	-0.776388	1.175423	-2.767814
C	-2.199788	0.805247	-2.379275
C	-3.207397	1.921668	-2.596828
C	-4.604107	1.540470	-2.097255
C	-4.625421	1.263221	-0.625817
C	-4.999536	0.140033	-0.010064
C	-5.514279	-1.123748	-0.624249
C	-4.794867	-2.375415	-0.118106

C -3.331180 -2.452869 -0.529861
C -2.653373 -3.736814 -0.066194
C -1.151660 -3.793156 -0.326116
C -0.744091 -3.743351 -1.793400
C 0.765747 -3.710379 -1.990418
C 1.188738 -3.727473 -3.450852
H 0.854179 -4.644239 -3.945359
H 2.275130 -3.668000 -3.556177
H 0.758129 -2.885106 -4.002915
H 1.215560 -4.562175 -1.465311
H 1.175428 -2.814696 -1.500689
H -1.190673 -2.868732 -2.286944
H -1.162429 -4.614085 -2.314145
H -0.664627 -2.968833 0.215758
H -0.742052 -4.706908 0.120858
H -3.138203 -4.595413 -0.548201
H -2.827299 -3.858314 1.010068
H -2.799230 -1.583983 -0.117463
H -3.266075 -2.361722 -1.621422
H -5.320111 -3.261973 -0.491955
H -4.866321 -2.418494 0.976737
H -5.455894 -1.084181 -1.716534
H -6.580383 -1.215341 -0.378982
H -4.955098 0.128231 1.079540
H -4.294433 2.092760 0.001219
H -4.970241 0.678453 -2.662749
H -5.293196 2.365592 -2.314708
H -3.257106 2.185051 -3.659648
H -2.867508 2.822651 -2.068916
H -2.513880 -0.088246 -2.935292
H -2.235817 0.523116 -1.319215
H -0.713061 1.403061 -3.836766
H -0.471452 2.082145 -2.233676
H 1.254063 0.379263 -2.695830
H -0.583937 -1.153927 -0.835302
H 0.470054 -0.192626 1.043302
H 4.243157 1.817823 -2.379444
H 5.263086 2.743264 -1.244410
C 4.034967 -2.392001 0.236420
C 3.510612 -1.768469 1.522528
C 2.316497 -2.494266 2.125049
C 1.894654 -1.901047 3.465470
C 0.591855 -2.468390 4.026310
C -0.672265 -2.019088 3.295663
C -0.993378 -0.537912 3.454757
C -2.256548 -0.116261 2.713407
C -2.551183 1.379163 2.775429
C -1.570864 2.226656 1.974591
C -1.895518 3.714737 1.994043
C -0.822249 4.589154 1.353851
C -0.575405 4.298442 -0.120891
C 0.469716 5.211557 -0.744154
H 0.144333 6.255383 -0.724765
H 1.415941 5.171241 -0.189549
H 0.670237 4.951012 -1.788116
H -1.519524 4.398029 -0.670395
H -0.275891 3.250842 -0.245842
H -1.098141 5.644236 1.465722
H 0.118890 4.465982 1.911076
H -2.855603 3.885459 1.489519
H -2.040079 4.034928 3.032408
H -0.552525 2.085996 2.364119
H -1.564799 1.859306 0.940001
H -2.554174 1.710859 3.821755
H -3.563047 1.564290 2.396634
H -3.106319 -0.674582 3.123797
H -2.186690 -0.420560 1.659488
H -1.106396 -0.304853 4.521577
H -0.142999 0.068118 3.116882
H -1.523055 -2.604145 3.664310
H -0.595733 -2.263894 2.227999
H 0.503620 -2.185098 5.081768
H 0.647987 -3.563439 4.011983
H 2.700377 -2.072677 4.188616
H 1.816052 -0.809115 3.372670
H 2.556377 -3.556626 2.258832
H 1.484010 -2.467781 1.410056
H 4.323410 -1.725110 2.259044
H 3.228794 -0.723287 1.340683
H 4.497084 -3.358833 0.457089
H 3.212367 -2.592391 -0.458171
H 5.832856 -1.157477 0.231660
H 5.563057 -2.066772 -1.263161

cis_2 29.0 kJ mol^{-1}

C -5.552516 -0.347764 -0.446894
C -4.102338 0.003473 -0.517853
O -3.435643 0.506950 0.354746
O -3.565132 -0.292616 -1.720081
C -2.244056 0.177447 -1.926019
C -1.228969 -0.477960 -0.998781
C -0.788218 0.290332 0.245717
O 0.675011 0.281283 0.141262
C 1.009841 -0.167605 -1.019562
C 2.327432 -0.203048 -1.531858
C 3.447530 0.134961 -0.846700
C 3.612983 0.622138 0.530716
C 4.944732 0.196164 1.154150
C 5.151488 0.678416 2.590301
C 5.717067 2.085218 2.789832
C 4.803558 3.269661 2.457432
C 4.785459 3.687732 1.019497
C 3.740827 4.151473 0.326338
C 2.335782 4.308393 0.818228
C 1.287153 4.015340 -0.251187
C -0.135901 4.126582 0.275816
C -1.206553 3.962605 -0.794415
C -2.618863 3.904077 -0.226854
C -3.708063 3.789032 -1.283804
C -5.105208 3.643914 -0.695000
C -6.179059 3.446801 -1.754506
H -6.223400 4.300554 -2.437470
H -5.979777 2.557248 -2.363398
H -7.170286 3.331595 -1.307741
H -5.110341 2.801239 0.008085
H -5.337235 4.532269 -0.095678

H	-3.676627	4.665300	-1.943956
H	-3.506419	2.926896	-1.936754
H	-2.704913	3.056824	0.467214
H	-2.797680	4.801734	0.378322
H	-1.001700	3.052954	-1.381810
H	-1.130272	4.787236	-1.513840
H	-0.287109	3.382516	1.071688
H	-0.267197	5.100493	0.763536
H	1.424245	4.706859	-1.092007
H	1.451921	3.008189	-0.660742
H	2.192905	5.339480	1.170869
H	2.158606	3.672382	1.693545
H	3.918651	4.475251	-0.699031
H	5.752284	3.661781	0.516544
H	3.790260	3.077266	2.828317
H	5.161255	4.134233	3.032899
H	6.654513	2.181686	2.226807
H	5.997963	2.166031	3.844801
H	4.209584	0.580882	3.147016
H	5.846775	-0.017716	3.070619
H	5.770060	0.547019	0.521955
H	4.994484	-0.897698	1.143640
H	2.767494	0.355214	1.167946
H	3.604572	1.726272	0.447032
H	4.371578	0.044951	-1.418520
H	2.394951	-0.553435	-2.554306
O	0.026151	-0.588894	-1.749365
H	-1.052080	-0.192793	1.181306
H	-1.098083	1.332602	0.251978
H	-1.532571	-1.501316	-0.769654
H	-2.003071	-0.100486	-2.952089
H	-2.211031	1.265282	-1.825995
C	-6.067928	-0.526790	0.976115
C	-5.364404	-1.627216	1.765595
C	-5.448508	-3.013341	1.137413
C	-4.783201	-4.101693	1.978382
C	-3.288137	-3.906386	2.223170
C	-2.432402	-3.941201	0.962875
C	-0.956112	-3.693821	1.243167
C	-0.048108	-3.907156	0.039682
C	1.408030	-3.559805	0.314552
C	2.315063	-3.704684	-0.898672
C	3.770558	-3.377861	-0.599793
C	4.652359	-3.309891	-1.837371
C	6.097100	-2.934971	-1.535138
C	6.963566	-2.842060	-2.781939
H	7.994136	-2.573192	-2.536665
H	6.579815	-2.088541	-3.478110
H	6.988918	-3.797220	-3.315114
H	6.118666	-1.976615	-0.997735
H	6.522450	-3.671968	-0.843336
H	4.229850	-2.583138	-2.547230
H	4.628226	-4.275498	-2.358265
H	3.819796	-2.416962	-0.067774
H	4.175369	-4.119294	0.100485
H	1.950684	-3.052702	-1.705566
H	2.242244	-4.725625	-1.293263
H	1.475407	-2.529111	0.696923
H	1.782382	-4.192796	1.128975
H	-0.408164	-3.320905	-0.818971
H	-0.117239	-4.953216	-0.282679
H	-0.825776	-2.671726	1.631165
H	-0.627220	-4.353101	2.056222
H	-2.796315	-3.198018	0.238901
H	-2.552745	-4.915142	0.470877
H	-3.115677	-2.962363	2.755581
H	-2.938841	-4.693840	2.901296
H	-5.292898	-4.160003	2.947413
H	-4.941034	-5.072877	1.494132
H	-5.001536	-3.005865	0.135233
H	-6.502565	-3.277731	0.988122
H	-4.317411	-1.340682	1.910091
H	-5.803039	-1.665828	2.769607
H	-5.967945	0.421790	1.512331
H	-7.140320	-0.740480	0.914918
H	-5.724351	-1.227770	-1.071637
H	-6.082172	0.474822	-0.945347

[PC(3:0/4:0) + Ag − 183 − AgH]$^+$

Figure S10

trans_initial

C	0.303194	-1.474974	0.423212
C	1.691115	-1.269728	0.612375
C	2.463546	-0.892155	-0.429888
C	3.898994	-0.588615	-0.341856
H	4.305664	-0.716179	0.661187
H	4.072464	0.439156	-0.683891
H	4.450182	-1.225252	-1.045009
H	1.988594	-0.775027	-1.402317
H	2.063004	-1.362005	1.626039
O	-0.247510	-1.566328	-0.739847
C	-1.699729	-1.479286	-0.581045
C	-1.857401	-1.766552	0.913836
O	-0.510098	-1.564184	1.423634
H	-2.138625	-2.794347	1.141747
H	-2.504362	-1.058056	1.427264
C	-2.165552	-0.111650	-1.030233
O	-1.822900	0.892686	-0.087613
C	-0.673091	1.598264	-0.310840
O	0.105483	1.293627	-1.172214
C	-0.556928	2.743277	0.644325
C	0.821358	3.377872	0.644987
H	1.095066	3.724498	-0.352865
H	0.841030	4.232796	1.322844
H	1.584378	2.668210	0.975057
H	-0.855220	2.392178	1.637366
H	-1.328213	3.466989	0.352440
H	-3.256641	-0.119134	-1.090420
H	-1.746498	0.116852	-2.012764
H	-2.123666	-2.255806	-1.217601

cis_initial

C	1.464004	0.001808	0.676537
C	1.656036	1.340449	1.092613

C 1.763385 2.388380 0.240073
C 1.717114 2.379115 -1.237189
H 2.523066 1.762947 -1.650283
H 0.777781 1.927840 -1.578724
H 1.803150 3.386717 -1.639164
H 1.882625 3.356498 0.722886
H 1.679664 1.486326 2.165507
O 1.511045 -0.391264 -0.551406
C 1.049874 -1.781515 -0.622936
C 1.124468 -2.202561 0.845618
O 1.217916 -0.929725 1.540517
H 2.016822 -2.774796 1.098248
H 0.224236 -2.699329 1.201463
C -0.332780 -1.824174 -1.236453
O -1.333864 -1.418765 -0.315688
C -1.737377 -0.114747 -0.359474
O -1.171388 0.704169 -1.031661
C -2.929426 0.106783 0.515070
C -3.268637 1.574086 0.699018
H -3.453272 2.058823 -0.261050
H -2.455677 2.110785 1.193971
H -4.164718 1.677194 1.313160
H -3.758908 -0.440501 0.050140
H -2.750663 -0.402438 1.467859
H -0.559969 -2.860485 -1.498150
H -0.357923 -1.207272 -2.137525
H 1.764147 -2.303365 -1.259696

PC(3:0/4:0) Hydride abstraction Figure S11

[Fragment + Ag]$^+$

C -1.216161 0.915358 -0.130224
C -1.993093 -0.222847 -0.277270
C -2.891330 -0.666841 0.861225
H -3.165556 -1.714144 0.711048
H -2.377928 -0.330401 -1.292374
O -0.922573 1.458798 1.038900
C -0.257784 2.718074 0.815483
C 0.185976 2.614668 -0.642858
O -0.695328 1.586370 -1.147073
C 1.650083 2.267240 -0.867521
O 2.217549 1.519447 0.216129
C 2.259172 0.192204 0.137776
O 1.798722 -0.418175 -0.811866
C 2.923684 -0.433389 1.321565
C 2.556656 -1.895770 1.503049
H 1.482248 -2.014744 1.692883
H 2.828672 -2.486180 0.625990
H 3.079293 -2.313909 2.364645
H 4.003520 -0.322203 1.158099
H 2.695918 0.167314 2.205845
H 2.230655 3.190037 -0.898390
H 1.767947 1.727275 -1.808438
H -0.044817 3.514455 -1.214487
H 0.565273 2.795413 1.523008
H -0.982170 3.516686 0.980667
Ag -0.209627 -1.563978 -0.579286
H -2.341746 -0.623821 1.806022
C -4.155793 0.182942 0.955094
H -3.913505 1.230526 1.156472
H -4.730372 0.140685 0.025734
H -4.800372 -0.171375 1.763059

cis_transition state

C -0.632409 -1.761345 -0.164381
C 0.615140 -2.069056 -0.634624
C 1.831322 -1.991896 0.095612
H 2.683859 -2.375058 -0.454464
H 0.653886 -2.299170 -1.692687
O -0.918414 -1.348965 1.063535
C -2.355952 -1.272359 1.183958
C -2.787419 -1.133688 -0.268853
O -1.696970 -1.790287 -0.950150
C -2.976691 0.295253 -0.784268
O -2.435218 1.311564 0.073555
C -1.160539 1.658362 -0.035020
O -0.403952 1.086954 -0.805662
C -0.805840 2.799364 0.862701
C 0.659369 3.187135 0.829520
H 1.303290 2.380769 1.203803
H 0.981604 3.458232 -0.178594
H 0.833454 4.050113 1.474341
H -1.444123 3.637278 0.558017
H -1.140635 2.540949 1.873050
H -4.040276 0.528751 -0.817972
H -2.551291 0.380060 -1.785880
H -3.679925 -1.715112 -0.498847
H -2.598647 -0.417857 1.811361
H -2.712717 -2.197970 1.638826
Ag 1.711793 0.414544 -0.425394
H 3.063177 -0.412964 -0.044709
C 1.936678 -2.129821 1.580443
H 1.218452 -1.523338 2.128059
H 1.725956 -3.182783 1.813967
H 2.944169 -1.901281 1.924505

cis_product-AgH-complex

C -0.726394 -1.712323 -0.158102
C 0.542355 -2.015278 -0.617638
C 1.729586 -1.961560 0.126709
H 2.613529 -2.279460 -0.418094
H 0.592097 -2.214684 -1.681969
O -1.040735 -1.381457 1.076248
C -2.478545 -1.227166 1.152947
C -2.860188 -1.036042 -0.309252
O -1.750333 -1.681305 -0.979662
C -3.016878 0.406556 -0.787615
O -2.434518 1.394293 0.070510
C -1.133510 1.662407 -0.021538
O -0.398027 0.998954 -0.733082
C -0.745532 2.833077 0.821726
C 0.724370 3.198061 0.758677
H 1.361998 2.405032 1.164162
H 1.042392 3.410037 -0.264689

H	0.910121	4.093093	1.354777
H	-1.377794	3.666863	0.494934
H	-1.071769	2.618397	1.845852
H	-4.075597	0.664819	-0.796353
H	-2.612974	0.496561	-1.798236
H	-3.748279	-1.601816	-0.589719
H	-2.691229	-0.369736	1.787657
H	-2.892381	-2.139588	1.584142
Ag	1.800440	0.298227	-0.415083
H	3.370345	0.211252	-0.086084
C	1.847295	-2.037064	1.612164
H	1.080097	-1.479452	2.145918
H	1.738617	-3.095899	1.888479
H	2.833715	-1.709990	1.937550

cis_ product

C	1.462345	-0.625455	-0.253050
C	2.363658	0.167723	-1.010184
C	3.049461	1.240367	-0.559897
H	3.680577	1.722028	-1.305305
H	2.453780	-0.146197	-2.042813
O	1.188089	-0.456313	0.998514
C	0.273466	-1.501300	1.428354
C	-0.109969	-2.185357	0.115764
O	0.879247	-1.633495	-0.806610
C	-1.526825	-1.948866	-0.404965
O	-2.228222	-0.850327	0.159285
C	-1.853105	0.392560	-0.226351
O	-0.827293	0.583546	-0.829956
C	-2.848808	1.425146	0.191766
C	-2.338917	2.845417	0.035367
H	-1.471169	3.028125	0.673780
H	-2.044899	3.046338	-0.996190
H	-3.118643	3.555986	0.314390
H	-3.743785	1.252291	-0.419313
H	-3.158901	1.201962	1.217783
H	-2.136360	-2.815357	-0.147214
H	-1.482590	-1.858608	-1.494024
H	0.088871	-3.256796	0.131005
H	-0.563072	-1.021106	1.932406
H	0.821142	-2.145496	2.115224
C	3.071937	1.869628	0.772790
H	2.452762	1.380817	1.518955
H	4.109195	1.913536	1.126163
H	2.759071	2.916006	0.667735

trans_transition state

C	0.167307	-1.891683	0.016760
C	1.469441	-1.728353	-0.382605
C	2.442150	-1.134166	0.441076
C	3.886806	-1.285961	0.110694
H	4.086082	-1.126795	-0.949901
H	4.519384	-0.626074	0.701318
H	4.157006	-2.325348	0.345701
H	2.190304	-0.998914	1.488454
H	2.767531	0.886396	0.461109
H	1.704182	-2.023216	-1.398103
O	-0.300831	-1.512378	1.193298
C	-1.660928	-1.984514	1.306941
C	-2.052609	-2.206184	-0.149658
O	-0.760189	-2.400516	-0.768844
C	-2.821527	-1.075165	-0.839861
O	-2.869643	0.157406	-0.108039
C	-1.861340	1.014599	-0.200072
O	-0.845150	0.740925	-0.819769
C	-2.141481	2.296988	0.513973
C	-0.988274	3.281018	0.506069
H	-0.117405	2.892413	1.049665
H	-0.685046	3.538057	-0.511562
H	-1.281544	4.205430	1.006349
H	-3.030647	2.724060	0.034922
H	-2.461339	2.045755	1.531008
H	-3.869652	-1.356528	-0.933219
H	-2.399186	-0.907892	-1.832577
H	-2.595119	-3.140197	-0.294068
H	-2.244145	-1.222691	1.819157
H	-1.653455	-2.911246	1.882787
Ag	1.286857	1.122209	-0.176896

trans_ product-AgH-complex

C	-0.053139	-1.812214	-0.009794
C	1.262441	-1.687690	-0.429852
C	2.280615	-1.210744	0.407877
C	3.718472	-1.363881	0.050617
H	3.896019	-1.257829	-1.020576
H	4.345263	-0.658754	0.593664
H	4.022564	-2.378993	0.342661
H	2.053714	-1.116527	1.468501
H	2.741780	1.584699	0.537269
H	1.459896	-1.952431	-1.462359
O	-0.470222	-1.520946	1.201284
C	-1.873807	-1.861081	1.295256
C	-2.285541	-1.983750	-0.169499
O	-1.005353	-2.205353	-0.815285
C	-3.004681	-0.786274	-0.788209
O	-2.893800	0.442014	-0.063808
C	-1.783572	1.170937	-0.181337
O	-0.808324	0.745899	-0.776211
C	-1.917037	2.502763	0.481800
C	-0.709065	3.405723	0.327670
H	0.177220	2.993331	0.821302
H	-0.469290	3.577764	-0.723812
H	-0.910651	4.374749	0.787284
H	-2.819546	2.964710	0.065654
H	-2.157466	2.316973	1.535378
H	-4.076423	-0.983659	-0.795427
H	-2.655609	-0.653165	-1.814676
H	-2.871443	-2.882224	-0.362621
H	-2.378606	-1.061933	1.834036
H	-1.954633	-2.802263	1.840037
Ag	1.472568	0.851062	-0.125986

trans_ product

C	1.301913	-0.812151	-0.046887
C	2.383161	-0.042241	-0.545924
C	3.067422	0.796305	0.256759

C	4.185431	1.648098	-0.180666
H	4.423764	1.527648	-1.237307
H	3.944068	2.699009	0.020367
H	5.074708	1.429997	0.422712
H	2.776605	0.859743	1.304333
H	2.600576	-0.159070	-1.600755
O	0.896062	-0.762797	1.179589
C	-0.176418	-1.725629	1.358822
C	-0.476227	-2.178324	-0.071438
O	0.678505	-1.651553	-0.798067
C	-1.779760	-1.686521	-0.696786
O	-2.397499	-0.575059	-0.064668
C	-1.833490	0.642272	-0.253879
O	-0.733679	0.761073	-0.732264
C	-2.735377	1.744084	0.199076
C	-2.047433	3.094897	0.257213
H	-1.230517	3.093439	0.982714
H	-1.632509	3.367091	-0.714759
H	-2.760754	3.865686	0.553589
H	-3.580836	1.755232	-0.500368
H	-3.168259	1.453266	1.161832
H	-2.519374	-2.483909	-0.618353
H	-1.597256	-1.475899	-1.754382
H	-0.410852	-3.259794	-0.189142
H	-1.007430	-1.210154	1.836452
H	0.205615	-2.520338	1.998614

PC(3:0/4:0) *cis/trans* isomerization
Figure S12

cis_product

C	1.436795	-0.199895	0.673396
C	1.879404	1.086501	1.068839
C	2.182421	2.102677	0.228189
C	2.141597	2.164170	-1.241252
H	1.719874	1.285651	-1.718316
H	3.161154	2.335736	-1.612043
H	1.570733	3.051505	-1.536831
H	2.488767	3.024020	0.721858
H	1.916795	1.230583	2.141995
O	1.497718	-0.677335	-0.522724
C	0.756022	-1.942490	-0.565703
C	0.662519	-2.285250	0.919520
O	0.940821	-1.008525	1.555508
H	1.421606	-2.988904	1.260793
H	-0.332003	-2.594641	1.233601
C	-0.570587	-1.754072	-1.275986
O	-1.593696	-1.241142	-0.436057
C	-1.718992	0.114105	-0.356532
O	-0.893677	0.854843	-0.820762
C	-2.973498	0.498465	0.359315
C	-3.048418	1.979883	0.677567
H	-2.977391	2.581539	-0.230153
H	-3.995773	2.210089	1.167773
H	-2.239153	2.284001	1.345616
H	-3.055159	-0.124544	1.256191
H	-3.805229	0.183542	-0.283048
H	-0.923146	-2.736610	-1.597328
H	-0.429406	-1.117958	-2.153170
H	1.385417	-2.631306	-1.128778

transition state

C	1.259640	0.177021	0.724322
C	0.954037	1.451496	1.014557
C	0.703295	2.390082	-0.054582
C	1.698480	3.160859	-0.697015
H	2.656743	3.259416	-0.189994
H	1.371224	3.990280	-1.323082
H	1.821573	2.275933	-1.415879
H	-0.311830	2.432876	-0.472845
H	0.726999	1.768722	2.029464
O	1.578198	-0.190783	-0.522972
C	1.389602	-1.618953	-0.615189
C	1.563878	-2.038805	0.845431
O	1.265565	-0.833419	1.577205
H	2.586557	-2.339795	1.077225
H	0.856961	-2.802719	1.165583
C	0.041517	-1.921556	-1.234483
O	-1.031466	-1.609048	-0.348389
C	-1.611085	-0.394176	-0.443768
O	-1.201419	0.473916	-1.176725
C	-2.799701	-0.294447	0.461075
C	-3.261816	1.133135	0.685575
H	-3.488502	1.628715	-0.260157
H	-4.162404	1.146140	1.301651
H	-2.498343	1.718596	1.206643
H	-2.560037	-0.806578	1.397363
H	-3.590575	-0.895205	-0.005382
H	-0.035102	-2.993502	-1.428796
H	-0.067791	-1.370311	-2.170794
H	2.180413	-2.005831	-1.259487

trans_product

C	0.302406	-1.475081	0.423196
C	1.690427	-1.270515	0.612264
C	2.462988	-0.893424	-0.430071
C	3.898554	-0.590453	-0.342123
H	4.305223	-0.718108	0.660910
H	4.072386	0.437235	-0.684228
H	4.449480	-1.227327	-1.045260
H	1.988058	-0.776227	-1.402495
H	2.062315	-1.362850	1.625939
O	-0.248466	-1.566126	-0.739794
C	-1.700623	-1.478271	-0.580843
C	-1.858301	-1.765393	0.914073
O	-0.510836	-1.563900	1.423711
H	-2.140227	-2.792981	1.142047
H	-2.504741	-1.056443	1.427537
C	-2.165832	-0.110452	-1.030048
O	-1.822504	0.893855	-0.087652
C	-0.672306	1.598698	-0.311101
O	0.106069	1.293368	-1.172413
C	-0.555513	2.744040	0.643592
C	0.823928	3.376142	0.645944
H	1.099818	3.721484	-0.351751

H	0.844083	4.231559	1.323164
H	1.585137	2.665359	0.977799
H	-0.856115	2.394275	1.636384
H	-1.324919	3.469011	0.349821
H	-3.256948	-0.117469	-1.089915
H	-1.746983	0.117749	-2.012732
H	-2.125040	-2.254591	-1.217330

References

[1] Pracht P, Bohle F, Grimme S. Automated exploration of the low-energy chemical space with fast quantum chemical methods. Phys Chem Chem Phys. 2020;22:7169-92.

[2] Bannwarth C, Ehlert S, Grimme S. GFN2-xTB-An Accurate and Broadly Parametrized Self-Consistent Tight-Binding Quantum Chemical Method with Multipole Electrostatics and Density-Dependent Dispersion Contributions. J Chem Theory Comput. 2019;15:1652-71.

[3] Frisch MJ, Trucks GW, Schlegel HB, Scuseria GE, Robb MA, Cheeseman JR, Scalmani G, Barone V, Petersson GA, Nakatsuji H, Li X, Caricato M, Marenich AV, Bloino J, Janesko BG, Gomperts R, Mennucci B, Hratchian HP, Ortiz JV, Izmaylov AF, Sonnenberg JL, Williams, Ding F, Lipparini F, Egidi F, Goings J, Peng B, Petrone A, Henderson T, Ranasinghe D, Zakrzewski VG, Gao J, Rega N, Zheng G, Liang W, Hada M, Ehara M, Toyota K, Fukuda R, Hasegawa J, Ishida M, Nakajima T, Honda Y, Kitao O, Nakai H, Vreven T, Throssell K, Montgomery Jr. JA, Peralta JE, Ogliaro F, Bearpark MJ, Heyd JJ, Brothers EN, Kudin KN, Staroverov VN, Keith TA, Kobayashi R, Normand J, Raghavachari K, Rendell AP, Burant JC, Iyengar SS, Tomasi J, Cossi M, Millam JM, Klene M, Adamo C, Cammi R, Ochterski JW, Martin RL, Morokuma K, Farkas O, Foresman JB, Fox DJ. Gaussian 16, Rev. A.03. 2016.

[4] Adamo C, Barone V. Toward reliable density functional methods without adjustable parameters: The PBE0 model. J Chem Phys. 1999;110:6158-70.

[5] Grimme S, Antony J, Ehrlich S, Krieg H. A consistent and accurate ab initio parametrization of density functional dispersion correction (DFT-D) for the 94 elements H-Pu. J Chem Phys. 2010;132:154104.
